# Isolation of Oral Bacteria, Measurement of the C-Reactive Protein, and Blood Clinical Parameters in Dogs with Oral Tumor

**DOI:** 10.1155/2023/2582774

**Published:** 2023-03-22

**Authors:** Chanokchon Setthawongsin, Duangdaow Khunbutsri, Sirinun Pisamai, Wuttinun Raksajit, Suchanit Ngamkala, Thitichai Jarudecha, Nattakan Meekhanon, Anudep Rungsipipat

**Affiliations:** ^1^Department of Veterinary Technology, Faculty of Veterinary Technology, Kasetsart University, Bangkok10900, Thailand; ^2^Veterinary Diagnostic Laboratory, Faculty of Veterinary Medicine, Khon Kaen University, Khon Kaen40002, Thailand; ^3^Department of Veterinary Surgery, Faculty of Veterinary Science, Chulalongkorn University, Bangkok10330, Thailand; ^4^Center of Excellence for Companion Animal Cancer, Department of Veterinary Pathology, Faculty of Veterinary Science, Chulalongkorn University, Bangkok10330, Thailand

## Abstract

Canine oral cancers have a poor prognosis and are related to chronic inflammation. This may pose a risk of secondary bacterial infection. This study aimed to compare the bacteria isolated from oral swab samples, values of C-reactive proteins (CRPs), and clinical blood profiles of dogs with and without oral mass. A total of 36 dogs were divided in three groups: no oral mass (*n* = 21), oral mass (*n* = 8), and metastasis groups (*n* = 7). Significantly, both the clinical groups (the oral mass group and metastasis group) showed anemia, a decrease in the albumin-to-globulin ratio (AGR), and an increase in the neutrophil-to-lymphocyte ratio (NLR), globulin-to-albumin ratio (GAR), CRP, and CRP-to-albumin ratio (CAR) compared to the normal group. CAR showed an increasing trend in the oral mass and metastasis groups (10 times and 100 times, respectively) compared to the no oral mass group (*P* < 0.001). *Neisseria* spp. (20.78%) was the main isolated bacteria in all groups. The main genera in the no oral mass group were *Neisseria* spp. (28.26%), *Pasteurella* spp. (19.57%), and *Staphylococcus* spp. (19.57%). *Neisseria* spp., *Staphylococcus* spp., *Klebsiella* spp., and *Escherichia* spp. were found equally (12.5%) in the oral mass group. *Escherichia* spp. (26.67%), *Pseudomonas* spp. (13.33%), and *Staphylococcus* spp. (13.33%) were the main genera in the metastasis group. Interestingly, *Neisseria* spp. decreased in the clinical groups (Fisher's exact = 6.39, *P*=0.048), and *Escherichia* spp. increased in the metastasis group (Fisher's exact = 14.00, *P*=0.002). The difference of oral bacteria in clinical dogs compared to healthy dogs may be related to microbiome alterations, and both the clinical groups showed the increment of inflammatory biomarkers. This suggested that further studies should be conducted on the correlation between the specific bacteria, CRP, blood clinical parameters, and type of canine oral mass.

## 1. Introduction

Nowadays, cancer is the most common diseases in both dogs and human. The dogs and their owners share the same environment [1]. Many studies revealed that they response to the carcinogens from the environment in the same manner. For example, tobacco smoke was related to the increasing incidence of the upper respiratory tract cancer and oral cancer [2]. Head and neck cancers (HNC), especially oral cancers, are often classified as serious diseases in dogs. The most common type of oral cancer in dogs is oral melanoma which is followed by oral squamous cell carcinoma (SCC). Oral melanoma in human presents the high aggressive behavior. Canine oral melanoma shares the similar features and characteristics with human oral melanoma. It has been argued that the spontaneous oral cancer in dogs may be a good model and the comparative study for human cancer [1]. Benign tumors, such as fibromatous epulis, ossifying epulis, and acanthomatous ameloblastoma, show slow progress and are curable by excision surgery [3, 4]. On the other hand, oral melanoma and SCC show aggressive behavior, a high metastasis rate, poor prognoses, and short survival times [5, 6].

In human research, chronic inflammation of oral cancer has been observed. Most of them trigger inflammatory responses and induce chronic inflammation status. Tumor-promoting inflammation may play an important role in steps of cancer progression as mentioned by Hanahan and Weinberg [7]. An increase in clinical blood parameters related to inflammation may indicate the existence of cancer or tumors. The concentration of the C-reactive protein (CRP), an acute phase protein, is rapidly rising in response to trauma [8], inflammation [9–11], infection [12–16], and several malignancies [8, 17–19]. The elevated CRP levels are the essential information for diagnosis and prognosis not only in human medicine [20–22] but also in veterinary clinical medicine [16, 17, 23–27]. A previous report identified CRP and serum amyloid A (SAA) as diagnostic markers and prognostic indications after treatment of inflammation for the bacterial pneumonia [15]. CRP, SAA, and haptoglobin were detected and a significant increase in the canine mammary cancer such as anaplastic carcinoma, complex adenocarcinoma, simple adenocarcinoma, and SCC with metastasis, which characterized in the clinical stage IV–V or by a mass diameter greater than 5 centimeters with ulceration and secondary inflammation, was seen. Conversely, these dogs had the decreasing albumin concentration [17]. Moreover, canine mammary carcinoma showed a high concentration of CRP. This suggested that an inflammatory response is associated with this type of cancer [27]. In addition to mammary cancer, elevated concentration of CRP was detected in hemangiosarcoma, nasal adenosarcoma, cholangiocellular carcinoma, acute lymphoblastic leukemia, malignant histiocytosis, lymphoma, malignant mesothelioma [26], and neuroendocrine carcinoma [28]. The *α*_1_-acid glycoprotein (AAG) and CRP were increased after operations to remove malignant cancer from the dogs and these acute-phase proteins again increased further after recurrences and metastasis [8].

Canine oral microflora comprised numerous resident bacteria [29–33]. These bacteria communities can play an essential role in interactions with host immunity [34]. The population and ecological system of the oral microbial agents can be changed as the host's health changes [34–38]. Many studies reported that the microbiome could either directly or indirectly increase the chances of developing cancers in human and animal specific pathogen models. For example, the formation of mice colon cancer and hepatocellular carcinoma is promoted by *Helicobacter hepaticus* infection [39, 40]. In addition, chronic infection of enteropathogenic *Escherichia coli* can result in colon cancer [41]. The specific bacterial pathogen promotes tumor formation and progression that is the principal mechanism of microbiota related to human SCC, gastric cancer, colorectal cancer, and melanoma [34, 38, 41–44]. Recently, the impact of microbiome related to the process of cancer development was summarized as a new hallmark of cancer [45]. Canine oral cancers with progression and chronic inflammation can break or decrease the host's oral mucosal defenses, and the failure of the host barrier may increase the risk of dysbiosis or the development of persistent or recurrent bacterial infection. This might be the result from persistent mucosal and/or epithelial cell colonization by microorganisms [46]. The advancement in bacterial identification with polymerase chain reaction (PCR) amplified 16S sequences. Dewhirst et al. found that the bacterial content in the dog mouth differs from that in the human mouth, and there were only 16.4% shared bacterial types in the dogs and human [47]. Ruparell et al. compared the microbiota from different areas within the canine oral cavity and found three niches (soft tissue surface, hard tissue surface, or dental plaque and saliva) in which the bacterial community profiles differed [33]. deCarvalho et al. compared the oral swabs from the normal dogs and the canine oral melanoma dogs. They found that *Tannerella forsythia* and *Porphyromonas gingivalis* from the subgingival plaque samples were significantly increased in canine oral melanoma dogs compared to the control dogs. These bacterial species are related to the periodontal diseases and human esophageal cancer [48]. So far, there are few studies that compare the oral bacteria of healthy dogs and dogs with oral cancer. The information of oral bacteria in canine oral tumors is limited and unclear. More information and knowledge about the bacterial population and the microbial alteration in the dog's mouth and the clinical blood profiles might help us to increase the understanding about the role of oral bacteria and the blood profile in canine oral tumor and cancer.

For providing more information of canine oral cancer, this study investigated the oral swab bacteria using the culture dependent method with 16s rRNA gene sequencing identification and the clinical blood profiles of the dogs with and without oral mass. We focused on identifying culturable bacteria living in the oral mucosal surface of dogs. Thus, the purposes of our study were to compare the bacteria isolated from canine oral swab samples, values of C-reactive proteins (CRPs), and clinical blood profiles of dogs with and without oral mass.

## 2. Materials and Methods

### 2.1. Animals and Sample Collections

**Figure 1:**
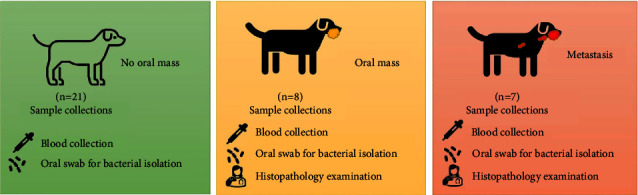
The groups of animals in this study and techniques of sample collections. There was one normal group,the no oral mass group, and the two clinical groups, the oral mass group and the metastasis group.

The thirty-six dogs in this observational prospective study were invited from the private animal hospitals in Bangkok and vicinity between June 2019 and March 2021. All the dog owners gave informed consent for sample collections from their dogs to be used for research purposes only (ACKU62-VTN-010). The signalment information included sex, age, and breed that were recorded. The dogs that had visited the small animal hospitals for the routine annual vaccination program and whose physical examination revealed no evidence of oral mass or serious clinical illness were included in this study as the “no oral mass” group or normal group (*n* = 21). In this group, there were 9 male and 12 female dogs. Their ages were between 6 and 13 years. The clinical group or the “oral tumor-bearing” group included dogs that presented with mass in their mouths during physical examination (*n* = 15) and/or had a later appointment for the surgical removal of an oral mass. Based on the definitive diagnosis from histopathological examination and clinical staging of fifteen dogs in the clinical group, eight dogs were classified in the oral mass group and seven dogs were classified in the metastasis group. In both clinical groups, there were 8 male and 7 female dogs. Their age was between 4 and 16 years (Table 1) (Figure 1).

All owners were asked to feed their dogs with commercial diets free of raw meat or milk products. The dog's diets were managed to prevent oral contamination from raw food and fecal matter. An oral swab sample was collected from the tongue's dorsum mucosa and the mucosa of the hard palate of each dog. The oral swab sampling was performed after 12 hours of fasting. The swabs were then transported in the Stuart transport medium (Yangzhou Chuangxin medical device factory, Yangzhou city, Jiangsu, China) from the animal hospital to the faculty of Veterinary Technology, Kasetsart University under cold transport, 4°C. Then, the bacterial culture was performed. Blood samples were collected and kept in the EDTA tube and Heparin tube for hematology and blood chemistry profile analysis, respectively.

In the oral mass group and the metastasis group, the oral swab, blood collection, and excision or incision biopsy were performed under anesthesia. The tissue from the oral mass was sent to the veterinary pathologists for diagnosis, and finally, a histopathological definitive diagnosis was received.

### 2.2. Clinical Laboratory Examination

The medical records include age, gender, clinical signs, histopathological diagnosis, complete blood count (CBC), the blood urea nitrogen (BUN) level, the creatinine (CRE) level, the alanine aminotransferase (ALT) level, the alkaline phosphatase (ALKP) level, total protein (TP), albumin concentration (ALB), globulin concentration (GLOB), and CRP concentration.

The CBC results were analyzed using an automatic analyzer (ProCyte Dx Hematology Analyzer, IDEXX Laboratories, Inc. USA). The BUN, CRE, ALT, ALKP, TP, ALB, and GLOB levels were measured using automatic blood chemistry measurement equipment (Catalyst One Chemistry Analyzer, IDEXX Laboratories, Inc., USA).

The plasma CRP concentration was measured using a commercial fluorescent immunoassay (Vcheck Canine CRP 2.0 Test Kit, Bionote, Gyeonggi-do, South Korea). In brief, five microliters of each plasma sample from all dogs were diluted with the diluent buffer (to 100 *μ*l), mixed thoroughly, and aliquoted to the test device (V200 Analyzer, Bionote, South Korea). The plasma CRP concentrations were recorded for further statistical analysis. As part of routine diagnosis, CRP concentration results below the detection limit for the assay were set as less than 10 mg/L.

### 2.3. Bacterial Culture, Isolation, and Identification

An oral swab was cultured on blood agar (BA) and MacConkey agar at 37°C for 24–48 hours. All different predominant colonies that grew in the last plane of agar were selected. The predominant selected morphology colony obtained from each sample was further cultured on BA to obtain a pure culture. Genomic DNA from each pure isolate was extracted using E.Z.N.A.® Bacterial DNA kit (Omega Bio-Tek, Doraville, GA, USA) following the manufacturer's instructions. The extracted bacterial DNA of isolates was identified using 16S rRNA gene sequencing by U2Bio Thailand (Bangkok, Thailand) and Bionics Co. Ltd. (Seoul, Korea). The extracted bacterial DNA was prepared for the identification step using 16S rRNA gene sequencing. The 16S rRNA gene was amplified with the pair of primers 518F (5′-CCAGCAGCCGCGGTAATACG-3′) and 800R (5′-TACCAGGGTATCTAATCC-3′). The thermocycler conditions were a predenaturation at 94°C for 4 min; 25 cycles of 94°C for 20 sec, 50°C for 40 sec, 72°C for 90 sec; a final extension step at 72°C for 3 min, and hold temperature at 4°C. PCR product was generated. Then, sequencing was performed after purification with ABI3730XL Sequencer (Thermo Fisher Scientific, Massachusetts, USA) by the Sanger sequencing method.

The nucleotide sequences were processed and assembled using BioEdit and the contig assembly program. The percent identity of bacterial isolates was determined using the BLAST server (https://blast.ncbi.nlm.nih.gov/Blast.cgi) of the National Center for Biotechnology Information (NCBI). The highest score and the closest relatives of the 16S rRNA gene were evaluated. Similarities to 16S rRNA gene sequences of the isolates that were ≥99% were used as criteria for identification.

### 2.4. Statistical Analysis

In this study, a variable was considered as a normal distribution when the *P* value of the Shapiro‐Wilk test was larger than 0.05. The clinical blood profile data were showed as mean ± standard deviation (SD) when they were the normal distribution or showed as median with the interquartile range (IQR) when they were the non-normal distribution. Continuous variables in the normal distribution (ALB, AGR, and CRE) among three groups were compared using a one-way ANOVA test. Post hoc multiple comparisons were performed using the Bonferroni test. The non-normal distribution of continuous variables (RBC, WBC, PLT, NLR, TP, GLOB, GAR, CRP, CAR, BUN, ALT, and ALKP) among three groups was analyzed using the Kruskal–Wallis test. Differences in the two groups were analyzed by the Mann–Whitney *U* test. The oral bacterial isolates were presented on percentage. The Fisher's exact was used to assess the relationships of variable-bacterial profiles to the groups of dogs. Multinomial logistic regression analysis was used to estimate the relative risk ratio (RRR) among the groups of variables. All data were facilitated by the STATA statistics analysis (StataCorp LLC, Texas, USA; serial number 401506228202), and *P* < 0.05 was considered significant. The graphs of variables were created using the GraphPad prism program (GraphPad Software, San Diego, USA) and Microsoft Excel program (Microsoft 365, Washington, USA).

## 3. Result

### 3.1. Animals

Thirty-six dogs were included in this study. Twenty-one dogs (9 males and 12 females) were classified into the group of no oral mass dogs. They were 6–13 years old. In the two clinical case groups, there were 15 oral tumor-bearing dogs (8 male and 7 female). The age of dogs in this study was between 4 and 16 years old (Table 1). The increase of age did not increase the risk ratio in the oral mass group (RRR = 1.11, 95% CI: 0.84–1.48) and the metastasis group (RRR = 1.38, 95% CI: 0.98–1.95) when evaluated based on the no oral mass group (*P* > 0.05) (Table 2). The proportion of female and male dogs that had oral masses or metastasis did not differ when evaluated based on the no oral mass group (*P* > 0.05) (Table 2).

**Figure 2:**
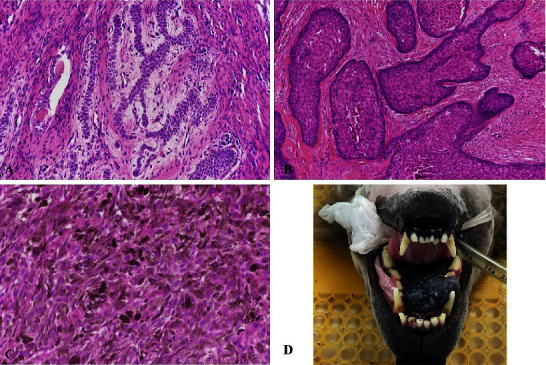
Histopathology of oral tumor mass stained with hematoxylin and eosin (H&E) stain and gross lesion of canine oral mass in this study. This figure presents the histopathology of acanthomatous ameloblastoma (20x,(a)), squamous cell carcinoma (SCC, 20x,(b)), malignant melanoma cells metastasis to the regional lymph node (40x,(c)), and the gross lesion of oral mass at the maxilla area (d).

**Figure 3:**
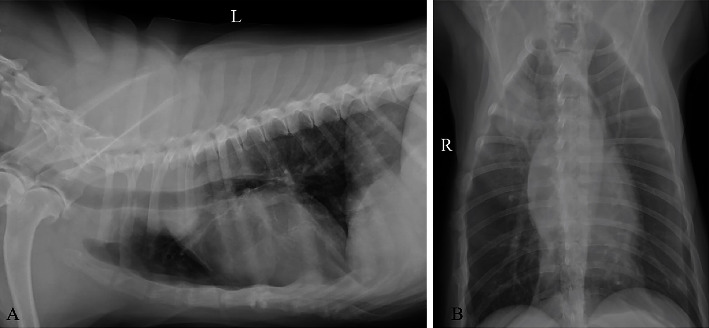
The thoracic radiography of the oral tumor-bearing dog with lung metastasis. The left lateral view of thoracic radiography (a) and the ventrodorsal (VD) view of thoracic radiography (b).

In the oral mass group, there were two cases of benign oral tumors in clinical stage I, which were acanthomatous ameloblastoma (*n* = 2), and six cases of malignant oral tumor with clinical stage II and III which were oral malignant melanotic melanoma (*n* = 1), oral SCC (*n* = 2), oral fibrosarcoma (*n* = 1), and oral malignant amelanotic melanoma (*n* = 2) (Table 3) (Figures 2(a) and 2(b)). In the metastasis group with clinical stage IV (regional lymph node metastasis and/or pulmonary metastasis), there were oral malignant melanotic melanoma (*n* = 3), oral chondrosarcoma (*n* = 1), oral squamous cell carcinoma (*n* = 1), and oral fibrosarcoma (*n* = 2) (Table 4) (Figures 2(c), 2(d), 3(a), and 3(b)). The dogs in the clinical case groups showed the clinical signs related to the oral mass such as hypersalivation, halitosis, dysphagia, and bleeding from mass. There was a dog (Case 1) in the metastasis group that presented lethargy and panting sign.

### 3.2. Clinical Laboratory Results

**Figure 4:**
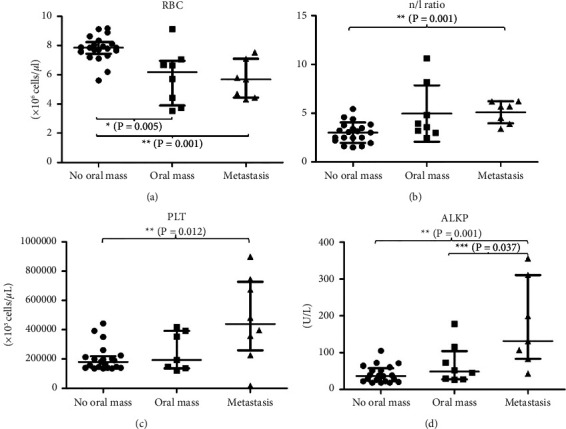
Comparison of red blood cell (RBC, median (IQR))(a), the neutrophil/lymphocyte ratio (n/l ratio or NLR, median (IQR))(b), platelet counts (PLT, median (IQR))(c), and alkaline phosphatase (ALKP, median (IQR))(d) of dogs among the no oral mass group, the oral mass group, and the metastasis group.

According to the CBC results, the median RBC in the oral mass group and metastasis group decreased when compared with the no oral mass group (*P* < 0.001) (Figure 4(a) and Table 5). The dogs in both clinical groups exhibited anemia. The proportion of dogs that had low RBC levels in the oral mass group (RRR = 12.74, 95% CI: 1.03–157.02) and the oral mass with the metastasis group (RRR = 12.74, 95% CI: 1.03–156.98) were more than the no oral mass group (*P*=0.047, borderline significance) (Table 6). There was a trend toward an increase in WBC in the clinical groups (*P*=0.088). Moreover, the NLR in both the clinical groups showed a trend to increase when compared to the no oral mass group, and it was significantly increased in the metastasis group when compared to the no oral mass group (*P*=0.001) (Figure 4(b) and Table 5). The median of platelets in the metastasis group was significantly higher than in the no oral mass group (*P*=0.012) (Figure 4(c) and Table 5). Only the metastasis group showed an increasing level of platelets compared to the normal reference range. The clinical blood chemistry profiles of the three groups were in the normal reference range. However, the median plasma ALKP in the metastasis group was significantly higher than those in the no oral mass group (*P*=0.001) and the oral mass group (*P*=0.037) (Figure 4(d), Table 5).

**Figure 5:**
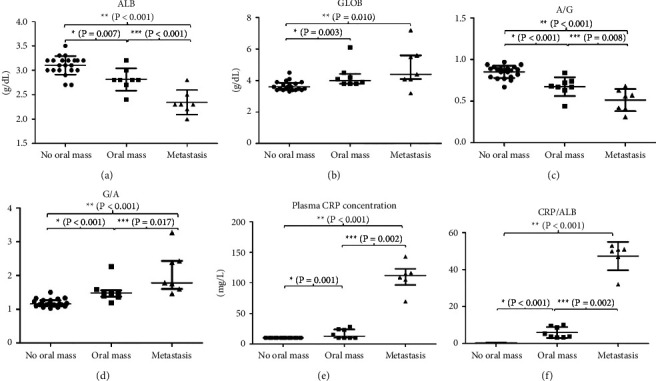
Comparison of the plasma albumin concentration (ALB, mean ± SD)(a), plasma globulin concentration (GLOB, median (IQR))(b), albumin to globulin ratio (A/G, AGR mean ± SD)(c), globulin to albumin ratio (G/A, GAR, median (IQR))(d), plasma CRP concentration (CRP, median (IQR))(e), and CRP to ALB ratio (CRP/ALB, CAR median (IQR))(f) of dogs among the no oral mass group, the oral mass group, and the metastasis group.

Plasma TP, ALB, GLOB, CRP, AGR, GAR, and CAR are presented in Table 5 and Figure 5. The protein level in all groups was in the normal reference range. The TP was not different among the three groups of dogs (Table 5). ALB and GLOB showed the opposite trend. The ALB showed decreased levels in both the clinical groups; conversely, the GLOB showed increased levels in both the clinical groups (Figures 5(a) and 5(b)). The mean ALB and the median of the AGR in the two clinical groups were significantly lower than in the no oral mass group (*P* < 0.001) (Figures 5(a) and 5(c)). Moreover, AGR in the metastasis group was significantly lower than the oral mass group (*P* = 0.008) (Figure 5(c)). Conversely, the median of plasma GLOB levels in the oral mass group (*P* = 0.003) and the metastasis group (*P* = 0.01) were significantly higher than the no oral mass group (Figure 5(b)). GAR in the oral mass group (*P* < 0.001) and the metastasis group (*P* < 0.001) was significantly higher than the no oral mass group. In addition, GAR in the metastasis group was significantly higher than the oral mass group (*P* = 0.017) (Figure 5(d)). All dogs in the no oral mass group had a CRP level of less than 10 mg/L, which is under the normal range or less than 20 mg/L. In the oral mass group, the median CRP level was 12.45 mg/L and the range was between less than 10 mg/L and28.2 mg/L. In clinical stage I and II, most dogs had CRP levels of less than 10 mg/L. They had the CRP level higher than 20 mg/L in clinical stage II. Most of the dogs in the metastasis group (clinical stage IV) had a plasma CRP level higher than 50 mg/L which is higher than the normal range. The median plasma CRP concentrations of the oral mass group and the metastasis group were 12.45 and 112 mg/L, respectively, and were significantly higher than those of the no oral mass group (*P* < 0.001) (Figure 5(e), Table 5). Also, the CRP levels of the metastasis group were significantly higher than those of the oral mass group (*P* = 0.002) (Figure 5(e) and Table 5). However, one dog in the metastasis group received a nonsteroidal anti-inflammatory drug before the blood collection, and the CRP level of this dog was less than 10 mg/L. So, the CRP level and CAR data of this dog were not included for the statistical analysis. The median CRP and CAR in the oral mass group and the metastasis group were significantly higher than in the no oral mass group (*P* < 0.001) (Figures 5(e) and 5(f) and Table 5). The median CRP of the metastasis group was about 10 times higher than that of the no oral mass group. In addition, the median of the CAR of the oral mass and metastasis groups was higher than that of the no oral mass group about 10–15 times and 100 times, respectively.

### 3.3. Bacterial Isolation and Identification

**Figure 6:**
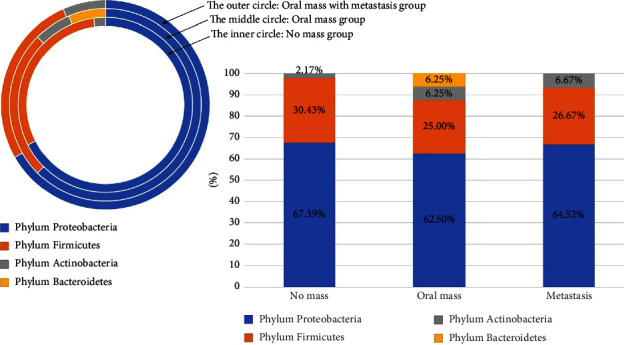
Phylum of the bacterial isolates which were analyzed based on 16S rRNA gene taxonomy from the canine oral swabs in the three groups of dogs.

**Figure 7:**
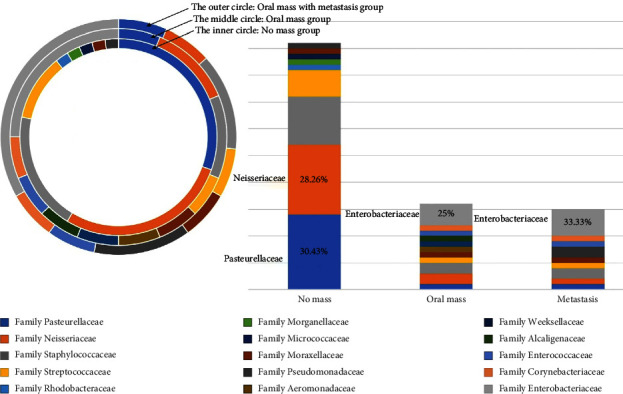
Family of the bacterial isolates which were analyzed based on 16S rRNA gene taxonomy from the canine oral swabs in the three groups of dogs.

Among a total of 36 oral swab samples, 77 bacterial isolates were analyzed based on 16S rRNA gene taxonomy (Table 7) (Supplementary file 1-2). There were four bacterial phyla: Proteobacteria (51/77, 66.23%), Firmicutes (22/77, 28.57%), Actinobacteria (3/77, 3.70%), and Bacteroidetes (1/77, 1.30%). Proteobacteria was the majority phylum of isolated bacteria in all groups: the no oral mass group (31/46, 67.39%), the oral mass group (10/16, 62.50%), and the metastasis group (10/15, 64.52%) (Figure 6). In our study, there were nine families in the Proteobacteria: Rhodobacteraceae, Neisseriaceae, Alcaligenaceae, Aeromonadaceae, Enterobacteriaceae, Morganellaceae, Pasteurellaceae, Moraxellaceae, and Pseudomonadaceae. Pasteurellaceae (14/46, 30.43%) and Neisseriaceae (13/46, 28.26%) were the two main bacterial families in the no oral mass group (Figure 7). We found that the bacterial isolates of family Pasteurellaceae in the oral mass group (Fisher's exact = 6.807, *P* = 0.014) (OR = 0.071, 95%CI: 0.007–0.70, *P* = 0.023) and the metastasis group (Fisher's exact = 5.79, *P* = 0.029) (OR = 0.083, 95%CI: 0.008–0.833, *P* = 0.034) were significantly lower than the no oral mass group. Moreover, the bacterial isolates of the Neisseriaceae family in the clinical groups showed a lower trend than in the no oral mass group (Fisher's exact = 6.39, *P* = 0.048, borderline significance). Interestingly, Enterobacteriaceae was the majority bacterial family of both clinical cases groups, which were the oral mass group (4/16, 25%) and the metastasis group (5/15, 33.33%) (Figure 7). This family was not isolated from the no oral mass group. From our result, the bacterial isolates of Enterobacteriaceae family showed the higher evidence in the oral mass group (Fisher's exact = 12.18, *P* = 0.003) and the metastasis group (Fisher's exact = 18.26, *P* < 0.001) compared with the no oral mass group. Also, the bacterial isolates of Alcaligenaceae, Aeromonadaceae, Enterococcaceae, and Corynebacteriaceae families were identified from the two clinical case groups (Figure 7).

**Figure 8:**
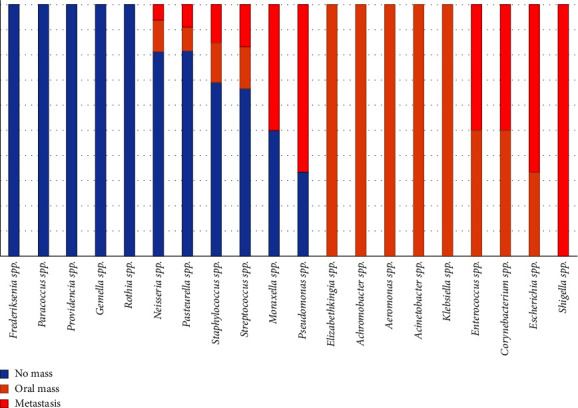
Genus of the bacterial isolates which were analyzed based on 16S rRNA gene taxonomy from the canine oral swabs in the three groups of dogs.

In the present study, the bacterial isolates were identified into 20 genera: *Frederiksenia* spp., *Paracoccus* spp., *Providencia* spp., *Gemella* spp., *Rothia* spp., *Neisseria* spp., *Pasteurella* spp., *Staphylococcus* spp., *Streptococcus* spp., *Moraxella* spp., *Pseudomonas* spp., *Elizabethkingia* spp., *Achromobacter* spp., *Aeromonas* spp., *Acinetobacter* spp., *Klebsiella* spp., *Enterococcus* spp., *Corynebacterium* spp., *Escherichia* spp., and *Shigella* spp. (Figure 8). The three main bacterial genera in all groups were *Neisseria* spp. (16/77, 20.78%), *Staphylococcus* spp. (13/77, 16.88%), and *Pasteurella* spp. (11/77, 14.29%). Interestingly, *Neisseria* spp. was the main genus of bacterial isolates from all the groups. In the no oral mass group, the three majority genera of bacterial isolates were *Neisseria* spp. (13/46, 28.26%), *Pasteurella* spp. (9/46, 19.57%), and *Staphylococcus* spp. (9/46, 19.57%). Bacterial isolates from the genus *Frederiksenia* spp., *Paracoccus* spp., *Providencia* spp., *Gemella* spp., and *Rothia* spp. were found only in the no oral mass group (Figure 8).

**Figure 9:**
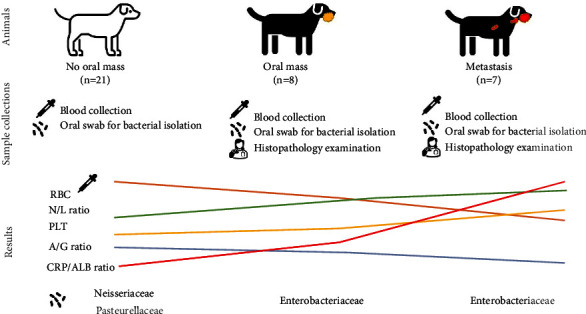
The layout of animals, sample collections, and the interesting results in this study.

In the oral mass group, there were four main genera of bacterial isolates: *Neisseria* spp. (2/16, 12.5%), *Staphylococcus* spp. (2/16, 12.5%), *Klebsiella* spp. (2/16, 12.5%), and *Escherichia* spp. (2/16, 12.5%). *Pasteurella* spp., *Streptococcus* spp., *Elizabethkingia* spp., *Achromobacter* spp., *Aeromonas* spp., *Acinetobacter* spp., *Enterococcus* spp., and *Corynebacterium* spp. were also isolated from the oral mass group. In the metastasis group, the three main majority genera of bacterial isolates were *Escherichia* spp. (4/15, 26.67%), *Pseudomonas* spp. (2/15, 13.33%), and *Staphylococcus* spp. (2/15, 13.33%). *Aeromonas* spp., *Acinetobacter* spp., *Klebsiella* spp., and *Shigella* spp. were also isolated from the metastasis group. There were three genera of bacterial isolates which were *Enterococcus* spp., *Corynebacterium* spp., and *Escherichia* spp. found in both the clinical groups (Figure 8). Moreover, *Shigella* spp. was isolated from only the metastasis group. Based on our results, *Neisseria* spp. isolates decreased in the clinical groups (Fisher's exact = 6.39, *P* = 0.048, borderline significance). Interestingly, *Escherichia* spp. was found in samples from the clinical groups, and we did not find evidence of this genus in the no oral mass group. This difference between the no oral mass group and the clinical groups was confirmed to have statistical significance (Fisher's exact = 12.857, *P* = 0.001). *Escherichia* spp. showed significantly higher evidence in the metastasis group (Fisher's exact = 14.00, *P* = 0.002) compared with the no oral mass group. Finally, the summary figure of the animal groups, the sample collections, and the interesting results of this study were provided in Figure 9.

## 4. Discussion

The present study aimed to compare blood profiles and plasma CRP levels among the no oral mass group and the two clinical groups. Both the clinical groups, the oral mass group and the metastasis group, showed a significant decrease in total RBC when compared with the no oral mass group. This finding may imply that most of the severe tumor-bearing dogs showed the anemia status which is related to the mass bleeding. Moreover, the growing mass in the oral cavity may have interfered with the dog's nutrition consumption. This status relates to the anemia of inflammatory disease (AID) which is the mild to moderate severity, nonregenerative anemia in chronic diseases including the neoplasia [49]. Previous reports have claimed that inflammation plays a key role in this type of anemia. Shortening RBC life span, impaired erythropoietin-mediated erythropoiesis, and inhibition of iron metabolism are the clinicopathological features [49]. The point is that if the oral mass cannot be treated, alternative treatments should be performed for these dogs such as blood transfusion in severe anemia cases, parenteral iron therapy, and the administration of recombinant human erythropoietin in mild to moderate anemic dogs.

According to the CBC results, there was a trend toward increasing WBC and the NLR in the oral mass group and the metastasis group compared to the no oral mass group. This implies that the tumor-bearing group displayed the inflammation feature. Many bacterial isolates in our study were the gram-negative bacteria. Gram-negative bacteria and their endotoxins, lipopolysaccharide (LPS), play the important role of inflammatory stimuli that are activated through the inflammatory cytokines [46, 50]. In addition, lipoteichoic acid (LTA) of Gram-positive bacteria is also one of the antigens that activated the immune cells [34]. The chronic inflammation, loss of surface integrity in the oral mucosal epithelium, and structure of the oral cancer in patients may result from the persistent bacterial colonization of mucosa or cancer epithelial cells, which has been observed at various stages of oral cancer. Thus, chronic inflammation could result from persistent mucosal or epithelial cell colonization by microorganisms. There is a lot of evidence of oral bacteria that are involved in the inflammation [2, 46, 51].

In this study, the CRP level of both the clinical groups was significantly higher than those of the no oral mass group and the increasing of the CRP level was related to the severity clinical stage. Cancer and inflammation are associated in a way that some cancers arise at the sites whereas the cancer induces an inflammatory microenvironment. We could support that CRP is a sensitive marker that has been shown to increase in response to malignancies [19, 26] such as lymphoma [18, 52], multiple myeloma [53], pancreatic cancer [54], and SCC [55]both in humans and dogs. In most studies, CRP levels were found to be highly elevated in patients with cancer and metastasis compared with the healthy control group or benign conditions [26, 27, 56]. These CRP-related inflammatory evidence may be the potential role of the tissue injuries which related to the tumor consequence of inflammatory response. The role of inflammation in the tumor development and progression was mentioned and added to the new version of hallmarks of cancer in human by Hanahan [45].

One dog with oral malignant melanoma that had regional lymph node and lung metastasis (clinical stage IV) and a plasma CRP level of 115 mg/L, died within seven days of the first visit. Increasing CRP levels and blood profiles showed a severe leukocytosis inflammatory pattern. The critical CRP value was mentioned in the systemic inflammatory response or severe emergency cases [24, 27]. According to our results, the CRP level in a high clinical stage presented as greater than 100 mg/L, as in previous studies. Elevated CRP is likely a response secondary to tumor necrosis and local tissue damage; this response may be attributable to bacterial translocation from the damaged oral mucosa, endothelium, and resulting septicemia that is associated with inflammation in patients with malignancies. NSAIDs inhibit the cyclooxygenase enzymes and anti-inflammation [57]. NSAIDs were the clinical factor for the dog that had a CRP level in the normal range.

In the oral mass with the metastasis group, the CRP and ALKP enzyme levels were significantly high. It is a fact that hepatocytes are the main cells that produce and release CRP to the systemic circulation. This process occurs in response to increased levels of circulating proinflammatory cytokines [58, 59]. Therefore, the plasma CRP and ALKP levels may be correlated with proinflammatory mediators, especially interleukin-1 (IL-1) and interleukin-6 (IL-6) in liver cells [58–60]. There are three isoforms of ALKP: liver ALKP (LALKP), bone ALKP (BALKP), and corticosteroid-induced ALKP (CALKP). ALKP is the primary indicator of cholestatic liver disease. This enzyme also increases with severe bone destruction and steroid induction. The increase in BALKP, which is shown in the total ALKP, is usually found in young puppies or during times of active osteoblast and bone development [61]. In our study, the metastasis dogs had increased ALKP levels. Their abdominal ultrasound examination results did not show any alteration of the liver and/or bile duct structure. The alteration of bone or bone lytic condition due to the cancer metastasis may be the cause of the increasing ALKP level in the malignant part with metastasis group compared to the no oral mass group and the oral mass without metastasis group. However, we cannot conclude that the total ALKP increase is due to bone destruction. In the future, a specific method for BALKP detection should be performed for the exact BALKP level, and then we can know the source of ALKP that is increasing in metastasis cases [62].

We compared the clinical blood results between the no oral mass group and the tumor-bearing groups and found that the plasma ALB levels and the AGR in the two clinical groups, the oral mass group and the metastasis group, were significantly lower than in the no oral mass group. Conversely, the GLOB and the GAR in the clinical groups were significantly higher than in the normal group. In addition, the CAR was significantly higher in the clinical groups compared to the normal group. These results, like those in previous studies, showed that both hypoalbuminemia and the elevated CRP concentration were associated with malignancy and related to survival time [26]. In our study, NLR was also evaluated as biomarker of a systemic inflammatory response. Both clinical groups showed an increasing trend. This finding was related with the previous study in dogs with gingivitis and dogs with oropharyngeal tumors [63]. The predictive ability for patients with advanced cancers might be improved by combining CRP with other parameters, such as plasma albumin and NLR.

In human medicine, the oral microbiomes of diseases and healthy people are different from those of dogs according to the sequencing method [30]. Therefore, there seem to be considered differences between the oral microbiomes of the different species [31, 33, 47, 64, 65]. This study aimed to identify oral bacterial samples from dogs with no oral mass and clinical oral tumor-bearing dogs. We focused on the cultivable bacteria living in the oral mucosal surface of dogs. To this point, the assembly of the 16S rRNA gene of bacterial isolates provides a new view of the oral bacterial profile in the canine oral tumor-bearing group compared with the no oral mass group. Traditionally, bacterial identification in laboratories was performed using phenotypic tests, including Gram smear and biochemical tests, considering culture requirements and growth characteristics [66, 67]. However, these methods of bacterial identification have major limitations due to the phenotypic tests and biochemical tests. Nowadays, the modern PCR, automated DNA sequencing, and work on 16S rDNA sequencing bacteria can make an accurate identification of bacterial isolates. From this point, the accurate identification is one of the most important functions of clinical microbiology laboratories and solves the problem related to the traditional method [66, 68–70].

According to our results, the phylum Proteobacteria was the most abundant bacteria, as reported in previous studies [30, 33, 64]. In our study, Pasteurellaceae and Neisseriaceae were the two main bacterial families in the no oral mass group. The three majority genera of bacterial isolates were *Neisseria* spp., *Pasteurella* spp., and *Staphylococcus* spp. These three bacterial genera can usually be cultured from dog bite wounds in human [71, 72]. This point is related to the previous studies stating that *Neisseria* spp., including *N. canis*, *N. animaloris*, *N. dumasiana*, *N. zoodegmatis*, and *N. weaverii*, are one of the normal floras of dogs [71]. In addition, *Pasteurella* spp. and *Staphylococcus* spp. were also isolated as cultivable oral microbial in domestic dogs, but they were related to dental plaque samples in previous studies [29, 32]. In this study, Enterobacteriaceae was the majority bacterial family in both the clinical case groups. *Escherichia* spp. and *Shigella* spp. were not isolated from the dogs in the no oral mass group. Moreover, *Neisseria* spp. and *Pasteurella* spp. decreased in both oral mass groups. In addition, the bacteria in the genera *Aeromonas* spp., *Acinetobacter* spp., *Elizabethkingia* spp., *Klebsiella* spp., *Enterococcus* spp., *Corynebacterium* spp., and *Escherichia* spp. were found in the oral mass group. This alteration in oral bacterial isolation is may be due to the changes in the oral microenvironment that destroy the normal microbial population and conduct the opportunistic microorganisms to increase the population. The unique microbial profile in humans with SCC is different from the normal humans [36]. The discovery of bacterial replication in the tumor may be linked to the presence of bacterial nutrients and chemotactic compounds. Moreover, the alteration in the salivary microbiome of pancreatic cancer patients compared to the healthy control group empathized with those hypotheses. The researchers attempted to select the human oral microbiome as the noninvasive method for the diagnosis of the oral and gastrointestinal cancer. Recently, the researchers discovered the relationship of the dysbiosis of bacteria in gut and oral bacteria in dogs. In that study, the Bacteroides bacteria were shared in the intratumoral, oral, and gut bacterium community of canine mammary tumor cases. This result confirmed that these bacteria might travel from the gastrointestinal tract to the tumor sites [73]. This was related to previous study in human oral SCC and the bacterial translocation. They isolated the enteric bacteria such as *Klebsiella*, *Enterobacter*, and *Enterococcus* from the tissue of oral mass and regional lymph node before surgery and the infection of sites after operation [74]. In addition, the patients with malignant cancers are more susceptible to get infected by the pathogenic bacteria in comparison to benign patients [75]. Thus, based on our results, the alteration of the oral microbial profile and the bacterial translocation in the canine oral tumor-bearing group is an interesting point for further study related to the diagnosis and prediction of canine oral cancer.

Oh et al. reported that *Porphyromonas*, *Fusobacterium*, *Actinomyces*, *Neisseria* and *Pasteurella* were the most abundant genera of the bacterial swab from the buccal area and the supragingival plaque [30, 33]. According to the several distinct microbial habitats and the oral cavity area, the tongue is the most populated niche, and this area has an impact on other regions in the oral cavity. The tongue and saliva facilitated the bacteria to travel around the oral cavity [50]. Ruparella et al. and team reported that saliva exhibited the lowest bacterial diversity, the buccal and the tongue dorsum mucosa had the most similar bacteria [33]. Moreover, the teeth area and the dental plaque were the selected area for the periodontal disease [31, 32, 67, 70, 76–78]. So, the oral swab sample from the tongue's dorsum mucosa and the mucosa of the hard palate of each dog was taken to compare the microbes among the three groups of dogs in our study. This site of the sampling method showed the microbe profiles that differ from the previous study that interested in the buccal site and supragingival plaque. According to our study, the bacteria from the supragingival plaque that is related to the periodontitis might not interfere with the results of bacterial isolates in our samples. Many oral bacteria are slow growing, require complex culture media, and specific atmospheric requirements [69, 70, 79]. However, many bacterial species could not be cultured in this study because they are difficult to reproduce under laboratory conditions. Oral anaerobic bacteria such as *Porphyromonas* spp., *Fusobacterium* spp., *Bacteriodes* spp., *Capnocytophaga* spp., *Prevotella* spp., *Tannerella* spp., *Treponema* spp., and *Actinomyces* spp. reported in the previous study [29, 31, 48, 70, 77, 79, 80] were not found in our study. Some bacterial isolates have been detected from the environment, as the channel of a dog's mouth is exposed to the outdoor environment.

In veterinary medicine, there have been few reports about canine oral microbiomes with malignancies. The present study investigated oral microbial isolates in canines bearing oral tumor and compared the oral microbial isolates and blood clinical profiles among the no oral mass group and the clinical groups. Most frequently, isolates were normal oral flora which were not considered primary pathogens in the no oral mass group. The alterations in canine oral bacteria with oral mass compared to healthy dogs in this study may be related to microbiome alteration and dysbiosis. This suggested that bacteria may play the same potential roles in the pathogenesis and cancer progression of oral cancer as inflammatory pathogens in human. These were mentioned in the review article by Faden that there are many mechanisms including (a) chronic infection altered cell growth, (b) infection resulting in suppression of apoptosis, (c) chronic infection induced cell proliferation and DNA replication, (d) bacterial products caused the epithelial DNA damage and secondary hyper-proliferative epithelium, and (e) carcinogenic nitrosamine produced by *E. coli* linked to oral cancer development [51].

We focused on identifying cultivable bacteria living in the oral mucosal surface of dogs with the culture-based method and 16s rRNA gene sequencing. We obtained a lower number of bacterial colonies compared to recent reports using advance sequencing methods [30, 33]. However, our results did not interfere by the DNA component of dead bacteria that can be detected by the advance sequencing methods in the previous studies [34]. The most abundant bacterial phyla from the oral swab in this study were not similar as the study of McDonald et al. [81] and Dewhirst et al. [47]. Our data was similar to the study of Ruparell et al.[33]; this may be the result of the site of sample collection and the sample preparation for bacterial identification. The direct oral swab samples for next generation sequencing (NGS) or the whole genome sequencing (WGS) provided the bacterial taxonomies more than the culture based and 16s RNA gene sequencing method and those methods could detect the uncultured bacteria [62–64]. Furthermore, the different settings of the disease groups, sample collection between awakening dogs and those under anesthesia, could be a confounding factor leading to different results [82]. Limitations of this study were the small numbers of dogs in each group, the different tumor types within the tumor bearing-dog groups and the cultured bacterial isolate-based method. Due to the limitations of this study, we suggest that the number of samples should be increased to provide more interesting and valuable information for the further study.

## 5. Conclusion

This study provides the knowledge of the oral bacterial population from the culture-based with 16s rRNA gene sequencing method that points toward the role of bacterial alteration and tumor-promoting inflammation. Canine oral cancers have a poor prognosis and are related to the chronic status which may decrease the host oral mucosa immunity and increase the risk of secondary bacterial infection. We compared the bacterial isolates and blood profiles of dogs in the three groups. Significantly, both clinical groups showed anemia, an increase in NLR, GAR, CRP, and CAR, and a decrease in AGR compared to the normal group. *Neisseria* spp. was the main genus of bacterial isolates in the oral swabs. Interestingly, *Neisseria* spp. decreased in the clinical groups, and *Escherichia* spp. increased in the clinical groups. Our results on the differences of the oral bacteria among the groups of dogs and the increase in the CRP, CAR, and NLR suggest that the bacteria and the oral cancer may be related and play a potential role in cancer progression. Whether or not bacterial play the role in tumorigenesis and progression, it is an interesting point to further explore the effect that the bacteria may have on different phenotypes of cancer cells and their interactions with the cancer cells. Thus, further studies may provide the new knowledge in this field and/or facilitate the new treatment options. The studies should be conducted on the larger sample size and investigate the correlation between the specific bacteria, the type of canine oral mass, the clinical blood profiles, and the clinical stage of oral cancer.

## Figures and Tables

**Table 1:** The sex and age (mean ± standard deviation, SD) of dogs in this study.

Sex	No oral mass group		Oral mass group		Metastasis group	
	*n* (%)	Age	*n*(%)	Age	*n* (%)	Age
Male (*n* = 17)	9 (42.9)	9.11 ± 2.52	5 (62.5)	10.00 ± 3.94	3 (42.9)	9.00 ± 4.00
Female (*n* = 19)	12 (57.1)	9.00 ± 2.45	3 (37.5)	9.67 ± 5.13	4 (57.1)	13.75 ± 2.06

**Table 2:** Relative risk ratios (RRRs) of sex and age within the oral mass group and the metastasis group base outcome on the no oral mass group.

Parameters	Oral mass group			Metastasis group		
	RRR	95%CI	*P* value	RRR	95%CI	*P* value
Signalments						
Sex						
Male	1.0	(Reference category)		1.0	(Reference category)	
Female	0.42	0.08–2.29	0.313	0.75	0.12–4.82	0.762
Age	1.11	0.84–1.48	0.451	1.38	0.98–1.95	0.068

RRR were performed using multinomial logistic regression analysis and *P* values ≤0.05 were considered significant. CI: confidence interval.

**Table 3:** The histological diagnosis, signalments, the site of oral mass, TNM staging, and the clinical stage of dogs in the oral mass group.

Cases	Histopathological diagnosis	Breeds	Age (Y)	Sex	Sites	TNM staging	Clinical stages
1	Acanthomatous ameloblastoma	Beagle	9	M	Rostral mandible	T1N0M0	I
2	Acanthomatous ameloblastoma	Mixed	11	F	Rostral mandible	T1N0M0	I
3	Malignant melanotic melanoma	Shih Tzu	10	M	Right maxilla	T2N0M0	II
4	Squamous cell carcinoma (SCC)	Mixed	13	M	Rostral mandible	T2N0M0	II
5	Squamous cell carcinoma (SCC)	Mixed	14	M	Rostral mandible	T2N0M0	II
6	Fibrosarcoma (FSA)	Mixed	4	M	Rostral mandibular	T3N0M0	III
7	Malignant amelanotic melanoma	Yorkshire Terrier	14	F	Right maxilla	T3N0M0	III
8	Malignant amelanotic melanoma	Poodle	4	F	Right mandible	T3N0M0	III

Y = year; M = male; F = female; T = tumor; N = lymph node; M = metastasis.

**Table 4:** The histological diagnosis, signalments, the site of oral mass, TNM staging, and the clinical stage of dogs in the metastasis group.

Cases	Histopathological diagnosis	Breeds	Age (Y)	Sex	Sites	TNM staging	Clinical stage
1	Malignant melanotic melanoma	Golden Retriever	12	F	Right maxilla	T3N3M1	IV
2	Fibrosarcoma (FSA)	Poodle	12	F	Rostral maxilla	T3N3M1	IV
3	Chondrosarcoma	Mixed	5	M	Rostral maxilla	T3N3M1	IV
4	Fibrosarcoma (FSA)	Shih Tzu	13	M	Right maxilla	T3N3M1	IV
5	Malignant melanotic melanoma	Poodle	16	F	Right maxilla	T3N3M1	IV
6	Squamous cell carcinoma (SCC)	Shih Tzu	15	F	Left maxilla	T3N3M1	IV
7	Malignant melanotic melanoma	Thai Bangkaew	9	M	Rostral maxilla	T2N3M1	IV

Y = year; M = male; F = female; T = tumor; N = lymph node; M = metastasis.

**Table 5:** Comparison of the hematology and the blood chemistry profile of dogs in the no oral mass group, the oral mass group, and the metastasis group.

Parameters	Reference range	No oral mass (*n* = 21)	Oral mass (*n* = 8)	Metastasis (*n* = 6)	*P* value
Blood hematology					
RBC (×10^6^cells/*μ*L), median (IQR)	5.65–8.87	7.85 (7.57–8.12)^*∗*,*∗∗*^	6.17 (4.07–6.86)^*∗*^	5.68 (4.44–7.1)^*∗∗*^	0.0004
WBC (×10^3^cells/*μ*L), median (IQR)	5.05–16.76	9.14 (8.09–10.67)	9.64 (6.69–17.46)	13.00 (10.60–16.20)	0.0886
PLT (×10^3^cells/*μ*L), median (IQR)	148–484	181.00 (140.00–213.00)^*∗∗*^	273.50 (142.50–375.00)^*∗∗∗*^	482.00 (226.00–746.00)^*∗∗*,*∗∗∗*^	0.0335
NLR, median (IQR)		3.03 (2.32–3.61)^*∗∗*^	3.74 (3.08–6.50)	5.69 (4.53–6.20)^*∗∗*^	0.0032
Blood chemistry profile					
TP (g/dL), median (IQR)	5.2–8.2	6.80 (6.50–7.10)	6.90 (6.65–7.20)	7.35 (6.40–7.90)	0.4680
ALB (g/dL), mean ± SD	2.2–3.9	3.10 ± 0.19^*∗*,*∗∗*^	2.81 ± 0.23^*∗*,*∗∗∗*^	2.34 ± 0.25^*∗∗*,*∗∗∗*^	<0.0001
GLOB (g/dL), median (IQR)	2.5–4.5	3.60 (3.40–3.80)^*∗*,*∗∗*^	4.00 (3.80–4.35)^*∗*^	4.40 (4.10–5.60)^*∗∗*^	0.0020
AGR, mean ± SD		0.85 ± 0.07^*∗*,*∗∗*^	0.67 ± 0.11^*∗*,*∗∗∗*^	0.50 ± 0.13^*∗∗*,*∗∗∗*^	<0.0001
GAR, median (IQR)		1.15 (1.12–1.26)^*∗*,*∗∗*^	1.48 (1.37–1.54)^*∗*,*∗∗∗*^	1.78 (1.60–2.43)^*∗∗*,*∗∗∗*^	0.0001
CRP (mg/L), median (IQR)	<20	<10^*∗*,*∗∗*^	12.45 (10.00–23.40)^*∗*,*∗∗∗*^	112 (105.3–116.4)^*∗∗*,*∗∗∗*^	0.0001
CAR, median (IQR)		0.30 (0.29–0.33)^*∗*,*∗∗*^	4.51 (3.45–9.03)^*∗*,*∗∗∗*^	50.50 (47.00–51.00)^*∗∗*,*∗∗∗*^	0.0001
BUN (mg/dL), median (IQR)	7–27	17.00 (16.00–20.00)	18.00 (13.00–22.50)	14.00 (6.00–22.00)	0.6848
CRE (mg/dL), mean ± SD	0.5–1.8	0.97 ± 0.20	0.74 ± 0.28	0.91 ± 0.41	0.1267
ALT (U/L), median (IQR)	10–125	48.00 (16.00–74.00)	43.00 (24.50–53.00)	30.00 (25.00–44.00)	0.8017
ALKP (U/L), median (IQR)	23–212	36.50 (22.00–55.50)^*∗∗*^	48.50 (28.00–93.50)	131 (83.00–311.00)^*∗∗*^	0.0017

^*∗*^There is the difference between the no oral mass group and the oral mass group. ^*∗∗*^There is the difference between the no oral mass group and the metastasis group.^*∗∗∗*^There is the difference between the oral mass group and the metastasis group. RBC = red blood cell count; WBC = white blood cell count; PLT = platelet count; NLR = neutrophil to lymphocyte ratio; TP = total protein; ALB = albumin; GLOB = globulin; AGR = albumin to globulin ratio; GAR = globulin to albumin ratio; CRP = C-reactive protein; CAR = C-reactive protein to albumin ratio; BUN = blood urea nitrogen; ALT = alanine transferase; ALKP = alkaline phosphatase.

**Table 6:** Relative risk ratios (RRRs) of proportion of a dog in all groups compared within the level of RBC that had been low level and high level base outcome on the RBC normal level (5.65–8.87 × 10^6^cells/*μ*L).

Groups	RBC low level (less than 5.65 × 10^6^cells/*μ*L)			RBC high level (more than 8.87 × 10^6^cells/*μ*L)		
	RRR	95%CI	*P* value	RRR	95%CI	*P* value
No oral mass	1.0	(Reference category)		1.0	(Reference category)	
Oral mass	12.74	1.03–157.02	0.047	1.42	0.11–17.46	0.786
Metastasis	12.74	1.03–156.98	0.047	N/A	N/A	

RRR was performed using multinomial logistic regression analysis, *P* values ≤0.05 were considered significant, and N/A is not applicable.

**Table 7:** The bacterial identification with 16S rRNA gene taxonomy from the dog oral cavity.

16S rRNA gene taxonomy	Total	No mass	Oral mass	Metastasis
Phylum Bacteroidetes				
Class Flavobacteria				
Order Flavobacteriales				
Family Weeksellaceae				
Genus Elizabethkingia				
*Elizabethkingia anophelis*	1	—	1	—

II. Phylum Proteobacteria				
Class Alphaproteobacteria				
Order Rhodobacterales				
Family Rhodobacteraceae				
Genus Paracoccus				
*Paracoccus communis*	1	1	—	—
Class Betaproteobacteria				
Order Neisseriales				
Family Neisseriaceae				
Genus Neisseria				
*Neisseria animaloris*	1	—	—	1
*Neisseria canis*	1	1	—	—
*Neisseria dumasiana*	4	3	1	—
*Neisseria zoodegmatis*	10	9	1	—
Order Burkholderiales				
Family Alcaligenaceae				
Genus Achromobacter				
*Achromobacter insolitus*	1	—	1	—
Class Gammaproteobacteria				
Order Aeromonadales				
Family Aeromonadaceae				
Genus Aeromonas				
*Aeromonas hydrophila subsp. Hydrophila*	1	—	1	—
Order Enterobacterales				
Family Enterobacteriaceae				
Genus Escherichia				
*Escherichia coli*	6	—	2	4
Genus Klebsiella				
*Klebsiella quasipnneumoniae subsp. Similipneumoniae*	1	—	1	—
*Klebsiella pneumoniae*	1	—	1	—
Genus Shigella				
*Shigella flexneri*	1	—	—	1
Family Morganellaceae				
Genus Providencia				
*Providencia stuartii*	1	1	—	—
Order Pasteurellales				
Family Pasteurellaceae				
Genus Frederiksenia				
*Frederiksenia canicola*	5	5	—	—
Genus Pasteurella				
*Pasteurella canis*	4	3	—	1
*Pasteurella multocida*	7	6	1	—
Order Pseudomonadales				
Family Moraxellaceae				
Genus Acinetobacter				
*Acinetobacter seifertii*	1	—	1	—
Genus Moraxella				
*Moraxella* sp.	2	1	—	1
Family Pseudomonadaceae				
Genus Pseudomonas				
*Pseudomonas aeruginosa*	3	1	—	2

III. Phylum Firmicutes				
Class Bacilli				
Order Bacillales				
Family Staphylococcaceae				
Genus Staphylococcus				
*Staphylococcus pseudintermedius*	12	8	2	2
*Staphylococcus cohnii*	1	1	—	—
Family Streptococcaceae				
Genus Gemella				
*Gemella palaticanis*	1	1	—	—
Genus Streptococcus				
*Streptococcus* sp. (canine oral)	5	4	—	1
*Streptococcus dysgalactiae* subsp. *Equisimills*	1	—	1	—
Family Enterococcaceae				
Order Lactobacillales				
Genus Enterococcus				
*Enterococcus faecalis*	1	—	1	—
*Enterococcus raffinosus*	1	—	—	1

IV. Phylum Actinobacteria				
Class Actinobacteria				
Order Micrococcales				
Family Micrococcaceae				
Genus Rothia				
*Rothia nasimurium*	1	1	—	—
Order Mycobacteriales				
Family Corynebacteriaceae				
Genus Corynebacterium				
*Corynebacterium jeikeium*	1	—	—	1
*Corynebacterium mustelae*	1	—	1	—
Total	77	46	16	15

## Data Availability

The data used to support the findings of this study are available from the corresponding author upon request.

## Supplementary Materials

Supplementary Materials Supplementary file 1: the nucleotide sequences of bacterial isolates from the no oral mass dogs. Supplementary file 2: the nucleotide sequences of bacterial isolates from the dogs with oral mass.
