# Supplementary material for: Isolation of Oral Bacteria, Measurement of the C-Reactive Protein, and Blood Clinical Parameters in Dogs with Oral Tumor
Source: Vet Med Int. 2023 Mar 22;2023:2582774. doi: 10.1155/2023/2582774 (PMC10060073; doi:10.1155/2023/2582774)
Supplement: Supplementary Materials — Supplementary file 1: the nucleotide sequences of bacterial isolates from the no oral mass dogs. Supplementary file 2: the nucleotide sequences of bacterial isolates from the dogs with oral mass. [file 2582774.f1.zip › 1. Supplementary file 1-The nucleotide sequences of bacterial isolates from the no oral mass dogs.pdf]

The nucleotide sequences of bacterial isolates from the no oral mass dogs

Sequence	% Identical	Bacterial identification
CTCAGGATGAACGCTGGCGGCGTGCCTAATACATGCAAGTCGAGCGAACAGATAAGGAGCTTGCTCCTTTGACGTTAGCGGCGGACGGGT<br>GAGTAACACGTGGGTAACCTACCTATAAGACTGGAATAACTCCGGGAAACCGGGGCTAATGCCGGATAACATGTTGAACCGCATGGTTCT<br>ACAGTGAAAGACGGTCTTGCTGTCACTTATAGATGGACCCGCGCCGTATTAGCTAGTTGGTGGGGTAACGGCCTACCAAGGCGACGATAC<br>GTAGCCGACCTGAGAGGGTGATCGGCCACACTGGAACTGAGACACGGTCCAGACTCCTACGGGAGGCAGCAGTAGGGAATCTTCCGCAA<br>TGGGCGAAAGCCTGACGGAGCAACGCCGCGTGAGTGATGAAGGTCTTCGGATCGTAAAGCTCTGTTGTTAGGGAAGAACAAATGTGTAAG<br>TAACTGTGCACATCTTGACGGTACCTAACCAGAAAGCCACGGCTAACTACGTGCCAGCAGCCGCGGTAATACGTAGGTGGCAAGCGTTAT<br>CCGGAATTATTGGGCGTAAAGCGCGCGTAGGCGGTTTTTTAAGTCTGATGTGAAAGCCCACGGCTCAACCGTGGAGGGTCATTGGAAACT<br>GGAAAACTTGAGTGCAGAAGAGGAAAGTGGAATTCCATGTGTAGCGGTGAAATGCGCAGAGATATGGAGGAACACCAGTGGCGAAGGCG<br>GCTTTCTGGTCTGCAACTGACGCTGATGTGCGAAAGCGTGGGGATCAAACAGGATTAGATACCCTGGTAGTCCACGCCGTAAACGATGAG<br>TGCTAAGTGTTAGGGGGTTTCCGCCCCTTAGTGCTGCAGCTAACGCATTAAGCACTCCGCCTGGGGAGTACGGTCGCAAGACTGAAACTC<br>AAAGGAATTGACGGGGACCCGCACAAGCGGTGGAGCATGTGGTTTAATTCGAAGCAACGCGAAGAACCTTACCAAATCTTGACATCCTTT<br>GACCGCTCTAGAGATAGAGTTTTCCTCTTCGGAGGACAAAGTGACAGGTGGTGCATGGTTGTCGTCAGCTCGTGTCGTGAGATGTTGGGTT<br>AAGTCCCGCAACGAGCGCAACCCTTGAACTTAGTTGCCATCATTCAGTTGGGCACTCTAAGTTGACTGCCGGTGACAAACCGGAGGAAGG<br>TGGGGATGACGTCAAATCATCATGCCCCTTATGATTTGGGCTACACACGTGCTACAATGGACAATACAAAGGGCAGCGAAACCGCGAGGT<br>CAAGCAAATCCCATAAAGTTGTTCTCAGTTCGGATTGTAGTCTGCAACTCGACTACATGAAGCTGGAATCGCTAGTAATCGTAGATCAGCA<br>TGCTACGGTGAATACGTTCCCGGGTCTTGTACACACCGCCCGTCACACCACGAGAGTTTGTAACACCCGAAGCCGGTGGAGTAACCATTTT<br>GGAGCTAGCCGTCGAAGGTGGGACAAATGATTGGGGTGAAGTCTAC	100	*Staphylococcus pseudintermedius*
CTCAGATTGAACGCTGGCGGCAGGCTTAACACATGCAAGTCGAACGGTAGCAGGAAGAAAGCTTGCTTTTTTTGCTGACGAGTGGCGGAC<br>GGGTGAGTAATGCTTGGGAATCTGGCTTATGGAGGGGGATAACTGTGGGAAACTGCAGCTAATACCGCGTATAATCGAGAGATGAAAGGG<br>TGGGACCGTAAGGCCACTTGCCATAAGATGAGCCCAAGTGGGATTAGGTAGTTGGTGGGGTAAAGGCCTACCAAGCCTGCGATCTCTAGC<br>TGGTCTGAGAGGATGGCCAGCCACACTGGGACTGAGACACGGCCCAGACTCCTACGGGAGGCAGCAGTGGGGAATATTGCGCAATGGGG<br>GGAACCCTGACGCAGCCATGCCGCGTGAATGAAGAAGGCCTTCGGGTTGTAAAGTTCTTTCGGTGATGAGGAAGGGGTATTATTGAATAG<br>ATAATATCATTGACGTTAATTACAGAAGAAGCACCGGCTAACTCCGTGCCAGCAGCCGCGGTAATACGGAGGGTGCGAGCGTTAATCGGA<br>ATAACTGGGCGTAAAGGGCACGCAGGCGGACTTTTAAGTGAGATGTGAAATCCCCGAGCTTAACTTGGGAACTGCATTTCAGACTGGGAG<br>TCTAGAGTACTTTAGGGAGGGGTAGAATTCCACGTGTAGCGGTGAAATGCGTAGAGATGTGGAGGAATACCGAAGGCGAAGGCAGCCCCT<br>TGGGAATGTACTGACGCTCATGTGCGAAAGCGTGGGGAGCAAACAGGATTAGATACCCTGGTAGTCCACGCTGTAAACGCTGTCGATTTG<br>GGGATTGGACTTTATGTTTGGTGCCCGAAGCTAACGTGATAAATCGACCGCCTGGGGAGTACGGCCGCAAGGTTAAAACTCAAATGAATT<br>GACGGGGGCCCGCACAAGCGGTGGAGCATGTGGTTTAATTCGATGCAACGCGAAGAACCTTACCTACTCTTGACATCCTAAGAAGAGCTC<br>AGAGATGAGCTTGTGCCTTCGGGAACTTAGAGACAGGTGCTGCATGGCTGTCGTCAGCTCGTGTTGTGAAATGTTGGGTTAAGTCCCGCAA<br>CGAGCGCAACCCTTATCCTTTGTTGCCAGCGATTTGGTCGGGAACTCAAAGGAGACTGCCAGTGACAAACTGGAGGAAGGTGGGGATGAC<br>GTCAAGTCATCATGGCCCTTACGAGTAGGGCTACACACGTGCTACAATGGTGCATACAGAGGGCGGCGAGACGGCGACGTTGAGCGAATC<br>TCAGAAAGTGCATCTAAGTCCGGATTGGAGTCTGCAACTCGACTCCATGAAGTCGGAATCGCTAGTAATCGCAAATCAGAATGTTGCGGT<br>GAATACGTTCCCGGGCCTTGTACACACCGCCCGTCACACCATGGGAGTGGGTTGTACCAGAAGTGGATAGCTTAACGGAAACGAGGGCGT<br>TCACCACGGTATGATTCATGACTGGGGTG	99.8	*Pasteurella canis*
GGCTGCCTTTCGAGCTCAGCGTCAGTTACAGACCAGAGAGCCGCTTTCGCCACCGGTGTTCCTCCATATATCTACGCATTTCACCGCTACA<br>CATGGAATTCCACTCTCCCCTTCTGCACTCAAGTTCTCCAGTTTCCAAAGCATACATTGGTTGAGCCAATGCCTTTAACTTCAGACTTAAAG<br>AACCGCCTGCGCTCGCTTTACGCCCAATAAATCCGGACAACGCTCGGGACCTACGTATTACCGCGGCTGCTGGCACGTAGTTAGCCGTCC<br>CTTTCTGGTTAGTTACCGTCACATAATGGGTTTTCCACTCCCATTACCGTTCTTCTCTAACAACAGAGCTTTACGATCCGAAAACCTTCTTC<br>ACTCACGCGGCGTTGCTCGGTCAGGGTTCCCCCATTGCCGAAGATTCCCTACTGCTGCCTCCCGTAGGAGTCTGGGCCGTGTCTCAGTCC<br>CAGTGTGGCCGATCACCCTCTCAGGTCGGCTATGTATCGTCGCCTTGGTGAGCCTTTACCTCACCAACTAGCTAATACAACGCAGGTCCAT<br>CTCATAGTGGAGCAGTTGCCCCTTTTAAGTTCTTAACATGTGTTAAGCACTTTTATGCGGTATTAGCTATCGTTTCCAATAGTTATCCCCCG<br>CTATCAGGTAGGTTACCTACGCGTTACTCACCCGTTCGCAACTCCTTAGACTAGTGCAAGCACCTGTCCTCAGCGTTCTACTTGCATGTATT<br>AGGCACGCCGCCAGCGTTCGTCCTGAGCCATATTCCAAACTCTAAAAA	99.61	*Streptococcus canis*

CTCAGATTGAACGCTGGCGGCATGCTTTACACATGCAAGTCGAACGGCAGCACGGAAGAGCTTGCTCTTTTGGTGGCGAGTGGCGAACGG<br>GTGAGTAACGCATCGGAACGTACCGAGTAGTGGGGGATAACTGTCCGAAAGGATGGCTAATACCGCATATTCTCTGAGGAGGAAAGCAGG<br>GGACCTTCGGGCCTTGCGCTATTTGAGCGGCCGATGTCTGATTAGCTAGTTGGTGGGGTAAAGGCCTACCAAGGCGACGATCAGTAGCGG<br>GTCTGAGAGGATGATCCGCCACACTGGGACTGAGACACGGCCCAGACTCCTACGGGAGGCAGCAGTGGGGAATTTTGGACAATGGGGG<br>AACCCTGATCCAGCCATGCCGCGTGTCTGAAGAAGGCCTTCGGGTTGTAAAGGACTTTTGTCAGGGAAGAAAAGCTTGAGGTTAATACCC<br>TTGAGTGATGACGGTACCTGAAGAATAAGCACCGGCTAACTACGTGCCAGCAGCCGCGGTAATACGTAGGGTGCGAGCGTTAATCGGAAT<br>TACTGGGCGTAAAGCGGGCGCAGACGGTTACTTAAGTCAGATGTGAAATCCCCGGGCTCAACCTGGGAACTGCGTTTGAAACTGGGTGAC<br>TAGAGTATGTCAGAGGGGGGTAGAATTCCACGTGTAGCAGTGAAATGCGTAGAGATGTGGAGGAATACCGATGGCGAAGGCAGCCCCCT<br>GGGATAATACTGACGTTCATGCCCGAAAGCGTGGGTAGCAAACAGGATTAGATACCCTGGTAGTCCACGCCCTAAACGATGTCAATTAGC<br>TGTTGGGGCACTTGATGCCTTAGTAGCGTAGCTAACGCGTGAAATTGACCGCCTGGGGAGTACGGTCGCAAGATTAAAACTCAAAGGAAT<br>TGACGGGGACCCGCACAAGCGGTGGATGATGTGGATTAATTCGATGCAACGCGAAGAACCTTACCTGGTCTTGACATGTACGGAATCCTC<br>CAGAGACGGAGGAGTGCCTTCGGGAGCCGTAACACAGGTGCTGCATGGCTGTCGTCAGCTCGTGTCGTGAGATGTTGGGTTAAGTCCCGC<br>AACGAGCGCAACCCTTGTCATTAGTTGCCATCATTAAGTTGGGCACTCTAATGAGACTGCCGGTGACAAGCCGGAGGAAGGTGGGGATGA<br>CGTCAAGTCCTCATGGCCCTTATGACCAGGGCTTCACACGTCATACAATGGTCGGTACAGAGGGTAGCCAAGCCGCGAGGTGGAGCCAAT<br>CCCAGAAAACCGATCGTAGTCCGGATTGCACTCTGCAACTCGAGTGCATGAAGTCGGAATCGCTAGTAATCGCAGGTCAGCATACTGCGG<br>TGAATACGTTCCCGGGTCTTGTACACACCGCCCGTCACACCATGGGAGTGGGGGATACCAGAAGTAGGTAGGGTAACCGTAAGGAGCCCG<br>CTTACCACGGTATGCTTCATGACTGGGG	100	*Neisseria canis*
ACTTTCCTTTTCGGGCATGACGTCAGTATTATCCCAGGGGGCTGCCTTCGCCATCGGTATTCCTCCACATCTCTACGCATTTCACTGCTACA<br>CGTGGAATTCTACCCCCCTCTGACATACTCTAGCCACCCAGTTTCAAACGCAGTTCCCAGGTTGAGCCCGGGGATTTCACATCTGACTTAA<br>GTAACCGTCTGCGCCCGCTTTACGCCCAGTAATTCCGATTAACGCTCGCACCCTACGTATTACCGCGGCTGCTGGCACGTAGTTAGCCGGT<br>GCTTATTCTTCAGGTACCGTCATAACTCAAGGGTATTAGCCTCAAGCTTTTCTTCCCTGACAAAAGTCCTTTACAACCCGAAGGCCTTCTTC<br>AGACACGCGGCATGGCTGGATCAGGCTTGCGCCCATTGTCCAAAATTCCCCACTGCTGCCTCCCGTAGGAGTCTGGGCCGTGTCTCAGTCC<br>CAGTGTGGCGGATCATCCTCTCAGACCCGCTACTGATCGTCGCCTTGGTAGGCCTTTACCCCACCAACCAGCTAATCAGACATCGGCCGCT<br>CGAATAGCGCGAGGTCCAAAGATCCCCCGCTTTCCTTCTCAAAGCGTATGCGGTATTAGCCATCCTTTCGGACAGTTATCCCCCACTACTC<br>GGTACGTTCCGATGCGTTACTCACCCGTTCGCCACTCGCCACCCAAGAAGCAAGCTTCTTTGTGCTGCCGTCCGACTTGCATGTGTAAAGC<br>ATGCCGCCAGCGTTCAATCTGAGCCATATACCCAAACTCCTAAAAA	99.2	*Neisseria dumasiana*
CTCAGATTGAACGCTGGCGGCAGGCTTAACACATGCAAGTCGAACGGTAGCAGGAGAAAGCTTGCTTTCTTGCTGACGAGTGGCGGACGG<br>GTGAGTAATGCTTGGGAATCTGGCTTATGGAGGGGGATAACTACTGGAAACGGTAGCTAATACCGCGTAATATCGAGAGATTAAAGACTG<br>GGACCTACGGGCCAGTTGCCATAAGATGAGCCCAAGTGGGATTAGGTAGTTGGTGGGGTAAAGGCCTACCAAGCCCGCGATCTCTAGCTG<br>GTCTGAGAGGATGACCAGCCACACTGGAACTGAGACACGGTCCAGACTCCTACGGGAGGCAGCAGTGGGGAATATTGCACAATGGGGG<br>AACCCTGATGCAGCCATGCCGCGTGAATGAAGAAGGCCTTCGGGTTGTAAAGTTCTTTCGGTGGTGAGGAAGGTATCAACTTTAATAGAG<br>TTGGTAATTGACGTTATCCACAGAAGAAGCACCGGCTAACTCCGTGCCAGCAGCCGCGGTAATACGGAGGGTGCGAGCGTTAATCGGAAT<br>GACTGGGCGTAAAGGGCACGCAGGCGGATGCTTAAGTGAGATGTGAAAGCCCCGGGCTTAACCTGGGAATTGCATTTCAGACTGGGCATC<br>TAGAGTATTTTAGGGAGGGGTAGAATTCCACGTGTAGCGGTGAAATGCGTAGAGATGTGGAGGAATACCGAAGGCGAAGGCAGCCCCTTG<br>GGAATATACTGACGCTCATGTGCGAAAGCGTGGGGAGCAAACAGGATTAGATACCCTGGTAGTCCACGCTGTAAACGATGTCGATTTGGG<br>GATTGGGGTTTAACTCTGGTGCCCGAAGCTAACGTGATAAATCGACCGCCTGGGGAGTACGGCCGCAAGGTTAAAACTCAAATGAATTGA<br>CGGGGGCCCGCACAAGCGGTGGAGCATGTGGTTTAATTCGATGCAACGCGAAGAACCTTACCTACTCTTGACATCCATGGAAGTTCGCAG<br>AGATGTGAATGTGCCTTCGGGAACCATGAGACAGGTGCTGCATGGCTGTCGTCAGCTCGTGTTGTGAAATGTTGGGTTAAGTCCCGCAACG<br>AGCGCAACCCTTATCCTTTGTTGCCAGCGATTAGGTCGGGAACTCAAAGGAGACTGCCAGTGATAAACTGGAGGAAGGTGGGGATGACGT<br>CAAGTCATCATGGCCCTTACGAGTAGGGCTACACACGTGCTACAATGGTGCATACAGAGGGAAGCAATATGGCGACATGGAGCAAATCTC<br>ACAAAGTGCATCTAAGTCCGGATTGGAGTCTGCAACTCGACTCCATGAAGTCGGAATCGCTAGTAATCGCAAATCAGAATGTTGCGGTGA<br>ATACGTTCCCGGGCCTTGTACACACCGCCCGTCACACCATGGGAGTGGGTTGTACCAGAAGTAGATAGCTTAACCGCAAGGGGGGCGTTT<br>ACCACGGTATGATTCATGACTGGGGTG	99.52	*Frederiksenia canicola*

CTCAGGATGAACGCTGGCGGCGTGCCTAATACATGCAAGTCGAGCGAACAGATAAGGAGCTTGCTCCTTTGACGTTAGCGGCGGACGGGT<br>GAGTAACACGTGGGTAACCTACCTATAAGACTGGAATAACTCCGGGAAACCGGGGCTAATGCCGGATAACATGTTGAACCGCATGGTTCT<br>ACAGTGAAAGACGGTCTTGCTGTCACTTATAGATGGACCCGCGCCGTATTAGCTAGTTGGTGGGGTAACGGCCTACCAAGGCGACGATAC<br>GTAGCCGACCTGAGAGGGTGATCGGCCACACTGGAACTGAGACACGGTCCAGACTCCTACGGGAGGCAGCAGTAGGGAATCTTCCGCAA<br>TGGGCGAAAGCCTGACGGAGCAACGCCGCGTGAGTGATGAAGGTCTTCGGATCGTAAAGCTCTGTTGTTAGGGAAGAACAAATGTGTAAG<br>TAACTGTGCACATCTTGACGGTACCTAACCAGAAAGCCACGGCTAACTACGTGCCAGCAGCCGCGGTAATACGTAGGTGGCAAGCGTTAT<br>CCGGAATTATTGGGCGTAAAGCGCGCGTAGGCGGTTTTTTAAGTCTGATGTGAAAGCCCACGGCTCAACCGTGGAGGGTCATTGGAAACT<br>GGAAAACTTGAGTGCAGAAGAGGAAAGTGGAATTCCATGTGTAGCGGTGAAATGCGCAGAGATATGGAGGAACACCAGTGGCGAAGGCG<br>GCTTTCTGGTCTGCAACTGACGCTGATGTGCGAAAGCGTGGGGATCAAACAGGATTAGATACCCTGGTAGTCCACGCCGTAAACGATGAG<br>TGCTAAGTGTTAGGGGGTTTCCGCCCCTTAGTGCTGCAGCTAACGCATTAAGCACTCCGCCTGGGGAGTACGGTCGCAAGACTGAAACTC<br>AAAGGAATTGACGGGGACCCGCACAAGCGGTGGAGCATGTGGTTTAATTCGAAGCAACGCGAAGAACCTTACCAAATCTTGACATCCTTT<br>GACCGCTCTAGAGATAGAGTTTTCCTCTTCGGAGGACAAAGTGACAGGTGGTGCATGGTTGTCGTCAGCTCGTGTCGTGAGATGTTGGGTT<br>AAGTCCCGCAACGAGCGCAACCCTTGAACTTAGTTGCCATCATTCAGTTGGGCACTCTAAGTTGACTGCCGGTGACAAACCGGAGGAAGG<br>TGGGGATGACGTCAAATCATCATGCCCCTTATGATTTGGGCTACACACGTGCTACAATGGACAATACAAAGGGCAGCGAAACCGCGAGGT<br>CAAGCAAATCCCATAAAGTTGTTCTCAGTTCGGATTGTAGTCTGCAACTCGACTACATGAAGCTGGAATCGCTAGTAATCGTAGATCAGCA<br>TGCTACGGTGAATACGTTCCCGGGTCTTGTACACACCGCCCGTCACACCACGAGAGTTTGTAACACCCGAAGCCGGTGGAGTAACCATTTT<br>GGAGCTAGCCGTCGAAGGTGGGACAAATGATTGGGGT	100	*Staphylococcus pseudintermedius*
AGGCTTCTTTCGCATGAGCGTCAGTATATTCCCAAGGGGCTGCCTTCGCCTTCGGTATTCCTCCACATCTCTACGCATTTCACCGCTACACG<br>TGGAATTCTACCCCTCCCTAAAATACTCTAGATGCCCAGTCTGAAATGCAATTCCCAGGTTAAGCCCGGGGCTTTCACATCTCACTTAAGC<br>ATCCGCCTGCGTGCCCTTTACGCCCAGTCATTCCGATTAACGCTCGCACCCTCCGTATTACCGCGGCTGCTGGCACGGAGTTAGCCGGTGC<br>TTCTTCTGTGGATAACGTCAATTACCAACTCTATTAAAGTTGATACCTTCCTCACCACCGAAAGAACTTTACAACCCGAAGGCCTTCTTCAT<br>TCACGCGGCATGGCTGCATCAGGGTTCCCCCATTGTGCAATATTCCCCACTGCTGCCTCCCGTAGGAGTCTGGACCGTGTCTCAGTTCCA<br>GTGTGGCTGGTCATCCTCTCAGACCAGCTAGAGATCGCGGGCTTGGTAGGCCTTTACCCCACCAACTACCTAATCCCACTTGGGCTCATCT<br>TATGGCAACTGGCCCGTAGGTCCCAGTCTTTAATCTCTCGATATTACGCGGTATTAGCTACCGTTTCCAGTAGTTATCCCCCTCCATAAGCC<br>AGATTCCCAAGCATTACTCACCCGTCCGCCACTCGTCAGCAAGAAAGCAAGCTTTCTCCTGCTACCGTTCGACTTGCATGTGTTAAGCCTG<br>CCGCCAGCGTTCAATCTGAGCCATGATTCCAAACTCTAA	98.69	*Frederiksenia canicola*
AGATTGAACGCTGGCGGCATGCTTTACACATGCAAGTCGGACGGCAGCACAGAGAAGCTTGCTTCTTGGGTGGCGAGTGGCGAACGGGTG<br>AGTAACGCATCGGAACGTACCGAGTAGTGGGGGATAACTGTCCGAAAGGATGGCTAATACCGCATACGCTTTGAGAAGGAAAGCGGGGG<br>ATCTTCGGACCTCGCGCTATTCGAGCGGCCGATGTCTGATTAGCTAGTTGGTGGGGTAAAGGCCTACCAAGGCGACGATCAGTAGCGGGT<br>CTGAGAGGATGATCCGCCACACTGGGACTGAGACACGGCCCAGACTCCTACGGGAGGCAGCAGTGGGGAATTTTGGACAATGGGCGCAA<br>GCCTGATCCAGCCATGCCGCGTGTCTGAAGAAGGCCTTCGGGTTGTAAAGGACTTTTGTCAGGGAAGAAAAGCTTGAGGCTAATACCCTT<br>AAGTGATGACGGTACCTGAAGAATAAGCACCGGCTAACTACGTGCCAGCAGCCGCGGTAATACGTAGGGTGCGAGCGTTAATCGGAATTA<br>CTGGGCGTAAAGCGGGCGCAGACGGTTACTTAAGTCAGATGTGAAATCCCCGGGCTCAACCTGGGAACTGCGTTTGAAACTGGGTAGCTA<br>GAGTATGTCAGAGGGGGGTAGAATTCCACGTGTAGCAGTGAAATGCGTAGAGATGTGGAGGAATACCGATGGCGAAGGCAGCCCCCTGG<br>GATAATACTGACGTTCATGCCCGAAAGCGTGGGTAGCAAACAGGATTAGATACCCTGGTAGTCCACGCCCTAAACGATGTCAATTAGCTG<br>TTGGGGCACTTGATGCCTTAGTAGCGTAGCTAACGCGTGAAATTGACCGCCTGGGGAGTACGGTCGCAAGATTAAAACTCAAAGGAATTG<br>ACGGGGACCCGCACAAGCGGTGGATGATGTGGATTAATTCGATGCAACGCGAAGAACCTTACCTGGTCTTGACATGTACGGAATCCTCCA<br>GAGACGGAGGAGTGCCTTCGGGAGCCGTAACACAGGTGCTGCATGGCTGTCGTCAGCTCGTGTCGTGAGATGTTGGGTTAAGTCCCGCAA<br>CGAGCGCAACCCTTGTCATTAGTTGCCATCATTAAGTTGGGCACTCTAATGAGACTGCCGGTGACAAGCCGGAGGAAGGTGGGGATGACG<br>TCAAGTCCTCATGGCCCTTATGACCAGGGCTTCACACGTCATACAATGGTCGGTACAGAGGGTAGCCAAGCCGCGAGGTGGAGCCAATCC<br>CACAAAACCGATCGTAGTCCGGATTGCACTCTGCAACTCGAGTGCATGAAGTCGGAATCGCTAGTAATCGCAGGTCAGCATACTGCGGTG<br>AATACGTTCCCGGGTCTTGTACACACCGCCCGTCACACCATGGGAGTGGGGGATACCAGAAGTAGGTAGGGTAACCGCAAGGAGCCCGCT<br>TACCACGGTATGCTTCATGACTGGGGTGAAGTCGTA	99.93	*Neisseria dumasiana*

TGCTTCTTTTCGCATCAGCGTCAGTTGCAGACCAGAAAGCCGCCTTCGCCACTGGTGTTCCTCCATATCTCTGCGCATTTCACCGCTACACA<br>TGGAATTCCACTTTCCTCTTCTGCACTCAAGTTTTCCAGTTTCCAATGACCCTCCACGGTTGAGCCGTGGGCTTTCACATCAGACTTAAAAA<br>ACCGCCTACGCGCGCTTTACGCCCAATAATTCCGGATAACGCTTGCCACCTACGTATTACCGCGGCTGCTGGCACGTAGTTAGCCGTGGCT<br>TTCTGGTTAGGTACCGTCAAGATGTGCACAGTTACTTACACATTTGTTCTTCCCTAACAACAGAGCTTTACGATCCGAAGACCTTCATCACT<br>CACGCGGCGTTGCTCCGTCAGGCTTTCGCCCATTGCGGAAGATTCCCTACTGCTGCCTCCCGTAGGAGTCTGGACCGTGTCTCAGTTCCAG<br>TGTGGCCGATCACCCTCTCAGGTCGGCTACGTATCGTCGCCTTGGTAGGCCGTTACCCCACCAACTAGCTAATACGGCGCGGGTCCATCTA<br>TAAGTGACAGCAAGACCGTCTTTCACTGTAGAACCATGCGGTTCAACATGTTATCCGGCATTAGCCCCGGTTTCCCGGAGTTATTCCAGTC<br>TTATAGGTAGGTTACCCACGTGTTACTCACCCGTCCGCCGCTAACGTCAAAGGAGCAAGCTCCTTATCTGTTCGCTCGACTTGCATGTATT<br>AGGCACGCCGCCAGCGTTCATCCTGAGCCATATATCCAAACTCTAAAAA	99.48	*Staphylococcus pseudintermedius*
AGATTGAACGCTGGCGGCATGCTTTACACATGCAAGTCGGACGGCAGCACAGAGAAGCTTGCTTCTTGGGTGGCGAGTGGCGAACGGGTG<br>AGTAATGCATCGGAACGTACCGAGTAGTGGGGGATAACTGTCCGAAAGGATGGCTAATACCGCATACGCTTTGAGAAGGAAAGCAGGGG<br>CTCTTCGGACCTTGCGCTATTCGAGCGGCCGATGTCTGATTAGCTGGTTGGTGGGGTAAAGGCCTACCAAGGCGACGATCAGTAGCGGGT<br>CTGAGAGGATGATCCGCCACACTGGGACTGAGACACGGCCCAGACTCCTACGGGAGGCAGCAGTGGGGAATTTTGGACAATGGGCGCAA<br>GCCTGATCCAGCCATGCCGCGTGTCTGAAGAAGGCCTTCGGGTTGTAAAGGACTTTTGTCAGGGAAGAAAAGGTTGTGGTTAATACCCAT<br>GACTGATGACGGTACCTGAAGAATAAGCACCGGCTAACTACGTGCCAGCAGCCGCGGTAATACGTAGGGTGCGAGCGTTAATCGGAATTA<br>CTGGGCGTAAAGCGGGCGCAGACGGTTACTTAAGTCAGATGTGAAATCCCCGGGCTCAACCTGGGAACTGCGTTTGAAACTGGGTGACTA<br>GAGTATGTCAGAGGGGGGTAGAATTCCACGTGTAGCAGTGAAATGCGTAGAGATGTGGAGGAATACCGATGGCGAAGGCAGCCCCCTGG<br>GATAATACTGACGTTCATGCCCGAAAGCGTGGGTAGCAAACAGGATTAGATACCCTGGTAGTCCACGCCCTAAACGATGTCAATTAGCTG<br>TTGGGCAACTTGATTGCTTAGTAGCGTAGCTAACGCGTGAAATTGACCGCCTGGGGAGTACGGTCGCAAGATTAAAACTCAAAGGAATTG<br>ACGGGGACCCGCACAAGCGGTGGATGATGTGGATTAATTCGATGCAACGCGAAGAACCTTACCTGGTCTTGACATGTACGGAATCCTCCA<br>GAGACGGAGGAGTGCCTTCGGGAGCCGTAACACAGGTGCTGCATGGCTGTCGTCAGCTCGTGTCGTGAGATGTTGGGTTAAGTCCCGCAA<br>CGAGCGCAACCCTTGTCATTAGTTGCCATCATTTGGTTGGGCACTCTAATGAGACTGCCGGTGACAAGCCGGAGGAAGGTGGGGATGACG<br>TCAAGTCCTCATGGCCCTTATGACCAGGGCTTCACACGTCATACAATGGTCGGTACAGAGGGTAGCCAAGCCGCGAGGTGGAGCCAATCC<br>CACAAAACCGATCGTAGTCCGGATTGCACTCTGCAACTCGAGTGCATGAAGTCGGAATCGCTAGTAATCGCAGGTCAGCATACTGCGGTG<br>AATACGTTCCCGGGTCTTGTACACACCGCCCGTCACACCATGGGAGTGGGGGATACCAGAAGTAGGTAGGGTAACCGCAAGGAGCCCGCT<br>TACCACGGTATGCTTCATGACTGGGGTG	100	*Neisseria zoodegmatis*
AGTCTTCCTTTCGCAATGAGCGTCAGTATATTCCCAAGGGGCTGCCTTCGCCTTCGGTATTCCTCCACATCTCTACGCATTTCACCGCTACA<br>CGTGGAATTCTACCCCTCCCTAAAATACTCTAGATGCCCAGTCTGAAATGCAATTCCCAGGTTAAGCCCGGGGCTTTCACATCTCACTTAA<br>GCATCCGCCTGCGTGCCCTTTACGCCCAGTCATTCCGATTAACGCTCGCACCCTCCGTATTACCGCGGCTGCTGGCACGGAGTTAGCCGGT<br>GCTTCTTCTGTGGATAACGTCAATTACCAACTCTATTAAAGTTGATACCTTCCTCACCACCGAAAGAACTTTACAACCCGAAGGCCTTCTT<br>CATTCACGCGGCATGGCTGCATCAGGGTTCCCCCATTGTGCAATATTCCCCACTGGTGGCCTCCCGAAGGAGCTGGGACCGGGTCTCAGT<br>TCCAGTGTGGCTGGTCATCCTCTCAGACCAGCTAGAGATCGCGGGCTTGGTAGGCCTTTACCCCACCAACTACCTAATCCCACTTGGGCTC<br>ATCTTATGGCAACTGGCCCGTAGGTCCCAGTCTTTAATCTCTCGATATTACGCGGTATTAGCTACCGTTTCCAGTAGTTATCCCCCTCCATA<br>AGCCAGATTCCCAAGCATTACTCACCCGTCCGCCACTCGTCAGCAAGAAAGCAAGCTTTCTCCTGCTACCGTTCGACTTGCATGTGTTAAG<br>CCTGCCGCCAGCGTTCAATCTGAGCCATGATCCAAACTCTAAAAA	99	*Frederiksenia canicola*

CTCAGATTGAACGCTGGCGGCAGGCTTAACACATGCAAGTCGAACGGTAGCAGGGAGAAAGCTTGCTTTCTTTGCTGACGAGTGGCGGAC<br>GGGTGAGTAATGCTTGGGAATCTGGCTTATGGAGGGGGATAACTGCGGGAAACTGCAGCTAATACCGCGTATTATCGGAAGATGAAAGGG<br>TGGGACCTTCGGGCCACCTGCCATAAGATGAGCCCAAGTGGGATTAGGTAGTTGGTGGGGTAAGGGCCTACCAAGCCGACGATCTCTAGC<br>TGGTCTGAGAGGATGACCAGCCACACTGGGACTGAGACACGGCCCAGACTCCTACGGGAGGCAGCAGTGGGGAATATTGCGCAATGGGG<br>GGAACCCTGACGCAGCCATGCCGCGTGAATGAAGAAGGCCTTCGGGTTGTAAAGTTCTTTCGGTAATGAGGAAGGGGTATTATTGAATAG<br>ATAATATCATTGACGTTAATTACAGAAGAAGCACCGGCTAACTCCGTGCCAGCAGCCGCGGTAATACGGAGGGTGCGAGCGTTAATCGGA<br>ATAACTGGGCGTAAAGGGCACGCAGGCGGACTTTTAAGTGAGATGTGAAATCCCCGAGCTTAACTTGGGAACTGCATTTCAGACTGGGAG<br>TCTAGAGTACTTTAGGGAGGGGTAGAATTCCACGTGTAGCGGTGAAATGCGTAGAGATGTGGAGGAATACCGAAGGCGAAGGCAGCCCCT<br>TGGGAATGTACTGACGCTCATGTGCGAAAGCGTGGGGAGCAAACAGGATTAGATACCCTGGTAGTCCACGCTGTAAACGCTGTCGATTTG<br>GGGATTGGGCTTTATGCTTGGTGCCCGAAGCTAACGTGATAAATCGACCGCCTGGGGAGTACGGCCGCAAGGTTAAAACTCAAATGAATT<br>GACGGGGGCCCGCACAAGCGGTGGAGCATGTGGTTTAATTCGATGCAACGCGAAGAACCTTACCTACTCTTGACATCCTAAGAAGAGCTC<br>AGAGATGAGCTTGTGCCTTCGGGAACTTAGAGACAGGTGCTGCATGGCTGTCGTCAGCTCGTGTTGTGAAATGTTGGGTTAAGTCCCGCAA<br>CGAGCGCAACCCTTATCCTTTGTTGCCAGCGATTTGGTCGGGAACTCAAAGGAGACTGCCAGTGACAAACTGGAGGAAGGTGGGGATGAC<br>GTCAAGTCATCATGGCCCTTACGAGTAGGGCTACACACGTGCTACAATGGTGCATACAGAGGGCGGCGAGACGGCGACGTTGAGCGAATC<br>TCATAAAGTGCATCTAAGTCCGGATTGGAGTCTGCAACTCGACTCCATGAAGTCGGAATCGCTAGTAATCGCAAATCAGAATGTTGCGGT<br>GAATACGTTCCCGGGCCTTGTACACACCGCCCGTCACACCATGGGAGTGGGTTGTACCAGAAGTGGATAGCTTAACGGAAACGAGGGCGT<br>TCACCACGGTATGATTCATGACTGGGGTG	99.86	*Pasteurella canis*
CTCAGGATGAACGCTGGCGGCGTGCCTAATACATGCAAGTCGAGCGAACAGATAAGGAGCTTGCTCCTTTGACGTTAGCGGCGGACGGGT<br>GAGTAACACGTGGGTAACCTACCTATAAGACTGGAATAACTCCGGGAAACCGGGGCTAATGCCGGATAACATGTTGAACCGCATGGTTCT<br>ACAGTGAAAGACGGTCTTGCTGTCACTTATAGATGGACCCGCGCCGTATTAGCTAGTTGGTGGGGTAACGGCCTACCAAGGCGACGATAC<br>GTAGCCGACCTGAGAGGGTGATCGGCCACACTGGAACTGAGACACGGTCCAGACTCCTACGGGAGGCAGCAGTAGGGAATCTTCCGCAA<br>TGGGCGAAAGCCTGACGGAGCAACGCCGCGTGAGTGATGAAGGTCTTCGGATCGTAAAGCTCTGTTGTTAGGGAAGAATAAATGTGTAAG<br>TAACTGTGCACATCTTGACGGTACCTAACCAGAAAGCCACGGCTAACTACGTGCCAGCAGCCGCGGTAATACGTAGGTGGCAAGCGTTAT<br>CCGGAATTATTGGGCGTAAAGCGCGCGTAGGCGGTTTTTTAAGTCTGATGTGAAAGCCCACGGCTCAACCGTGGAGGGTCATTGGAAACT<br>GGAAAACTTGAGTGCAGAAGAGGAAAGTGGAATTCCATGTGTAGCGGTGAAATGCGCAGAGATATGGAGGAACACCAGTGGCGAAGGCG<br>GCTTTCTGGTCTGCAACTGACGCTGATGTGCGAAAGCGTGGGGATCAAACAGGATTAGATACCCTGGTAGTCCACGCCGTAAACGATGAG<br>TGCTAAGTGTTAGGGGGTTTCCGCCCCTTAGTGCTGCAGCTAACGCATTAAGCACTCCGCCTGGGGAGTACGGTCGCAAGACTGAAACTC<br>AAAGGAATTGACGGGGACCCGCACAAGCGGTGGAGCATGTGGTTTAATTCGAAGCAACGCGAAGAACCTTACCAAATCTTGACATCCTTT<br>GACCGCTCTAGAGATAGAGTTTTCCTCTTCGGAGGACAAAGTGACAGGTGGTGCATGGTTGTCGTCAGCTCGTGTCGTGAGATGTTGGGTT<br>AAGTCCCGCAACGAGCGCAACCCTTGAACTTAGTTGCCATCATTCAGTTGGGCACTCTAAGTTGACTGCCGGTGACAAACCGGAGGAAGG<br>TGGGGATGACGTCAAATCATCATGCCCCTTATGATTTGGGCTACACACGTGCTACAATGGACAATACAAAGGGCAGCGAAACCGCGAGGT<br>CAAGCAAATCCCATAAAGTTGTTCTCAGTTCGGATTGTAGTCTGCAACTCGACTACATGAAGCTGGAATCGCTAGTAATCGTAGATCAGCA<br>TGCTACGGTGAATACGTTCCCGGGTCTTGTACACACCGCCCGTCACACCACGAGAGTTTGTAACACCCGAAGCCGGTGGAGTAACCATTTT<br>GGAGCTAGCCGTCGAAGGTGGGACAAATGATTGGGGTGAAGTCGTACAGGGG	100	*Staphylococcus pseudintermedius*

CTCAGATTGAACGCTGGCGGCAGGCTTAACACATGCAAGTCGAACGGTAGCAGGAAGAAAGCTTGCTTTTTTTGCTGACGAGTGGCGGAC<br>GGGTGAGTAATGCTTGGGAATCTGGCTTATGGAGGGGGATAACTGTGGGAAACTGCAGCTAATACCGCGTATAATCGAGAGATGAAAGGG<br>TGGGACCGTAAGGCCACTTGCCATAAGATGAGCCCAAGTGGGATTAGGTAGTTGGTGGGGTAAAGGCCTACCAAGCCTGCGATCTCTAGC<br>TGGTCTGAGAGGATGGCCAGCCACACTGGGACTGAGACACGGCCCAGACTCCTACGGGAGGCAGCAGTGGGGAATATTGCGCAATGGGG<br>GGAACCCTGACGCAGCCATGCCGCGTGAATGAAGAAGGCCTTCGGGTTGTAAAGTTCTTTCGGTGATGAGGAAGGGGTATTATTGAATAG<br>ATAATATCATTGACGTTAATTACAGAAGAAGCACCGGCTAACTCCGTGCCAGCAGCCGCGGTAATACGGAGGGTGCGAGCGTTAATCGGA<br>ATAACTGGGCGTAAAGGGCACGCAGGCGGACTTTTAAGTGAGATGTGAAATCCCCGAGCTTAACTTGGGAACTGCATTTCAGACTGGGAG<br>TCTAGAGTACTTTAGGGAGGGGTAGAATTCCACGTGTAGCGGTGAAATGCGTAGAGATGTGGAGGAATACCGAAGGCGAAGGCAGCCCCT<br>TGGGAATGTACTGACGCTCATGTGCGAAAGCGTGGGGAGCAAACAGGATTAGATACCCTGGTAGTCCACGCTGTAAACGCTGTCGATTTG<br>GGGATTGGACTTTATGTTTGGTGCCCGAAGCTAACGTGATAAATCGACCGCCTGGGGAGTACGGCCGCAAGGTTAAAACTCAAATGAATT<br>GACGGGGGCCCGCACAAGCGGTGGAGCATGTGGTTTAATTCGATGCAACGCGAAGAACCTTACCTACTCTTGACATCCTAAGAAGGGCTC<br>AGAGATGAGCTTGTGCCTTCGGGAACTTAGAGACAGGTGCTGCATGGCTGTCGTCAGCTCGTGTTGTGAAATGTTGGGTTAAGTCCCGCAA<br>CGAGCGCAACCCTTATCCTTTGTTGCCAGCGATTTGGTCGGGAACTCAAAGGAGACTGCCAGTGACAAACTGGAGGAAGGTGGGGATGAC<br>GTCAAGTCATCATGGCCCTTACGAGTAGGGCTACACACGTGCTACAATGGTGCATACAGAGGGCGGCGAGACGGCGACGTTGAGCGAATC<br>TCAGAAAGTGCATCTAAGTCCGGATTGGAGTCTGCAACTCGACTCCATGAAGTCGGAATCGCTAGTAATCGCAAATCAGAATGTTGCGGT<br>GAATACGTTCCCGGGCCTTGTACACACCGCCCGTCACACCATGGGAGTGGGTTGTACCAGAAGTGGATAGCTTAACGGAAACGAGGGCGT<br>TCACCACGGTATGATTCATGACTGGGGTG	99.73	*Gemella palaticanis*
CTCAGGACGAACGCTGGCGGCGTGCCTAATACATGCAAGTCGAGCGGAGTTGAATAAGTGCTTGCACTAATTCAACTTAGCGGCGAACGG<br>GTGAGTAACACGTAAAGAACCTGCCTCATAGACTGGGACAACTACTGGAAACGGTAGCTAATACCAGATAACATATTTTTCTGCATGGAA<br>GAATATTGAAAGTCGGTCATGCTGACACTATGAGATGGCTTTGCGGTGCATTAGCTAGTTGGTAGGGTAAAGGCCTACCAAGGCGACGAT<br>GCATAGCCGACCTGAGAGGGTGATCGGCCACACTGGGACTGAGACACGGCCCAGACTCCTACGGGAGGCAGCAGTAGGGAATCTTCCGC<br>AATGGACGAAAGTCTGACGGAGCAACGCCGCGTGAGTGAAGAAGGATTTCGGTTCGTAAAACTCTGTTGTTAGGGAAGAATGATTGTATA<br>GTAACTATATACAGTAGAGACGGTACCTAACCAGAAAGCCACGGCTAACTACGTGCCAGCAGCCGCGGTAATACGTAGGTGGCAAGCGTT<br>GTCCGGAATTATTGGGCGTAAAGCGCGCGCAGGTGGTTTAAAAAGTCTGATGTGAAAGCCCACGGCTCAACCGTGGAAGGTCATTGGAAA<br>CTGTTAAACTTGAGTGCAGGAGAGAAAAGTGGAATTCCTAGTGTAGCGGTGAAATGCGTAGAGATTAGGAGGAACACCAGTGGCGAAGG<br>CGGCTTTTTGGCCTGTAACTGACACTGAGGCGCGAAAGCGTGGGGAGCAAACAGGATTAGATACCCTGGTAGTCCACGCTGTAAACGATG<br>AGTGCTAAGTGTTGGAGTCATAAGACTTCAGTGCTGCAGCTAACGCATTAAGCACTCCGCCTGGGGAGTACGGTCGCAAGACTGAAACTC<br>AAAGGAATTGACGGGGACCCGCACAAGCGGTGGAGTATGTGGTTTAATTCGAAGCAACGCGAAGAACCTTACCAAGTCTTGACATACAGT<br>GAAGATTTAAGAGATTAGATTGTTTATACCTATGGTATAACACTGATACAGGTGGTGCATGGTTGTCGTCAGCTCGTGTCGTGAGATGTTG<br>GGTTAAGTCCCGCAACGAGCGCAACCCTTATATCTAGTTGCCAGCAGTAAGATGGGGACTCTAGATAGACTGCCAGTGATAAACTGGAGG<br>AAGGTGGGGATGACGTCAAATCATCATGCCCCTTATGACTTGGGCTACACACGTACTACAATGGATAGGAACAAAGAGAAGCAACCTCGC<br>AAGAGCAAGCCAACCTCATAAAACTATTCTCAGTTCGGATTGTAGTCTGCAACTCGACTACATGAAGCTGGAATCGCTAGTAATCGCGAA<br>TCAGAATGTCGCGGTGAATACGTTCCCGGGTCTTGTACACACCGCCCGTCACACCACGAGAGTTTGTAACACCCGAAGACGGTGGCCTAA<br>CCGCAAGGAGGGAGCCGGTCACGGTGGGATAGATGATTGGGGTGAAGTCTAC	100	*Neisseria zoodegmatis*

CTCAGATTGAACGCTGGCGGCAGGCTTAACACATGCAAGTCGAACGGTAGCAGGAAGAAAGCTTGCTTTTTTTGCTGACGAGTGGCGGAC<br>GGGTGAGTAATGCTTGGGAATCTGGCTTATGGAGGGGGATAACTGTGGGAAACTGCAGCTAATACCGCGTATAATCGAGAGATGAAAGGG<br>TGGGACCGTAAGGCCACTTGCCATAAGATGAGCCCAAGTGGGATTAGGTAGTTGGTGGGGTAAAGGCCTACCAAGCCTGCGATCTCTAGC<br>TGGTCTGAGAGGATGGCCAGCCACACTGGGACTGAGACACGGCCCAGACTCCTACGGGAGGCAGCAGTGGGGAATATTGCGCAATGGGG<br>GGAACCCTGACGCAGCCATGCCGCGTGAATGAAGAAGGCCTTCGGGTTGTAAAGTTCTTTCGGTGATGAGGAAGGGGTATTATTGAATAG<br>ATAATATCATTGACGTTAATTACAGAAGAAGCACCGGCTAACTCCGTGCCAGCAGCCGCGGTAATACGGAGGGTGCGAGCGTTAATCGGA<br>ATAACTGGGCGTAAAGGGCACGCAGGCGGACTTTTAAGTGAGATGTGAAATCCCCGAGCTTAACTTGGGAACTGCATTTCAGACTGGGAG<br>TCTAGAGTACTTTAGGGAGGGGTAGAATTCCACGTGTAGCGGTGAAATGCGTAGAGATGTGGAGGAATACCGAAGGCGAAGGCAGCCCCT<br>TGGGAATGTACTGACGCTCATGTGCGAAAGCGTGGGGAGCAAACAGGATTAGATACCCTGGTAGTCCACGCTGTAAACGCTGTCGATTTG<br>GGGATTGGACTTTATGTTTGGTGCCCGAAGCTAACGTGATAAATCGACCGCCTGGGGAGTACGGCCGCAAGGTTAAAACTCAAATGAATT<br>GACGGGGGCCCGCACAAGCGGTGGAGCATGTGGTTTAATTCGATGCAACGCGAAGAACCTTACCTACTCTTGACATCCTAAGAAGGGCTC<br>AGAGATGAGCTTGTGCCTTCGGGAACTTAGAGACAGGTGCTGCATGGCTGTCGTCAGCTCGTGTTGTGAAATGTTGGGTTAAGTCCCGCAA<br>CGAGCGCAACCCTTATCCTTTGTTGCCAGCGATTTGGTCGGGAACTCAAAGGAGACTGCCAGTGACAAACTGGAGGAAGGTGGGGATGAC<br>GTCAAGTCATCATGGCCCTTACGAGTAGGGCTACACACGTGCTACAATGGTGCATACAGAGGGCGGCGAGACGGCGACGTTGAGCGAATC<br>TCAGAAAGTGCATCTAAGTCCGGATTGGAGTCTGCAACTCGACTCCATGAAGTCGGAATCGCTAGTAATCGCAAATCAGAATGTTGCGGT<br>GAATACGTTCCCGGGCCTTGTACACACCGCCCGTCACACCATGGGAGTGGGTTGTACCAGAAGTGGATAGCTTAACGGAAACGAGGGCGT<br>TCACCACGGTATGATTCATGACTGGGGTG	100	*Pasteurella canis*
TTTAGAGTTTGGATCATGGCTCAGATTGAACGCTGGCGGCAGGCTTAACACATGCAAGTCGAACGGTAGCAGGAAGAAAGCTTGCTTTCTT<br>TGCTGACGAGTGGCGGACGGGTGAGTAATGCTTGGGAATCTGGCTTATGGAGGGGGATAACTGCGGGAAACTGCAGCTAATACCGCGTAT<br>TATCGAGAGATGAAAGGGTGGGACCTTAGGGCCACCTGCCATAAGATGAGCCCAAGTGGGATTAGGTAGTTGGTGGGGTAAAGGCCTACC<br>AAGCCGACGATCTCTAGCTGGTCTGAGAGGATGACCAGCCACACTGGGACTGAGACACGGCCCAGACTCCTACGGGAGGCAGCAGTGGG<br>GAATATTGCGCAATGGGGGGAACCCTGACGCAGCCATGCCGCGTGAATGAAGAAGGCCTTCGGGTTGTAAAGTTCTTTCGGTAATGAGGA<br>AGGGGTATTATTGAATAGATAATATCATTGACGTTAATTACAGAAGAAGCACCGGCTAACTCCGTGCCAGCAGCCGCGGTAATACGGAGG<br>GTGCGAGCGTTAATCGGAATAACTGGGCGTAAAGGGCACGCAGGCGGGCTTTTAAGTGAGATGTGAAATCCCCGAGCTTAACTTGGGAAC<br>TGCATTTCAGACTGGGAGTCTAGAGTACTTTAGGGAGGGGTAGAATTCCACGTGTAGCGGTGAAATGCGTAGAGATGTGGAGGAATACCG<br>AAGGCGAAGGCAGCCCCTTGGGAATGTACTGACGCTCATGTGCGAAAAGGMRTGRGGAGCAAACAGGATTAGATACCCTGGTAGTCCAC<br>GCTGTAAACGCTGTCGATTTGGGGATTGGGCTTTATGCTTGGTGCCCGAAGCTAACGTGATAAATCGACCGCCTGGGGAGTACGGCCGCA<br>AGGTTAAAACTCAAATGAATTGACGGGGGCCCGCACAAGCGGTGGAGCATGTGGTTTAATTCGATGCAACGCGAAGAACCTTACCTACTC<br>TTGACATCCTAAGAAGAGCTCAGAGATGAGCTTGTGCCTTCGGGAACTTAGAGACAGGTGCTGCATGGCTGTCGTCAGCTCGTGTTGTGAA<br>ATGTTGGGTTAAGTCCCGCAACGAGCGCAACCCTTATCCTTTGTTGCCAGCGATTTGGTCGGGAACTCAAAGGAGACTGCCAGTGACAAA<br>CTGGAGGAAGGTGGGGATGACGTCAAGTCATCATGGCCCTTACGAGTAGGGCTACACACGTGCTACAATGGTGCATACAGAGGGCGGCGA<br>GACGGCGACGTTGAGCGAATCTCATAAAGTGCATCTAAGTCCGGATTGGAGTCTGCAACTCGACTCCATGAAGTCGGAATCGCTAGTAAT<br>CGCAAATCAGAATGTTGCGGTGAATACGTTCCCGGGCCTTGTACACACCGCCCGTCACACCATGGGAGTGGGTTGTACCAGAAGTGGATA<br>GCTTAACGGAAACGAGGGCGTTCACCACGGTATGATTCATGACTGGGGTGAAGTCGTACAG	99.53	*Pasteurella multocida subsp. multocida*

CTCAGATTGAACGCTGGCGGCAGGCTTAACACATGCAAGTCGAACGATGATTACTAGCTTGCTAGTATGATTAGTGGCGAACGGGTGAGT<br>AATGCTTAGGAATCTGCCTATTAGTGGGGGACAACATTCCGAAAGGAATGCTAATACCGCATACGTCCTACGGGAGAAAGCAGGGGATCG<br>CAAGACCTTGCGCTAATAGATGAGCCTAAGTCGGATTAGCTAGTTGGTGGGGTAAAGGCCTACCAAGGCGACGATCTGTAGCTGGTCTGA<br>GAGGATGATCAGCCACACTGGGACTGAGACACGGCCCAGACTCCTACGGGAGGCAGCAGTGGGGAATATTGGACAATGGGCGAAAGCCT<br>GATCCAGCCATGCCGCGTGTGTGAAGAAGGCCTTTTGGTTGTAAAGCACTTTAAGTGGGGAGGAAAAGCTAATAATTAATACCTATTAGC<br>CCTGACGTTACCCACAGAATAAGCACCGGCTAACTCTGTGCCAGCAGCCGCGGTAATACAGAGGGTGCAAGCGTTAATCGGAATTACTGG<br>GCGTAAAGCGAGCGTAGGTGGTTACTTAAGTCAGATGTGAAAGCCCCGGGCTTAACCTGGGAACTGCATCTGATACTGGGTAACTAGAGT<br>AGGTGAGAGGGAAGTAGAATTCCAGGTGTAGCGGTGAAATGCGTAGAGATCTGGAGGAATACCGATGGCGAAGGCAGCTTCCTGGCATC<br>ATACTGACACTGAGGTTCGAAAGCGTGGGTAGCAAACAGGATTAGATACCCTGGTAGTCCACGCCGTAAACGATGTCTACCAGTCGTTGG<br>GTCTTTTAAAGACTTAGTGACGCAGTTAACGCAATAAGTAGACCGCCTGGGGAGTACGGCCGCAAGGTTAAAACTCAAATGAATTGACGG<br>GGGCCCGCACAAGCGGTGGAGCATGTGGTTTAATTCGATGCAACGCGAAGAACCTTACCTGGTCTTGACATAGTAGGAATCCTGCAGAGA<br>TGCGGGAGTGCCTTCGGGAATCTACATACAGGTGCTGCATGGCTGTCGTCAGCTCGTGTCGTGAGATGTTGGGTTAAGTCCCGCAACGAGC<br>GCAACCCTTTTCCTTAGTTACCAGCGGGTTATGCCGGGAACTCTAAGGATACTGCCAGTGACAAACTGGAGGAAGGCGGGGACGACGTCA<br>AGTCATCATGGCCCTTACGACCAGGGCTACACACGTGCTACAATGGTAGGTACAAAGGGTTGCTACACAGCGATGTGATGCTAATCTCAA<br>AAAGCCTATCGTAGTCCGGATTGGAGTCTGCAACTCGACTCCATGAAGTCGGAATCGCTAGTAATCGCAGATCAGAATGCTGCGGTGAAT<br>ACGTTCCCGGGCCTTGTACACACCGCCCGTCACACCATGGGAGTTGATCTCACCAGAAGTGGGTAGCCTAACGCAAGAGGGCGCTCACCA<br>CGGTGGGGTCGATGACTGGGGTGAAGTCGTACAAGGG	99.53	*Moraxella sp.*
GGCGGCAGGCCTAACACATGCAAGTCGAGCGGATGAAGGGAGCTTGCTCCTGGATTCAGCGGCGGACGGGTGAGTAATGCCTAGGAATCT<br>GCCTGGTAGTGGGGGATAACGTCCGGAAACGGGCGCTAATACCGCATACGTCCTGAGGGAGAAAGTGGGGGATCTTCGGACCTCACGCTA<br>TCAGATGAGCCTAGGTCGGATTAGCTAGTTGGTGGGGTAAAGGCCTACCAAGGCGACGATCCGTAACTGGTCTGAGAGGATGATCAGTCA<br>CACTGGAACTGAGACACGGTCCAGACTCCTACGGGAGGCAGCAGTGGGGAATATTGGACAATGGGCGAAAGCCTGATCCAGCCATGCCG<br>CGTGTGTGAAGAAGGTCTTCGGATTGTAAAGCACTTTAAGTTGGGAGGAAGGGCAGTAAGTTAATACCTTGCTGTTTTGACGTTACCAACA<br>GAATAAGCACCGGCTAACTTCGTGCCAGCAGCCGCGGTAATACGAAGGGTGCAAGCGTTAATCGGAATTACTGGGCGTAAAGCGCGCGTA<br>GGTGGTTCAGCAAGTTGGATGTGAAATCCCCGGGCTCAACCTGGGAACTGCATCCAAAACTACTGAGCTAGAGTACGGTAGAGGGTGGTG<br>GAATTTCCTGTGTAGCGGTGAAATGCGTAGATATAGGAAGGAACACCAGTGGCGAAGGCGACCACCTGGACTGATACTGACACTGAGGTG<br>CGAAAGCGTGGGGAGCAAACAGGATTAGATACCCTGGTAGTCCACGCCGTAAACGATGTCGACTAGCCGTTGGGATCCTTGAGATCTTAG<br>TGGCGCAGCTAACGCGATAAGTCGACCGCCTGGGGAGTACGGCCGCAAGGTTAAAACTCAAATGAATTGACGGGGGCCCGCACAAGCGG<br>TGGAGCATGTGGTTTAATTCGAAGCAACGCGAAGAACCTTACCTGGCCTTGACATGCTGAGAACTTTCCAGAGATGGATTGGTGCCTTCGG<br>GAACTCAGACACAGGTGCTGCATGGCTGTCGTCAGCTCGTGTCGTGAGATGTTGGGTTAAGTCCCGTAACGAGCGCAACCCTTGTCCTTAG<br>TTACCAGCACCTCGGGTGGGCACTCTAAGGAGACTGCCGGTGACAAACCGGAGGAAGGTGGGGATGACGTCAAGTCATCATGGCCCTTAC<br>GGCCAGGGCTACACACGTGCTACAATGGTCGGTACAAAGGGTTGCCAAGCCGCGAGGTGGAGCTAATCCCATAAAACCGATCGTAGTCCG<br>GATCGCAGTCTGCAACTCGACTGCGTGAAGTCGGAATCGCTAGTAATCGTGAATCAGAATGTCACGGTGAATACGTTCCCGGGCCTTGTA<br>CACACCGCCCGTCACACCATGGGAGTGGGTTGCTCCAGAAGTAGCTAGTCTAACCGCAAGGGGGACGGTTACCACGGAGTGATTCAT	100	*Pseudomonas aeruginosa*

ATCATGGCTCAGATTGAACGCTGGCGGCAGGCTTAACACATGCAAGTCGAACGGTAGCAGGGAGAAAGCTTGCTTTCTTTGCTGACGAGT<br>GGCGGACGGGTGAGTAATGCTTGGGAATCTGGCTTATGGAGGGGGATAACTGTGGGAAACTGCAGCTAATACCGCGTATTATCGAGAGAT<br>GAAAGGGTGGGACCTTCGGGCCACCTGCCATGAGATGAGCCCAAGTGGGATTAGGTAGTTGGTGGGGTAAAGGCCTACCAAGCCGACGAT<br>CTCTAGCTGGTCTGAGAGGATGACCAGCCACACTGGGACTGAGACACGGCCCAGACTCCTACGGGAGGCAGCAGTGGGGAATATTGCGC<br>AATGGGGGGAACCCTGACGCAGCCATGCCGCGTGAATGAAGAAGGCCTTCGGGTTGTAAAGTTCTTTCGGTAATGAGGAAGGGGTATTAT<br>TGAATAGATAATATCATTGACGTTAATTACAGAAGAAGCACCGGCTAACTCCGTGCCAGCAGCCGCGGTAATACGGAGGGTGCGAGCGTT<br>AATCGGAATAACTGGGCGTAAAGGGCACGCAGGCGGACTTTTAAGTGAGATGTGAAATCCCCGAGCTTAACTTGGGAACTGCATTTCAGA<br>CTGGGAGTCTAGAGTACTTTAGGGAGGGGTAGAATTCCACGTGTAGCGGTGAAATGCGTAGAGATGTGGAGGAATACCGAAGGCGAAGG<br>CAGCCCCTTGGGAATGTACTGACGCTCATGTGCGAAAGCGTGGGGAGCAAACAGGATTAGATACCCTGGTAGTCCACGCTGTAAACGCTG<br>TCGATTTGGGGATTGGACTTAATGTTTGGTGCCCGAAGCTAACGTGATAAATCGACCGCCTGGGGAGTACGGCCGCAAGGTTAAAACTCA<br>AATGAATTGACGGGGGCCCGCACAAGCGGTGGAGCATGTGGTTTAATTCGATGCAACGCGAAGAACCTTACCTACTCTTGACATCCTAAG<br>AAGAGCTCAGAGATGAGCTTGTGCCTTCGGGAACTTAGAGACAGGTGCTGCATGGCTGTCGTCAGCTCGTGTTGTGAAATGTTGGGTTAAG<br>TCCCGCAACGAGCGCAACCCTTATCCTTTGTTGCCAGCGATTTGGTCGGGAACTCAAAGGAGACTGCCAGTGATAAACTGGAGGAAGGTG<br>GGGATGACGTCAAGTCATCATGGCCCTTACGAGTAGGGCTACACACGTGCTACAATGGTGCATACAGAGGGCGGCGAGACGGCGACGTTG<br>AGCGAATCTCATAAAGTGCATCTAAGTCCGGATTGGAGTCTGCAACTCGACTCCATGAAGTCGGAATCGCTAGTAATCGCAAATCAGAAT<br>GTTGCGGTGAATACGTTCCCGGGCCTTGTACACACCGCCCGTCACACCATGGGAGTGGGTTGTACCAGAAGTGGATAGCTTAACGGAAAC<br>GAGGGCGTTCACCACGGTATGATTCATGACTGGGGTGAAGTCGTACA	99.46	*Pasteurella multocida subsp. multocida*
TTTAGAGTTTGAATCATGGCTCAGGATGAACGCTGGCGGCGTGCCTAATACATGCAAGTCGAGCGAACAGATAAGGAGCTTGCTCCTTTG<br>ACGTTAGCGGCGGACGGGTGAGTAACACGTGGGTAACCTACCTATAAGACTGGAATAACTCCGGGAAACCGGGGCTAATGCCGGATAAC<br>ATGTTGAACCGCATGGTTCTACAGTGAAAGACGGTCTTGCTGTCACTTATAGATGGACCCGCGCCGTATTAGCTAGTTGGTGGGGTAACGG<br>CCTACCAAGGCGACGATACGTAGCCGACCTGAGAGGGTGATCGGCCACACTGGAACTGAGACACGGTCCAGACTCCTACGGGAGGCAGC<br>AGTAGGGAATCTTCCGCAATGGGCGAAAGCCTGACGGAGCAACGCCGCGTGAGTGATGAAGGTCTTCGGATCGTAAAGCTCTGTTGTTAG<br>GGAAGAACAAATGTGTAAGTAACTGTGCACATCTTGACGGTACCTAACCAGAAAGCCACGGCTAACTACGTGCCAGCAGCCGCGGTAATA<br>CGTAGGKGGSAAGCGYTATCCGGAATTATTGGGCGTAAAGCGCGCGTAGGCGGTTTTTTAAGTCTGATGTGAAAGCCCACGGCTCAACCG<br>TGGAGGGTCATTGGAAACTGGAAAACTTGAGTGCAGAAGAGGAAAGTGGAATTCCATGTGTAGCGGTGAAATGCGCAGAGATATGGAGG<br>AACACCAGTGGCGAAGGCGGCTTTCTGGTCTGCAACTGACGCTGATGTGCGAAAGCGWRGSSATCAAACAGGATTAGATACCCTGGTAGT<br>CCACGCCGTAAACGATGAGTGCTAAGTGTTAGGGGGTTTCCGCCCCTTAGTGCTGCAGCTAACGCATTAAGCACTCCGCCTGGGGAGTAC<br>GGTCGCAAGACTGAAACTCAAAGGAATTGACGGGGACCCGCACAAGCGGTGGAGCATGTGGTTTAATTCGAAGCAACGCGAAGAACCTT<br>ACCAAATCTTGACATCCTTTGACCGCTCTAGAGATAGAGTTTTCCTCTTCGGAGGACAAAGTGACAGGTGGTGCATGGTTGTCGTCAGCTC<br>GTGTCGTGAGATGTTGGGTTAAGTCCCGCAACGAGCGCAACCCTTGAACTTAGTTGCCATCATTCAGTTGGGCACTCTAAGTTGACTGCCG<br>GTGACAAACCGGAGGAAGGTGGGGATGACGTCAAATCATCATGCCCCTTATGATTTGGGCTACACACGTGCTACAATGGACAATACAAAG<br>GGCAGCGAAACCGCGAGGTCAAGCAAATCCCATAAAGTTGTTCTCAGTTCGGATTGTAGTCTGCAACTCGACTACATGAAGCTGGAATCG<br>CTAGTAATCGTAGATCAGCATGCTACGGTGAATACGTTCCCGGGTCTTGTACACACCGCCCGTCACACCACGAGAGTTTGTAACACCCGA<br>AGCCGGTGGAGTAACCATTTTGGAGCTAGCCGTCGAAGGTGGGACAAATGATTGGGGTGAAGTCGTACAGGGGAAACCGTAAA	100	*Staphylococcus pseudintermedius*

ATCATGGCTCAGATTGAACGCTGGCGGCAGGCTTAACACATGCAAGTCGAACGGTAGCAGGGAGAAAGCTTGCTTTCTTTGCTGACGAGT<br>GGCGGACGGGTGAGTAATGCTTGGGAATCTGGCTTATGGAGGGGGATAACTGTGGGAAACTGCAGCTAATACCGCGTATTATCGAGAGAT<br>GAAAGGGTGGGACCTTCGGGCCACCTGCCATGAGATGAGCCCAAGTGGGATTAGGTAGTTGGTGGGGTAAAGGCCTACCAAGCCGACGAT<br>CTCTAGCTGGTCTGAGAGGATGACCAGCCACACTGGGACTGAGACACGGCCCAGACTCCTACGGGAGGCAGCAGTGGGGAATATTGCGC<br>AATGGGGGGAACCCTGACGCAGCCATGCCGCGTGAATGAAGAAGGCCTTCGGGTTGTAAAGTTCTTTCGGTAATGAGGAAGGGGTATTAT<br>TGAATAGATAATATCATTGACGTTAATTACAGAAGAAGCACCGGCTAACTCCGTGCCAGCAGCCGCGGTAATACGGAGGGTGCGAGCGTT<br>AATCGGAATAACTGGGCGTAAAGGGCACGCAGGCGGACTTTTAAGTGAGATGTGAAATCCCCGAGCTTAACTTGGGAACTGCATTTCAGA<br>CTGGGAGTCTAGAGTACTTTAGGGAGGGGTAGAATTCCACGTGTAGCGGTGAAATGCGTAGAGATGTGGAGGAATACCGAAGGCGAAGG<br>CAGCCCCTTGGGAATGTACTGACGCTCATGTGCGAAAGCGTGGGGAGCAAACAGGATTAGATACCCTGGTAGTCCACGCTGTAAACGCTG<br>TCGATTTGGGGATTGGACTTAATGTTTGGTGCCCGAAGCTAACGTGATAAATCGACCGCCTGGGGAGTACGGCCGCAAGGTTAAAACTCA<br>AATGAATTGACGGGGGCCCGCACAAGCGGTGGAGCATGTGGTTTAATTCGATGCAACGCGAAGAACCTTACCTACTCTTGACATCCTAAG<br>AAGAGCTCAGAGATGAGCTTGTGCCTTCGGGAACTTAGAGACAGGTGCTGCATGGCTGTCGTCAGCTCGTGTTGTGAAATGTTGGGTTAAG<br>TCCCGCAACGAGCGCAACCCTTATCCTTTGTTGCCAGCGATTTGGTCGGGAACTCAAAGGAGACTGCCAGTGATAAACTGGAGGAAGGTG<br>GGGATGACGTCAAGTCATCATGGCCCTTACGAGTAGGGCTACACACGTGCTACAATGGTGCATACAGAGGGCGGCGAGACGGCGACGTTG<br>AGCGAATCTCATAAAGTGCATCTAAGTCCGGATTGGAGTCTGCAACTCGACTCCATGAAGTCGGAATCGCTAGTAATCGCAAATCAGAAT<br>GTTGCGGTGAATACGTTCCCGGGCCTTGTACACACCGCCCGTCACACCATGGGAGTGGGTTGTACCAGAAGTGGATAGCTTAACGGAAAC<br>GAGGGCGTTCACCACGGTATGATTCATGACTGGGGTGAAGTCGTACA	99.46	*Pasteurella multocida subsp. multocida*
TTAGAGTTTGGAATCATGGCTCAGATTGAACGCTGGCGGCATGCTTTACACATGCAAGTCGGACGGCAGCACAGAGAAGCTTGCTTCTTG<br>GGTGGCGAGTGGCGAACGGGTGAGTAATGCATCGGAACGTACCGAGTAGTGGGGGATAACTGTCCGAAAGGATGGCTAATACCGCATAC<br>GCTTTGAGAAGGAAAGCAGGGGCTCTTCGGACCTTGCGCTATTCGAGCGGCCGATGTCTGATTAGCTGGTTGGTGGGGTAAAGGCCTACC<br>AAGGCGACGATCAGTAGCGGGTCTGAGAGGATGATCCGCCACACTGGGACTGAGACACGGCCCAGACTCCTACGGGAGGCAGCAGTGGG<br>GAATTTTGGACAATGGGCGCAAGCCTGATCCAGCCATGCCGCGTGTCTGAAGAAGGCCTTCGGGTTGTAAAGGACTTTTGTCAGGGAAGA<br>AAAGGTTGTGGTTAATACCCATGACTGATGACGGTACCTGAAGAATAAGCACCGGCTAACTACGTGCCAGCAGCCGCGGTAATACGTAGG<br>GKGCSARCGTTAATCGGAATTACTGGGCGTAAAGCGGGCGCAGACGGTTACTTAAGTCAGATGTGAAATCCCCGGGCTCAACCTGGGAAC<br>TGCGTTTGAAACTGGGTGACTAGAGTATGTCAGAGGGGGGTAGAATTCCACGTGTAGCAGTGAAATGCGTAGAGATGTGGAGGAATACCG<br>ATGGCGAAGGCAGCCCCCTGGGATAATACTGACGTTCATGCCCGAAAGMGKRRGTWSCAAACAGGATTAGATACCCTGGTAGTCCACGC<br>CCTAAACGATGTCAATTAGCTGTTGGGCAACTTGATTGCTTAGTAGCGTAGCTAACGCGTGAAATTGACCGCCTGGGGAGTACGGTCGCA<br>AGATTAAAACTCAAAGGAATTGACGGGGACCCGCACAAGCGGTGGATGATGTGGATTAATTCGATGCAACGCGAAGAACCTTACCTGGTC<br>TTGACATGTACGGAATCCTCCAGAGACGGAGGAGTGCCTTCGGGAGCCGTAACACAGGTGCTGCATGGCTGTCGTCAGCTCGTGTCGTGA<br>GATGTTGGGTTAAGTCCCGCAACGAGCGCAACCCTTGTCATTAGTTGCCATCATTTGGTTGGGCACTCTAATGAGACTGCCGGTGACAAGC<br>CGGAGGAAGGTGGGGATGACGTCAAGTCCTCATGGCCCTTATGACCAGGGCTTCACACGTCATACAATGGTCGGTACAGAGGGTAGCCAA<br>GCCGCGAGGTGGAGCCAATCCCACAAAACCGATCGTAGTCCGGATTGCACTCTGCAACTCGAGTGCATGAAGTCGGAATCGCTAGTAATC<br>GCAGGTCAGCATACTGCGGTGAATACGTTCCCGGGTCTTGTACACACCGCCCGTCACACCATGGGAGTGGGGGATACCAGAAGTAGGTAG<br>GGTAACCGCAAGGAGCCCGCTTACCACGGTATGCTTCATGACTGGGGTGAAGTCGTACAGGGGAAACCGTAAAT	99.2	*Neisseria zoodegmatis*

TTTAGAGTTTGGATCATGGCTCAGAACGAACGCTGGCGGCAGGCCTAACACATGCAAGTCGAGCGAACCCTTCGGGGTTAGCGGCGGACG<br>GGTGAGTAACGCGTGGGAACGTGCCCTTTGCTACGGAATAGCCCCGGGAAACTGGGAGTAATACCGTATACGCCCTTAGGGGGAAAGATT<br>TATCGGCAAGGGATCGGCCCGCGTTGGATTAGGTAGTTGGTGGGGTAATGGCCTACCAAGCCGACGATCCATAGCTGGTTTGAGAGGATG<br>ATCAGCCACACTGGGACTGAGACACGGCCCAGACTCCTACGGGAGGCAGCAGTGGGGAATCTTAGACAATGGGGGCAACCCTGATCTAG<br>CCATGCCGCGTGAGTGATGAAGGCCCTAGGGTTGTAAAGCTCTTTCAGCTGGGAAGATAATGACGGTACCAGCAGAAGAAGCCCCGGCTA<br>ACTCCGTGCCAGCAGCCGCGGTAATACKGRGGGGGCTAGCGKTGTTCGGAATTACTGGGCGTAAAGCGCACGTAGGCGGACCAGAAAGTT<br>GGGGGTGAAATCCCGGGGCTCAACCTCGGAACTGCCTTCAAAACTATTGGTCTGGAGTTCGAGAGAGGTGAGTGGAATTCCGAGTGTAGA<br>GGTGAAATTCGTAGATATTCGGAGGAACACCAGTGGCGAAGGCGGCTCACTGGCTCGATACTGACGCTGAGGTGCGAAAGMGWRGGSMG<br>CAAACAGGATTAGATACCCTGGTAGTCCACGCCGTAAACGATGAATGCCAGTCGTCGGGCAGCATGCTGTTCGGTGACACACCTAACGGA<br>TTAAGCATTCCGCCTGGGGAGTACGGTCGCAAGATTAAAACTCAAAGGAATTGACGGGGGCCCGCACAAGCGGTGGAGCATGTGGTTTAA<br>TTCGAAGCAACGCGCAGAACCTTACCAACCCTTGACATCCCCGGACCGGCCTGGAGACAGGTCTTCCACTTCGGTGGCCGGGTGACAGGT<br>GCTGCATGGCTGTCGTCAGCTCGTGTCGTGAGATGTTCGGTTAAGTCCGGCAACGAGCGCAACCCACACTCTTAGTTGCCAGCATTTGGTT<br>GGGCACTCTAAGAGAACTGCCGATGATAAGTCGGAGGAAGGTGTGGATGACGTCAAGTCCTCATGGCCCTTACGGGTTGGGCTACACACG<br>TGCTACAATGGTGGTGACAGTGGGTTAATCCCCAAAAGCCATCTCAGTTCGGATTGGGGTCTGCAACTCGACCCCATGAAGTTGGAATCG<br>CTAGTAATCGCGGAACAGCATGCCGCGGTGAATACGTTCCCGGGCCTTGTACACACCGCCCGTCACACCATGGGAGTTGGGTCTACCCGA<br>CGGCCGTGCGCTAACCAGCAATGGGGGCAGCGGACCACGGTAGGCTCAGCGACTGGGGTGAAGTCGTACAGGGGTAACCGTAAA	99.16	*Paracoccus communis*
CTCAGATTGAACGCTGGCGGCAGGCTTAACACATGCAAGTCGAACGGTAGCAGGGAGAAAGCTTGCTTTCTTTGCTGACGAGTGGCGGAC<br>GGGTGAGTAATGCTTGGGAATCTGGCTTATGGAGGGGGATAACTGTGGGAAACTGCAGCTAATACCGCGTATTATCGGAAGATGAAAGGG<br>TGGGACCTTCGGGCCACCTGCCATAAGATGAGCCCAAGTGGGATTAGGTAGTTGGTGGGGTAAGGGCCTACCAAGCCGACGATCTCTAGC<br>TGGTCTGAGAGGATGACCAGCCACACTGGGACTGAGACACGGCCCAGACTCCTACGGGAGGCAGCAGTGGGGAATATTGCGCAATGGGG<br>GGAACCCTGACGCAGCCATGCCGCGTGAATGAAGAAGGCCTTCGGGTTGTAAAGTTCTTTCGGTAATGAGGAAGGGGTATTATTGAATAG<br>ATAATATCATTGACGTTAATTACAGAAGAAGCACCGGCTAACTCCGTGCCAGCAGCCGCGGTAATACGGAGGGTGCGAGCGTTAATCGGA<br>ATAACTGGGCGTAAAGGGCACGCAGGCGGACTTTTAAGTGAGATGTGAAATCCCCGAGCTTAACTTGGGAACTGCATTTCAGACTGGGAG<br>TCTAGAGTACTTTAGGGAGGGGTAGAATTCCACGTGTAGCGGTGAAATGCGTAGAGATGTGGAGGAATACCGAAGGCGAAGGCAGCCCCT<br>TGGGAATGTACTGACGCTCATGTGCGAAAGCGTGGGGAGCAAACAGGATTAGATACCCTGGTAGTCCACGCTGTAAACGCTGTCGATTTG<br>GGGATTGGGCTTTATGCTTGGTGCCCGAAGCTAACGTGATAAATCGACCGCCTGGGGAGTACGGCCGCAAGGTTAAAACTCAAATGAATT<br>GACGGGGGCCCGCACAAGCGGTGGAGCATGTGGTTTAATTCGATGCAACGCGAAGAACCTTACCTACTCTTGACATCCTAAGAAGAGCTC<br>AGAGATGAGCTTGTGCCTTCGGGAACTTAGAGACAGGTGCTGCATGGCTGTCGTCAGCTCGTGTTGTGAAATGTTGGGTTAAGTCCCGCAA<br>CGAGCGCAACCCTTATCCTTTGTTGCCAGCGATTTGGTCGGGAACTCAAAGGAGACTGCCAGTGACAAACTGGAGGAAGGTGGGGATGAC<br>GTCAAGTCATCATGGCCCTTACGAGTAGGGCTACACACGTGCTACAATGGTGCATACAGAGGGCGGCGAGACGGCGACGTTGAGCGAATC<br>TCATAAAGTGCATCTAAGTCCGGATTGGAGTCTGCAACTCGACTCCATGAAGTCGGAATCGCTAGTAATCGCAAATCAGAATGTTGCGGT<br>GAATACGTTCCCGGGCCTTGTACACACCGCCCGTCACACCATGGGAGTGGGTTGTACCAGAAGTGGATAGCTTAACGGAAACGAGGGCGT<br>TCACCACGGTATGATTCATGACTGGGGTG	99.59	*Pasteurella multocida subsp. multocida*

CATTAGAGTTTGGATCATGGCTCAGATTGAACGCTGGCGGCATGCTTTACACATGCAAGTCGGACGGCAGCACAGAGAAGCTTGCTTCTTG<br>GGTGGCGAGTGGCGAACGGGTGAGTAATGCATCGGAACGTACCGAGTAGTGGGGGATAACTGTCCGAAAGGATGGCTAATACCGCATAC<br>GCTTTGAGAAGGAAAGCAGGGGCTCTTCGGACCTTGCGCTATTCGAGCGGCCGATGTCTGATTAGCTGGTTGGTGGGGTAAAGGCCTACC<br>AAGGCGACGATCAGTAGCGGGTCTGAGAGGATGATCCGCCACACTGGGACTGAGACACGGCCCAGACTCCTACGGGAGGCAGCAGTGGG<br>GAATTTTGGACAATGGGCGCAAGCCTGATCCAGCCATGCCGCGTGTCTGAAGAAGGCCTTCGGGTTGTAAAGGACTTTTGTCAGGGAAGA<br>AAAGGTTGTGGTTAATACCCATGACTGATGACGGTACCTGAAGAATAAGCACCGGCTAACTACGTGCCAGCAGCCGCGGTAATACGTAGG<br>GTSCGASCGTTAATCSGRATTACTGGGCGTAAAGCGGGCGCAGACGGTTACTTAAGTCAGATGTGAAATCCCCGGGCTCAACCTGGGAACT<br>GCGTTTGAAACTGGGTGACTAGAGTATGTCAGAGGGGGGTAGAATTCCACGTGTAGCAGTGAAATGCGTAGAGATGTGGAGGAATACCGA<br>TGGCGAAGGCAGCCCCCTGGGATAATACTGACGTTCATGCCCGAAAGAAMGTKSGTAGCAAACAGGATTAGATACCCTGGTAGTCCACGC<br>CCTAAACGATGTCAATTAGCTGTTGGGCAACTTGATTGCTTAGTAGCGTAGCTAACGCGTGAAATTGACCGCCTGGGGAGTACGGTCGCA<br>AGATTAAAACTCAAAGGAATTGACGGGGACCCGCACAAGCGGTGGATGATGTGGATTAATTCGATGCAACGCGAAGAACCTTACCTGGTC<br>TTGACATGTACGGAATCCTCCAGAGACGGAGGAGTGCCTTCGGGAGCTGTAACACAGGTGCTGCATGGCTGTCGTCAGCTCGTGTCGTGA<br>GATGTTGGGTTAAGTCCCGCAACGAGCGCAACCCTTGTCATTAGTTGCCATCATTTGGTTGGGCACTCTAATGAGACTGCCGGTGACAAGC<br>CGGAGGAAGGTGGGGATGACGTCAAGTCCTCATGGCCCTTATGACCAGGGCTTCACACGTCATACAATGGTCGGTACAGAGGGTAGCCAA<br>GCCGCGAGGTGGAGCCAATCCCACAAAACCGATCGTAGTCCGGATTGCACTCTGCAACTCGAGTGCATGAAGTCGGAATCGCTAGTAATC<br>GCAGGTCAGCATACTGCGGTGAATACGTTCCCGGGTCTTGTACACACCGCCCGTCACACCATGGGAGTGGGGGATACCAGAAGTAGGTAG<br>GGTAACCGCAAGGAGCCCGCTTACCACGGTATGCTTCATGACTGGGGTGAAGTCGTAACAGGGTAACCGTAAA	99.01	*Neisseria zoodegmatis*
ATTACGGTTACCTTGTTACGACTTCACCCCAGTCATGAAGCATACCGTGGTAAGCGGGCTCCTTGCGGTTACCCTACCTACTTCTGGTATC<br>CCCCACTCCCATGGTGTGACGGGCGGTGTGTACAAGACCCGGGAACGTATTCACCGCAGTATGCTGACCTGCGATTACTAGCGATTCCGA<br>CTCCATGCACTCGAGTTGCATTAGAGTTTGGATCTGGCTCAGATTGAACGCTGGCGGCATGCTTTACACATGCAAGTCGGACGGCAGCACA<br>AAGAAGCTTGCTTCTTGGGTGGCGAGTGGCGAACGGGTGAGTAACGCATCGGAACGTACCGAGTAGTGGGGGATAACTGTCCGAAAGGAT<br>GGCTAATACCGCATACGCTTTGAGAAGGAAAGCGGGGGATCTTCGGACCTCGCGCTATTCGAGCGGCCGATGTCTGATTAGCTGGTTGGT<br>GGGGTAAAGGCCTACCAAGGCGACGATCAGTAGCGGGTCTGAGAGGATGATCCGCCACACTGGGACTGAGACACGGCCCAGACTCCTAC<br>GGGAGGCAGCAGTGGGGAATTTTGGACAATGGGCGCAAGCCTGATCCAGCCATGCCGCGTGTCTGAAGAAGGCCTTCGGGTTGTAAAGGA<br>CTTTTGTCAGGGAAGAAAAGCTTGAGGCTAATACCCTTAAGTGATGACGGTACCTGAAGAATAAGCACCGGCTAACTACGTGCCAGCAGC<br>CGCGGTAATACGTAGGGKGCGAGCGTTAATCGGAATTACTGGGCGTAAAGCGGGCGCAGACGGTTACTTAAGTCAGATGTGAAATCCCCG<br>GGCTCAACCTGGGAACTGCGTTTGAAACTGGGTAGCTAGAGTATGTCAGAGGGGGGTAGAATTCCACGTGTAGCAGTGAAATGCGTAGAG<br>ATGTGGAGGAATWCCGATGGCGAAGGCAGCCCCCTGGGATAATACTGACGTTMATGCCSGAAAGCGTRGGTAGCAAACAGGATTAGATA<br>CCCTGGTAGTCCACGCCCTAAACGATGTCAATTAGCTGTTGGGGCACTTGATGCCTTAGTAGCGTAGCTAACGCGTGAAATTGACCGCCTG<br>GGGAGTACGGTCGCAAGATTAAAACTCAAAGGAATTGACGGGGACCCGCACAAGCGGTGGATGATGTGGATTAATTCGATGCAACGCGA<br>AGAACCTTACCTGGTCTTGACATGTACGGAATCCTCCAGAGACGGAGGAGTGCCTTCGGGAGCCGTAACACAGGTGCTGCATGGCTGTCG<br>TCAGCTCGTGTCGTGAGATGTTGGGTTAAGTCCCGCAACGAGCGCAACCCTTGTCATTAGTTGCCATCATTAAGTTGGGCACTCTAATGAG<br>ACTGCCGGTGACAAGCCGGAGGAAGGTGGGGATGACGTCAAGTCCTCATGGCCCTTATGACCAGGGCTTCACACGTCATACAATGGTCGG<br>TACAGAGGGTAGCCAAGCCGCGAGGTGGAGCCAATCCCACAAAACCGATCGTAGTCCGGATTGCACTCTGCAACTCGAGTGCATGAAGTC<br>GGAATCGCTAGTAATCGCAGGTCAGCATACTGCGGTGAATACGTTCCCGGGTCTTGTACACACCGCCCGTCACACCATGGGAGTGGGGGA<br>TACCAGAAGTAGGTAGGGTAACCGCAAGGAGCCCGCTTACCACGGTATGCTTCATGACTGGGGTGAAGTCGTACAGAGGAAACCGTAAA	99.59	*Neisseria dumasiana*

CTCAGGACGAACGCTGGCGGCGTGCTTAACACATGCAAGTCGAACGATGAAGCCCAGCTTGCTGGGTGGATTAGTGGCGAACGGGTGAGT<br>AATACGTGAGTAACCTGCCTTTAACTCTGGGATAAGCCTTGGAAACGAGGTCTAATACCGGATACGACTAGTTCCCGCATGGGATGCTGGT<br>GGAAAGGGATATGTACTGGTTTTAGATGGGCTCACGGCCTATCAGCTTGTTGGTGAGGTAACGGCTCACCAAGGCGACGACGGGTAGCCG<br>GCCTGAGAGGGTGACCGGCCACACTGGGACTGAGACACGGCCCAGACTCCTACGGGAGGCAGCAGTGGGGAATATTGCACAATGGGCGC<br>AAGCCTGATGCAGCGACGCCGCGTGAGGGATGACGGCCTTCGGGTTGTAAACCTCTTTCAGCAGGGAAGAAGCGAAAGTGACGGTACCTG<br>CAGAAGAAGCGCCGGCTAACTACGTGCCAGCAGCCGCGGTAATACGTAGGGCGCGAGCGTTGTCCGGAATTATTGGGCGTAAAGAGCTTG<br>TAGGCGGTTTGTCGCGTCTGCTGTGAAAGCCCGGGGCTTAACCCCGGGTTTGCAGTGGGTACGGGCTAACTAGAGTGCAGTAGGGGAGAC<br>TGGAATTCCTGGTGTAGCGGTGAAATGCGCAGATATCAGGAGGAACACCAATGGCGAAGGCAGGTCTCTGGGCTGTAACTGACGCTGAGA<br>AGCGAAAGCATGGGGAGCAAACAGGATTAGATACCCTGGTAGTCCATGCCGTAAACGTTGGGCACTAGGTGTGGGGGGCATTCCACGTTT<br>TCCGCGCCGTAGCTAACGCATTAAGTGCCCCGCCTGGGGAGTACGGCCGCAAGGCTAAAACTCAAAGAAATTGACGGGGGCCCGCACAA<br>GCGGCGGAGCATGCGGATTAATTCGATGCAACGCGAAGAACCTTACCAAGGCTTGACATATACCGGACCGCTCTAGAGATAGAGTTTCCC<br>TTCGGGGCTGGTATACAGGTGGTGCATGGTTGTCGTCAGCTCGTGTCGTGAGATGTTGGGTTAAGTCCCGCAACGAGCGCAACCCTCGTTC<br>TATGTTGCCAGCACGTAATGGTGGGGACTCATAGGAGACTGCCGGGGTCAACTCGGAGGAAGGTGGGGATGACGTCAAATCATCATGCCC<br>CTTATGTCTTGGGCTTCACGCATGCTACAATGGCCGGTACAAAGGGTTGCGATACTGTGAGGTTGAGCTAATCCCAAAAAGCCGGTCTCAG<br>TTCGGATTGGGGTCTGCAACTCGACCCCATGAAGTCGGAGTCGCTAGTAATCGCAGATCAGCAACGCTGCGGTGAATACGTTCCCGGGCC<br>TTGTACACACCGCCCGTCAAGTCACGAAAGTTGGTAACACCCGAAGCCGATGGCCTAACCCCTTGTGGGAGGGAGTCGTC	99.79	*Rothia nasimurium*
ATGGCTCAGATTGAACGCTGGCGGCAGGCTTAACACATGCAAGTCGAACGGTAGCAGGAGAAAGCTTGCTTTCTTGCTGACGAGTGGCGG<br>ACGGGTGAGTAATGCTTGGGAATCTGGCTTATGGAGGGGGATAACTACTGGAAACGGTAGCTAATACCGCGTAATATCGAGAGATTAAAG<br>ACTGGGACCTACGGGCCAGTTGCCATAAGATGAGCCCAAGTGGGATTAGGTAGTTGGTGGGGTAAAGGCCTACCAAGCCCGCGATCTCTA<br>GCTGGTCTGAGAGGATGACCAGCCACACTGGAACTGAGACACGGTCCAGACTCCTACGGGAGGCAGCAGTGGGGAATATTGCACAATGG<br>GGGGAACCCTGATGCAGCCATGCCGCGTGAATGAAGAAGGCCTTCGGGTTGTAAAGTTCTTTCGGTGGTGAGGAAGGTATCAACTTTAAT<br>AGAGTTGGTAATTGACGTTATCCACAGAAGAAGCACCGGCTAACTCCGTGCCAGCAGCCGCGGTAATACGGAGGGKGCSAGCGTTAATCG<br>GAATGACTGGGCGTAAAGGGCACGCAGGCGGATGCTTAAGTGAGATGTGAAAGCCCCGGGCTTAACCTGGGAATTGCATTTCAGACTGGG<br>CATCTAGAGTATTTTAGGGAGGGGTAGAATTCCACGTGTAGCGGTGAAATGCGTAGAGATGTGGAGGAATACCGAAGGCGAAGGCAGCCC<br>CTTGGGAATATACTGACGCTCATGTGCGAAAGCGTGGGGAGCAAACAGGATTAGATACCCTGGTAGTCCACGCTGTAAACGATGTCGATT<br>TGGGGATTGGGGTATAACTCTGGTGCCCGAAGCTAACGTGATAAATCGACCGCCTGGGGAGTACGGCCGCAAGGTTAAAACTCAAATGAA<br>TTGACGGGGGCCCGCACAAGCGGTGGAGCATGTGGTTTAATTCGATGCAACGCGAAGAACCTTACCTACTCTTGACATCCATGGAAGTTC<br>GCAGAGATGTGAATGTGCCTTCGGGAACCATGAGACAGGTGCTGCATGGCTGTCGTCAGCTCGTGTTGTGAAATGTTGGGTTAAGTCCCGC<br>AACGAGCGCAACCCTTATCCTTTGTTGCCAGCGATTAGGTCGGGAACTCAAAGGAGACTGCCAGTGATAAACTGGAGGAAGGTGGGGATG<br>ACGTCAAGTCATCATGGCCCTTACGAGTAGGGCTACACACGTGCTACAATGGTGCATACAGAGGGAAGCAATATGGCGACATGGAGCAAA<br>TCTCACAAAGTGCATCTAAGTCCGGATTGGAGTCTGCAACTCGACTCCATGAAGTCGGAATCGCTAGTAATCGCAAATCAGAATGTTGCG<br>GTGAATACGTTCCCGGGCCTTGTACACACCGCCCGTCACACCATGGGAGTGGGTTGTACCAGAAGTAGATAGCTTAACCGCAAGGGGGGC<br>GTTTACCACGGTATGATTCATGACTGGGGTGAAGTCGTACAGAGGTAACCGTAAA	99.2	*Frederiksenia canicola*
GGCTGCCTTTCGAGCTCAGCGTCAGTTACAGACCAGAGAGCCGCTTTCGCCACCGGTGTTCCTCCATATATCTACGCATTTCACCGCTACA<br>CATGGAATTCCACTCTCCCCTTCTGCACTCAAGTTCTCCAGTTTCCAAAGCATACATTGGTTGAGCCAATGCCTTTAACTTCAGACTTAAAG<br>AACCGCCTGCGCTCGCTTTACGCCCAATAAATCCGGACAACGCTCGGGACCTACGTATTACCGCGGCTGCTGGCACGTAGTTAGCCGTCC<br>CTTTCTGGTTAGTTACCGTCACATAATGGGTTTTCCACTCCCATTACCGTTCTTCTCTAACAACAGAGCTTTACGATCCGAAAACCTTCTTC<br>ACTCACGCGGCGTTGCTCGGTCAGGGTTCCCCCATTGCCGAAGATTCCCTACTGCTGCCTCCCGTAGGAGTCTGGGCCGTGTCTCAGTCC<br>CAGTGTGGCCGATCACCCTCTCAGGTCGGCTATGTATCGTCGCCTTGGTGAGCCTTTACCTCACCAACTAGCTAATACAACGCAGGTCCAT<br>CTCATAGTGGAGCAGTTGCCCCTTTTAAGTTCTTAACATGTGTTAAGCACTTTTATGCGGTATTAGCTATCGTTTCCAATAGTTATCCCCCG<br>CTATCAGGTAGGTTACCTACGCGTTACTCACCCGTTCGCAACTCCTTAGACTAGTGCAAGCACCTGTCCTCAGCGTTCTACTTGCATGTATT<br>AGGCACGCCGCCAGCGTTCGTCCTGAGCCATATTCCAAACTCTAAAAA	99.06	*Streptococcus sp. (canis)*

TTAGAGTTTGAATCATGGCTCAGGATGAACGCTGGCGGCGTGCCTAATACATGCAAGTCGAGCGAACAGATAAGGAGCTTGCTCCTTTGA<br>CGTTAGCGGCGGACGGGTGAGTAACACGTGGGTAACCTACCTATAAGACTGGAATAACTCCGGGAAACCGGGGCTAATGCCGGATAACAT<br>GTTGAACCGCATGGTTCTACAGTGAAAGACGGTCTTGCTGTCACTTATAGATGGACCCGCGCCGTATTAGCTAGTTGGTGGGGTAACGGCC<br>TACCAAGGCGACGATACGTAGCCGACCTGAGAGGGTGATCGGCCACACTGGAACTGAGACACGGTCCAGACTCCTACGGGAGGCAGCAG<br>TAGGGAATCTTCCGCAATGGGCGAAAGCCTGACGGAGCAACGCCGCGTGAGTGATGAAGGTCTTCGGATCGTAAAGCTCTGTTGTTAGGG<br>AAGAACAAATGTGTAAGTAACTGTGCACATCTTGACGGTACCTAACCAGAAAGCCACGGCTAACTACGTGCCAGCAGCCGCGGTAATACG<br>KRGGKGGSAASCGTTATCCGGRATTATTGGGGCGTAAAGCGCGCGTAGGCGGTTTTTTAAGTCTGATGTGAAAGCCCACGGCTCAACCGTG<br>GAGGGTCATTGGAAACTGGAAAACTTGAGTGCAGAAGAGGAAAGTGGAATTCCATGTGTAGCGGTGAAATGCGCAGAGATATGGAGGAA<br>CACCAGTGGCGAAGGCGGCTTTCTGGTCTGCAACTGACGCTGATGTGCGAAAGCGTGRSGMYCMAACAGGATTAGATACCCTGGTAGTCC<br>ACGCCGTAAACGATGAGTGCTAAGTGTTAGGGGGTTTCCGCCCCTTAGTGCTGCAGCTAACGCATTAAGCACTCCGCCTGGGGAGTACGG<br>TCGCAAGACTGAAACTCAAAGGAATTGACGGGGACCCGCACAAGCGGTGGAGCATGTGGTTTAATTCGAAGCAACGCGAAGAACCTTAC<br>CAAATCTTGACATCCTTTGACCGCTCTAGAGATAGAGTTTTCCTCTTCGGAGGACAAAGTGACAGGTGGTGCATGGTTGTCGTCAGCTCGT<br>GTCGTGAGATGTTGGGTTAAGTCCCGCAACGAGCGCAACCCTTGAACTTAGTTGCCATCATTCAGTTGGGCACTCTAAGTTGACTGCCGGT<br>GACAAACCGGAGGAAGGTGGGGATGACGTCAAATCATCATGCCCCTTATGATTTGGGCTACACACGTGCTACAATGGACAATACAAAGGG<br>CAGCGAAACCGCGAGGTCAAGCAAATCCCATAAAGTTGTTCTCAGTTCGGATTGTAGTCTGCAACTCGACTACATGAAGCTGGAATCGCT<br>AGTAATCGTAGATCAGCATGCTACGGTGAATACGTTCCCGGGTCTTGTACACACCGCCCGTCACACCACGAGAGTTTGTAACACCCGAAG<br>CCGGTGGAGTAACCATTTTGGAGCTAGCCGTCGAAGGTGGGACAAATGATTGGGGTGAAGTCGTACAGGGGGTAACCGTAAA	99.07	*Staphylococcus pseudintermedius*
TTTAGAGTTTGGATCATGGCTCAGATTGAACGCTGGCGGCATGCTTTACACATGCAAGTCGGACGGCAGCACAGAGAAGCTTGCTTCTTGG<br>GTGGCGAGTGGCGAACGGGTGAGTAATGCATCGGAACGTACCGAGTAGTGGGGGATAACTGTCCGAAAGGATGGCTAATACCGCATACG<br>CTTTGAGAAGGAAAGCAGGGGCTCTTCGGACCTTGCGCTATTCGAGCGGCCGATGTCTGATTAGCTGGTTGGTGGGGTAAAGGCCTACCA<br>AGGCGACGATCAGTAGCGGGTCTGAGAGGATGATCCGCCACACTGGGACTGAGACACGGCCCAGACTCCTACGGGAGGCAGCAGTGGGG<br>AATTTTGGACAATGGGCGCAAGCCTGATCCAGCCATGCCGCGTGTCTGAAGAAGGCCTTCGGGTTGTAAAGGACTTTTGTCAGGGAAGAA<br>AAGGTTGTGGTTAATACCCATGACTGATGACGGTACCTGAAGAATAAGCACCGGCTAACTACGTGCCAGCAGCCGCGGTAATACGTAGGC<br>RTKYSMSCGTTAATCGGAATTACTGGGCGTAAAGCGGGCGCAGACGGTTACTTAAGTCAGATGTGAAATCCCCGGGCTCAACCTGGGAAC<br>TGCGTTTGAAACTGGGTGACTAGAGTATGTCAGAGGGGGGTAGAATTCCACGTGTAGCAGTGAAATGCGTAGAGATGTGGAGGAATACCG<br>ATGGCGAAGGCAGCCCCCTGGGATAATACTGACGTTCATGCCCGAAAGGMRTGGGTAGCAAACAGGATTAGATACCCTGGTAGTCCACG<br>CCCTAAACGATGTCAATTAGCTGTTGGGCAACTTGATTGCTTAGTAGCGTAGCTAACGCGTGAAATTGACCGCCTGGGGAGTACGGTCGCA<br>AGATTAAAACTCAAAGGAATTGACGGGGACCCGCACAAGCGGTGGATGATGTGGATTAATTCGATGCAACGCGAAGAACCTTACCTGGTC<br>TTGACATGTACGGAATCCTCCAGAGACGGAGGAGTGCCTTCGGGAGCCGTAACACAGGTGCTGCATGGCTGTCGTCAGCTCGTGTCGTGA<br>GATGTTGGGTTAAGTCCCGCAACGAGCGCAACCCTTGTCATTAGTTGCCATCATTTGGTTGGGCACTCTAATGAGACTGCCGGTGACAAGC<br>CGGAGGAAGGTGGGGATGACGTCAAGTCCTCATGGCCCTTATGACCAGGGCTTCACACGTCATACAATGGTCGGTACAGAGGGTAGCCAA<br>GCCGCGAGGTGGAGCCAATCCCACAAAACCGATCGTAGTCCGGATTGCACTCTGCAACTCGAGTGCATGAAGTCGGAATCGCTAGTAATC<br>GCAGGTCAGCATACTGCGGTGAATACGTTCCCGGGTCTTGTACACACCGCCCGTCACACCATGGGAGTGGGGGATACCAGAAGTAGGTAG<br>GGTAACCGCAAGGAGCCCGCTTACCACGGTATGCTTCATGACTGGGGTGAAGTCGTAACAAGGGTAACCGTAAA	99.07	*Neisseria zoodegmatis*

CTCAGATTGAACGCTGGCGGCAGGCTTAACACATGCAAGTCGAACGGTAGCAGGGAGAAAGCTTGCTTTCTTTGCTGACGAGTGGCGGAC<br>GGGTGAGTAATGCTTGGGAATCTGGCTTATGGAGGGGGATAACTGCGGGAAACTGCAGCTAATACCGCGTATTATCGGAAGATGAAAGGG<br>TGGGACCTTCGGGCCACCTGCCATAAGATGAGCCCAAGTGGGATTAGGTAGTTGGTGGGGTAAGGGCCTACCAAGCCGACGATCTCTAGC<br>TGGTCTGAGAGGATGACCAGCCACACTGGGACTGAGACACGGCCCAGACTCCTACGGGAGGCAGCAGTGGGGAATATTGCGCAATGGGG<br>GGAACCCTGACGCAGCCATGCCGCGTGAATGAAGAAGGCCTTCGGGTTGTAAAGTTCTTTCGGTAATGAGGAAGGGGTATTATTGAATAG<br>ATAATATCATTGACGTTAATTACAGAAGAAGCACCGGCTAACTCCGTGCCAGCAGCCGCGGTAATACGGAGGGTGCGAGCGTTAATCGGA<br>ATAACTGGGCGTAAAGGGCACGCAGGCGGACTTTTAAGTGAGATGTGAAATCCCCGAGCTTAACTTGGGAACTGCATTTCAGACTGGGAG<br>TCTAGAGTACTTTAGGGAGGGGTAGAATTCCACGTGTAGCGGTGAAATGCGTAGAGATGTGGAGGAATACCGAAGGCGAAGGCAGCCCCT<br>TGGGAATGTACTGACGCTCATGTGCGAAAGCGTGGGGAGCAAACAGGATTAGATACCCTGGTAGTCCACGCTGTAAACGCTGTCGATTTG<br>GGGATTGGGCTTTATGCTTGGTGCCCGAAGCTAACGTGATAAATCGACCGCCTGGGGAGTACGGCCGCAAGGTTAAAACTCAAATGAATT<br>GACGGGGGCCCGCACAAGCGGTGGAGCATGTGGTTTAATTCGATGCAACGCGAAGAACCTTACCTACTCTTGACATCCTAAGAAGAGCTC<br>AGAGATGAGCTTGTGCCTTCGGGAACTTAGAGACAGGTGCTGCATGGCTGTCGTCAGCTCGTGTTGTGAAATGTTGGGTTAAGTCCCGCAA<br>CGAGCGCAACCCTTATCCTTTGTTGCCAGCGATTTGGTCGGGAACTCAAAGGAGACTGCCAGTGACAAACTGGAGGAAGGTGGGGATGAC<br>GTCAAGTCATCATGGCCCTTACGAGTAGGGCTACACACGTGCTACAATGGTGCATACAGAGGGCGGCGAGACGGCGACGTTGAGCGAATC<br>TCATAAAGTGCATCTAAGTCCGGATTGGAGTCTGCAACTCGACTCCATGAAGTCGGAATCGCTAGTAATCGCAAATCAGAATGTTGCGGT<br>GAATACGTTCCCGGGCCTTGTACACACCGCCCGTCACACCATGGGAGTGGGTTGTACCAGAAGTGGATAGCTTAACGGAAACGAGGGCGT<br>TCACCACGGTATGATTCATGACTGGGGTG	99.66	*Pasteurella multocida subsp. multocida*
TTTTAGAGTTTGAATCATGGCTCAGGATGAACGCTGGCGGCGTGCCTAATACATGCAAGTCGAGCGAACAGATAAGGAGCTTGCTCCTTTG<br>ACGTTAGCGGCGGACGGGTGAGTAACACGTGGGTAACCTACCTATAAGACTGGAATAACTCCGGGAAACCGGGGCTAATGCCGGATAAC<br>ATGTTGAACCGCATGGTTCTACAGTGAAAGACGGTCTTGCTGTCACTTATAGATGGACCCGCGCCGTATTAGCTAGTTGGTGGGGTAACGG<br>CCTACCAAGGCGACGATACGTAGCCGACCTGAGAGGGTGATCGGCCACACTGGAACTGAGACACGGTCCAGACTCCTACGGGAGGCAGC<br>AGTAGGGAATCTTCCGCAATGGGCGAAAGCCTGACGGAGCAACGCCGCGTGAGTGATGAAGGTCTTCGGATCGTAAAGCTCTGTTGTTAG<br>GGAAGAACAAATGTGTAAGTAACTGTGCACATCTTGACGGTACCTAACCAGAAAGCCACGGCTAACTACGTGCCAGCAGCCGCGGTAATA<br>CGTAKGKGGSAAGCGTTATCCGGRATTATTGGGCGTAAAGCGCGCGTAGGCGGTTTTTTAAGTCTGATGTGAAAGCCCACGGCTCAACCGT<br>GGAGGGTCATTGGAAACTGGAAAACTTGAGTGCAGAAGAGGAAAGTGGAATTCCATGTGTAGCGGTGAAATGCGCAGAGATATGGAGGA<br>ACACCAGTGGCGAAGGCGGCTTTCTGGTCTGCAACTGACCGCTGATGTGCGAAAGSGYGGSGATCAAACAGGATTAGATACCCTGGTAGT<br>CCACGCCGTAAACGATGAGTGCTAAGTGTTAGGGGGTTTCCGCCCCTTAGTGCTGCAGCTAACGCATTAAGCACTCCGCCTGGGGAGTAC<br>GGTCGCAAGACTGAAACTCAAAGGAATTGACGGGGACCCGCACAAGCGGTGGAGCATGTGGTTTAATTCGAAGCAACGCGAAGAACCTT<br>ACCAAATCTTGACATCCTTTGACCGCTCTAGAGATAGAGTTTTCCTCTTCGGAGGACAAAGTGACAGGTGGTGCATGGTTGTCGTCAGCTC<br>GTGTCGTGAGATGTTGGGTTAAGTCCCGCAACGAGCGCAACCCTTGAACTTAGTTGCCATCATTCAGTTGGGCACTCTAAGTTGACTGCCG<br>GTGACAAACCGGAGGAAGGTGGGGATGACGTCAAATCATCATGCCCCTTATGATTTGGGCTACACACGTGCTACAATGGACAATACAAAG<br>GGCAGCGAAACCGCGAGGTCAAGCAAATCCCATAAAGTTGTTCTCAGTTCGGATTGTAGTCTGCAACTCGACTACATGAAGCTGGAATCG<br>CTAGTAATCGTAGATCAGCATGCTACGGTGAATACGTTCCCGGGTCTTGTACACACCGCCCGTCACACCACGAGAGTTTGTAACACCCGA<br>AGCCGGTGGAGTAACCATTTTGGAGCTAGCCGTCGAAGGTGGGACAAATGATTGGGGTGAAGTCGTACAGAGGGAAACCGTAAA	99.34	*Staphylococcus pseudintermedius*

CTCAGATTGAACGCTGGCGGCATGCTTTACACATGCAAGTCGGACGGCAGCACAGAGAAGCTTGCTTCTTGGGTGGCGAGTGGCGAACGG<br>GTGAGTAATGCATCGGAACGTACCGAGTAGTGGGGGATAACTGTCCGAAAGGATGGCTAATACCGCATACACTTTGAGAAGGAAAGCAG<br>GGGCTCTTCGGACCTTGCGCTATTCGAGCGGCCGATGTCTGATTAGCTAGTTGGTGGGGTAAAGGCCTACCAAGGCGACGATCAGTAGCG<br>GGTCTGAGAGGATGATCCGCCACACTGGGACTGAGACACGGCCCAGACTCCTACGGGAGGCAGCAGTGGGGAATTTTGGACAATGGGCG<br>CAAGCCTGATCCAGCCATGCCGCGTGTCTGAAGAAGGCCTTCGGGTTGTAAAGGACTTTTGTCAGGGAAGAAAAGGTTGTGGTTAATACC<br>CATGACTGATGACGGTACCTGAAGAATAAGCACCGGCTAACTACGTGCCAGCAGCCGCGGTAATACGTAGGGTGCGAGCGTTAATCGGAA<br>TTACTGGGCGTAAAGCGGGCGCAGACGGTTACTTAAGTCAGATGTGAAATCCCCGGGCTCAACCTGGGAACTGCGTTTGAAACTGGGTGA<br>CTAGAGTATGTCAGAGGGGGGTAGAATTCCACGTGTAGCAGTGAAATGCGTAGAGATGTGGAGGAATACCGATGGCGAAGGCAGCCCCCT<br>GGGATAATACTGACGTTCATGCCCGAAAGCGTGGGTAGCAAACAGGATTAGATACCCTGGTAGTCCACGCCCTAAACGATGTCAATTAGC<br>TGTTGGGCAACTTGATTGCTTAGTAGCGTAGCTAACGCGTGAAATTGACCGCCTGGGGAGTACGGTCGCAAGATTAAAACTCAAAGGAAT<br>TGACGGGGACCCGCACAAGCGGTGGATGATGTGGATTAATTCGATGCAACGCGAAGAACCTTACCTGGTCTTGACATGTACGGAATCCTC<br>CAGAGACGGAGGAGTGCCTTCGGGAGCCGTAACACAGGTGCTGCATGGCTGTCGTCAGCTCGTGTCGTGAGATGTTGGGTTAAGTCCCGC<br>AACGAGCGCAACCCTTGTCATTAGTTGCCATCATTTGGTTGGGCACTCTAATGAGACTGCCGGTGACAAGCCGGAGGAAGGTGGGGATGA<br>CGTCAAGTCCTCATGGCCCTTATGACCAGGGCTTCACACGTCATACAATGGTCGGTACAAAGGGTAGCCAAGCCGCGAGGTGGAGCCAAT<br>CCCACAAAACCGATCGTAGTCCGGATTGCACTCTGCAACTCGAGTGCATGAAGTCGGAATCGCTAGTAATCGCAGGTCAGCATACTGCGG<br>TGAATACGTTCCCGGGTCTTGTACACACCGCCCGTCACACCATGGGAGTGGGGGATACCAGAAGTAGGTAGGGTAACCGCAAGGGGCCCG<br>CTTACCACGGTATGCTTCATGACTGG	99.73	*Neisseria zoodegmatis*
TTTAGAGTTTGAATCATGGCTCAGGATGAACGCTGGCGGCGTGCCTAATACATGCAAGTCGAGCGAACAGATAAGGAGCTTGCTCCTTTG<br>ACGTTAGCGGCGGACGGGTGAGTAACACGTGGGTAACCTACCTATAAGACTGGAATAACTCCGGGAAACCGGGGCTAATGCCGGATAAC<br>ATGTTGAACCGCATGGTTCTACAGTGAAAGACGGTCTTGCTGTCACTTATAGATGGACCCGCGCCGTATTAGCTAGTTGGTGGGGTAACGG<br>CCTACCAAGGCGACGATACGTAGCCGACCTGAGAGGGTGATCGGCCACACTGGAACTGAGACACGGTCCAGACTCCTACGGGAGGCAGC<br>AGTAGGGAATCTTCCGCAATGGGCGAAAGCCTGACGGAGCAACGCCGCGTGAGTGATGAAGGTCTTCGGATCGTAAAGCTCTGTTGTTAG<br>GGAAGAACAAATGTGTAAGTAACTGTGCACATCTTGACGGTACCTAACCAGAAAGCCACGGCTAACTACGTGCCAGCAGCCGCGGTAATA<br>CGTAGGKGGSRRRCGTTAYCCGGAATTATTGGGCGTAAAGCGCGCGTAGGCGGTTTTTTAAGTCTGATGTGAAAGCCCACGGCTCAACCG<br>TGGAGGGTCATTGGAAACTGGAAAACTTGAGTGCAGAAGAGGAAAGTGGAATTCCATGTGTAGCGGTGAAATGCGCAGAGATATGGAGG<br>AACACCAGTGGCGAAGGCGGCTTTCTGGTCTGCAACTGACGCTGATGTGCGAAAGCGWRGGSMYCAAACAGGATTAGATACCCTGGTAG<br>TCCACGCCGTAAACGATGAGTGCTAAGTGTTAGGGGGTTTCCGCCCCTTAGTGCTGCAGCTAACGCATTAAGCACTCCGCCTGGGGAGTA<br>CGGTCGCAAGACTGAAACTCAAAGGAATTGACGGGGACCCGCACAAGCGGTGGAGCATGTGGTTTAATTCGAAGCAACGCGAAGAACCT<br>TACCAAATCTTGACATCCTTTGACCGCTCTAGAGATAGAGTTTTCCTCTTCGGAGGACAAAGTGACAGGTGGTGCATGGTTGTCGTCAGCT<br>CGTGTCGTGAGATGTTGGGTTAAGTCCCGCAACGAGCGCAACCCTTGAACTTAGTTGCCATCATTCAGTTGGGCACTCTAAGTTGACTGCC<br>GGTGACAAACCGGAGGAAGGTGGGGATGACGTCAAATCATCATGCCCCTTATGATTTGGGCTACACACGTGCTACAATGGACAATACAAA<br>GGGCAGCGAAACCGCGAGGTCAAGCAAATCCCATAAAGTTGTTCTCAGTTCGGATTGTAGTCTGCAACTCGACTACATGAAGCTGGAATC<br>GCTAGTAATCGTAGATCAGCATGCTACGGTGAATACGTTCCCGGGTCTTGTACACACCGCCCGTCACACCACGAGAGTTTGTAACACCCG<br>AAGCCGGTGGAGTAACCATTTTGGAGCTAGCCGTCGAAGGTGGGACAAATGATTGGGGTGAAGTCGTACAGGGGGAAACCGTAAA	99.14	*Staphylococcus pseudintermedius*
GGCTGCCTTTCGAGCTCAGCGTCAGTTACAGACCAGAGAGCCGCTTTCGCCACCGGTGTTCCTCCATATATCTACGCATTTCACCGCTACA<br>CATGGAATTCCACTCTCCCCTTCTGCACTCAAGTTCTCCAGTTTCCAAAGCATACATTGGTTGAGCCAATGCCTTTAACTTCAGACTTAAAG<br>AACCGCCTGCGCTCGCTTTACGCCCAATAAATCCGGACAACGCTCGGGACCTACGTATTACCGCGGCTGCTGGCACGTAGTTAGCCGTCC<br>CTTTCTGGTTAGTTACCGTCACATAATGGGTTTTCCACTCCCATTACCGTTCTTCTCTAACAACAGAGCTTTACGATCCGAAAACCTTCTTC<br>ACTCACGCGGCGTTGCTCGGTCAGGGTTCCCCCCATTGCCGAAGATTCCCTACTGCTGCCTCCCGTAGGAGTCTGGGCCGTGTCTCAGTCC<br>CAGTGTGGCCGATCACCCTCTCAGGTCGGCTATGTATCGTCGCCTTGGTGAGCCTTTACCTCACCAACTAGCTAATACAACGCAGGTCCAT<br>CTCATAGTGGAGCAGTTGCCCCTTTTAAGTTCTTAACATGTGTTAAGCACTTTTATGCGGTATTAGCTATCGTTTCCAATAGTTATCCCCCG<br>CTATCAGGTAGGTTACCTACGCGTTACTCACCCGTTCGCAACTCCTTAGACTAGTGCAAGCACCTGTCCTCAGCGTTCTACTTGCATGTATT<br>AGGCACGCCGCCAGCGTTCGTCCTGAGCCATATTCCAAACTCTAAAAA	99	*Streptococcus sp. (canis)*

CTCAGATTGAACGCTGGCGGCAGGCTTAACACATGCAAGTCGAACGGTAGCAGGAAGAAAGCTTGCTTTCTTTGCTGACGAGTGGCGGAC<br>GGGTGAGTAATGCTTGGGAATCTGGCTTATGGAGGGGGATAACTGTGGGAAACTGCAGCTAATACCGCGTATTCTCTGAGGAGGAAAGGG<br>TGGGACCTTAGGGCCACCTGCCATAAGATGAGCCCAAGTGGGATTAGGTAGTTGGTGGGGTAAAGGCCTACCAAGCCTGCGATCTCTAGC<br>TGGTCTGAGAGGATGACCAGCCACACTGGAACTGAGACACGGTCCAGACTCCTACGGGAGGCAGCAGTGGGGAATATTGCGCAATGGGG<br>GGAACCCTGACGCAGCCATGCCGCGTGAATGAAGAAGGCCTTCGGGTTGTAAAGTTCTTTCGGTAATGAGGAAGGGATGTTGTTAAATAG<br>ATAGCATCATTGACGTTAATTACAGAAGAAGCACCGGCTAACTCCGTGCCAGCAGCCGCGGTAATACGGAGGGTGCGAGCGTTAATCGGA<br>ATAACTGGGCGTAAAGGGCACGCAGGCGGACTTTTAAGTGAGATGTGAAATCCCCGAGCTTAACTTGGGAACTGCATTTCAGACTGGGAG<br>TCTAGAGTACTTTAGGGAGGGGTAGAATTCCACGTGTAGCGGTGAAATGCGTAGAGATGTGGAGGAATACCGAAGGCGAAGGCAGCCCCT<br>TGGGAATGTACTGACGCTCATGTGCGAAAGCGTGGGGAGCAAACAGGATTAGATACCCTGGTAGTCCACGCTGTAAACGCTGTCGATTTG<br>GGGATTGGGCTATATGCTTGGTGCCCGAAGCTAACGTGATAAATCGACCGCCTGGGGAGTACGGCCGCAAGGTTAAAACTCAAATGAATT<br>GACGGGGGCCCGCACAAGCGGTGGAGCATGTGGTTTAATTCGATGCAACGCGAAGAACCTTACCTACTCTTGACATCCTAAGAAGAGCTC<br>AGAGATGAGCTTGTGCCTTCGGGAACTTAGAGACAGGTGCTGCATGGCTGTCGTCAGCTCGTGTTGTGAAATGTTGGGTTAAGTCCCGCAA<br>CGAGCGCAACCCTTATCCTTTGTTGCCAGCGATTCGGTCGGGAACTCAAAGGAGACTGCCAGTGACAAACTGGAGGAAGGTGGGGATGAC<br>GTCAAGTCATCATGGCCCTTACGAGTAGGGCTACACACGTGCTACAATGGTGCATACAGAGGGCAGCGAGAGTGCGAGCTTGAGCGAATC<br>TCAGAAAGTGCATCTAAGTCCGGATTGGAGTCTGCAACTCGACTCCATGAAGTCGGAATCGCTAGTAATCGCAAATCAGAATGTTGCGGT<br>GAATACGTTCCCGGGCCTTGTACACACCGCCCGTCACACCATGGGAGTGGGTTGTACCAGAAGTAGATAGCTTAACCTTCGGGGGGGCGT<br>TTACCACGGTATGATTCATGACTGGGGT	100	*Pasteurella multocida*
GGCTGCCTTTCGAGCTCAGCGTCAGTTACAGACCAGAGAGCCGCTTTCGCCACCGGTGTTCCTCCATATATCTACGCATTTCACCGCTACA<br>CATGGAATTCCACTCTCCCCTTCTGCACTCAAGTTCTCCAGTTTCCAAAGCATACATTGGTTGAGCCAATGCCTTTAACTTCAGACTTAAAG<br>AACCGCCTGCGCTCGCTTTACGCCCAATAAATCCGGACAACGCTCGGGACCTACGTATTACCGCGGCTGCTGGCACGTAGTTAGCCGTCC<br>CTTTCTGGTTAGTTACCGTCACATAATGGGTTTTCCACTCCCATTACCGTTCTTCTCTAACAACAGAGCTTTACGATCCGAAAACCTTCTTC<br>ACTCACGCGGCGTTGCTCGGTCAGGGTTCCCCCATTGCCGAAGATTCCCTACTGCTGCCTCCCGTAGGAGTCTGGGCCGTGTCTCAGTCC<br>CAGTGTGGCCGATCACCCTCTCAGGTCGGCTATGTATCGTCGCCTTGGTGAGCCTTTACCTCACCAACTAGCTAATACAACGCAGGTCCAT<br>CTCATAGTGGAGCAGTTGCCCCTTTTAAGTTCTTAACATGTGTTAAGCACTTTTATGCGGTATTAGCTATCGTTTCCAATAGTTATCCCCCG<br>CTATCAGGTAGGTTACCTACGCGTTACTCACCCGTTCGCAACTCCTTAGACTAGTGCAAGCACCTGTCCTCAGCGTTCTACTTGCATGTATT<br>AGGCACGCCGCCAGCGTTCGTCCTGAGCCATATTCCAAACTCTAAAAA	99	*Streptococcus sp. (canis)*
GATTGAACGCTGGCGGCATGCTTTACACATGCAAGTCGGACGGCAGCACAGAGAAGCTTGCTTCTTGGGTGGCGAGTGGCGAACGGGTGA<br>GTAATGCATCGGAACGTACCGAGTAGTGGGGGATAACTGTCCGAAAGGATGGCTAATACCGCATACGCTTTGAGAAGGAAAGCAGGGGCT<br>CTTCGGACCTTGCGCTATTCGAGCGGCCGATGTCTGATTAGCTGGTTGGTGGGGTAAAGGCCTACCAAGGCGACGATCAGTAGTGGGTCTG<br>AGAGGATGATCCGCCACACTGGGACTGAGACACGGCCCAGACTCCTACGGGAGGCAGCAGTGGGGAATTTTGGACAATGGGCGCAAGCC<br>TGATCCAGCCATGCCGCGTGTCTGAAGAAGGCCTTCGGGTTGTAAAGGACTTTTGTCAGGGAAGAAAAGGTTGTGGTTAATACCCATGACT<br>GATGACGGTACCTGAAGAATAAGCACCGGCTAACTACGTGCCAGCAGCCGCGGTAATACGTAGGGTGCGAGCGTTAATCGGAATTACTGG<br>GCGTAAAGCGGGCGCAGACGGTTACTTAAGTCAGATGTGAAATCCCCGGGCTCAACCTGGGAACTGCGTTTGAAACTGGGTGACTAGAGT<br>ATGTCAGAGGGGGGTAGAATTCCACGTGTAGCAGTGAAATGCGTAGAGATGTGGAGGAATACCGATGGCGAAGGCAGCCCCCTGGGATA<br>ATACTGACGTTCATGCCCGAAAGCGTGGGTAGCAAACAGGATTAGATACCCTGGTAGTCCACGCCCTAAACGATGTCAATTAGCTGTTGG<br>GCAACTTGATTGCTTAGTAGCGTAGCTAACGCGTGAAATTGACCGCCTGGGGAGTACGGTCGCAAGATTAAAACTCAAAGGAATTGACGG<br>GGACCCGCACAAGCGGTGGATGATGTGGATTAATTCGATGCAACGCGAAGAACCTTACCTGGTCTTGACATGTACGGAATCCTCCAGAGA<br>CGGAGGAGTGCCTTCGGGAGCCGTAACACAGGTGCTGCATGGCTGTCGTCAGCTCGTGTCGTGAGATGTTGGGTTAAGTCCCGCAACGAG<br>CGCAACCCTTGTCATTAGTTGCCATCATTTGGTTGGGCACTCTAATGAGACTGCCGGTGACAAGCCGGAGGAAGGTGGGGATGACGTCAA<br>GTCCTCATGGCCCTTATGACCAGGGCTTCACACGTCATACAATGGTCGGTACAGAGGGTAGCCAAGCCGCGAGGTGGAGCCAATCCCACA<br>AAACCGATCGTAGTCCGGATTGCACTCTGCAACTCGAGTGCATGAAGTCGGAATCGCTAGTAATCGCAGGTCAGCATACTGCGGTGAATA<br>CGTTCCCGGGTCTTGTACACACCGCCCGTCACACCATGGGAGTGGGGGATACCAGAAGTAGGTAGGGTAACCGCAAGGAGCCCGCTTACC<br>ACGGTATGCTTCATGACTGGGGT	99.93	*Neisseria zoodegmatis*

GCTCAGGATGAACGCTGGCGGCGTGCCTAATACATGCAAGTCGAGCGAACAGATAAGGAGCTTGCTCCTTTGACGTTAGCGGCGGACGGG<br>TGAGTAACACGTGGGTAACCTACCTATAAGACTGGAATAACTCCGGGAAACCGGGGCTAATGCCGGATAACATTTAGAACCGCATGGTTC<br>TAAAGTGAAAGATGGTTTTGCTATCACTTATAGATGGACCCGCGCCGTATTAGCTAGTTGGTAAGGTAACGGCTTACCAAGGCAACGATAC<br>GTAGCCGACCTGAGAGGGTGATCGGCCACACTGGAACTGAGACACGGTCCAGACTCCTACGGGAGGCAGCAGTAGGGAATCTTCCGCAA<br>TGGGCGAAAGCCTGACGGAGCAACGCCGCGTGAGTGATGAAGGTCTTCGGATCGTAAAACTCTGTTATTAGGGAAGAACAAATGTGTAAG<br>TAACTGTGCACGTCTTGACGGTACCTAATCAGAAAGCCACGGCTAACTACGTGCCAGCAGCCGCGGTAATACGTAGGTGGCAAGCGTTAT<br>CCGGAATTATTGGGCGTAAAGCGCGCGTAGGCGGTTTCTTAAGTCTGATGTGAAAGCCCACGGCTCAACCGTGGAGGGTCATTGGAAACT<br>GGGAAACTTGAGTGCAGAAGAGGAAAGTGGAATTCCATGTGTAGCGGTGAAATGCGCAGAGATATGGAGGAACACCAGTGGCGAAGGCG<br>ACTTTCTGGTCTGTAACTGACGCTGATGTGCGAAAGCGTGGGGATCAAACAGGATTAGATACCCTGGTAGTCCACGCCGTAAACGATGAG<br>TGCTAAGTGTTAGGGGGTTTCCGCCCCTTAGTGCTGCAGCTAACGCATTAAGCACTCCGCCTGGGGAGTACGACCGCAAGGTTGAAACTC<br>AAAGGAATTGACGGGGACCCGCACAAGCGGTGGAGCATGTGGTTTAATTCGAAGCAACGCGAAGAACCTTACCAAATCTTGACATCCTTT<br>GACAACTCTAGAGATAGAGCCTTCCCCTTCGGGGGACAAAGTGACAGGTGGTGCATGGTTGTCGTCAGCTCGTGTCGTGAGATGTTGGGTT<br>AAGTCCCGCAACGAGCGCAACCCTTAAGCTTAGTTGCCAGCATTAAGTTGGGCACTCTAAGTTGACTGCCGGTGACAAACCGGAGGAAGG<br>TGGGGATGACGTCAAATCATCATGCCCCTTATGATTTGGGCTACACACGTGCTACAATGGACAATACAAAGGGCAGCTAAACCGCGAGGT<br>CATGCAAATCCCATAAAGTTGTTCTCAGTTCGGATTGTAGTCTGCAACTCGACTACATGAAGCTGGAATCGCTAGTAATCGTAGATCAGCA<br>TGCTACGGTGAATACGTTCCCGGGTCTTGTACACACCGCCCGTCACACCACGAGAGTTTGTAACACCCGAAGCCGGTGGAGTAACCATCT<br>ATGGAGCTAGCCGTCGAAGGTGGGACAAATGATTGGGGTGAAGT	100	*Staphylococcus cohnii*
CTCAGATTGAACGCTGGCGGCATGCTTTACACATGCAAGTCGGACGGCAGCACAGAGAAGCTTGCTTCTTGGGTGGCGAGTGGCGAACGG<br>GTGAGTAATGCATCGGAACGTACCGAGTAGTGGGGGATAACTGTCCGAAAGGATGGCTAATACCGCATACGCTTTGAGAAGGAAAGCAG<br>GGGCTCTTCGGACCTTGCGCTATTCGAGCGGCCGATGTCTGATTAGCTGGTTGGTGGGGTAAAGGCCTACCAAGGCGACGATCAGTAGCG<br>GGTCTGAGAGGATGATCCGCCACACTGGGACTGAGACACGGCCCAGACTCCTACGGGAGGCAGCAGTGGGGAATTTTGGACAATGGGCG<br>CAAGCCTGATCCAGCCATGCCGCGTGTCTGAAGAAGGCCTTCGGGTTGTAAAGGACTTTTGTCAGGGAAGAAAAGGTTGTGGTTAATACC<br>CATGACTGATGACGGTACCTGAAGAATAAGCACCGGCTAACTACGTGCCAGCAGCCGCGGTAATACGTAGGGTGCGAGCGTTAATCGGAA<br>TTACTGGGCGTAAAGCGGGCGCAGACGGTTACTTAAGTCAGATGTGAAATCCCCGGGCTCAACCTGGGAACTGCGTTTGAAACTGGGTGA<br>CTAGAGTATGTCAGAGGGGGGTAGAATTCCACGTGTAGCAGTGAAATGCGTAGAGATGTGGAGGAATACCGATGGCGAAGGCAGCCCCCT<br>GGGATAATACTGACGTTCATGCCCGAAAGCGTGGGTAGCAAACAGGATTAGATACCCTGGTAGTCCACGCCCTAAACGATGTCAATTAGC<br>TGTTGGGCAACTTGATTGCTTAGTAGCGTAGCTAACGCGTGAAATTGACCGCCTGGGGAGTACGGTCGCAAGATTAAAACTCAAAGGAAT<br>TGACGGGGACCCGCACAAGCGGTGGATGATGTGGATTAATTCGATGCAACGCGAAGAACCTTACCTGGTCTTGACATGTACGGAATCCTC<br>CAGAGACGGAGGAGTGCCTTCGGGAGCCGTAACACAGGTGCTGCATGGCTGTCGTCAGCTCGTGTCGTGAGATGTTGGGTTAAGTCCCGC<br>AACGAGCGCAACCCTTGTCATTAGTTGCCATCATTTGGTTGGGCACTCTAATGAGACTGCCGGTGACAAGCCGGAGGAAGGTGGGGATGA<br>CGTCAAGTCCTCATGGCCCTTATGACCAGGGCTTCACACGTCATACAATGGTCGGTACAGAGGGTAGCCAAGCCGCGAGGTGGAGCCAAT<br>CCCACAAAACCGATCGTAGTCCGGATTGCACTCTGCAACTCGAGTGCATGAAGTCGGAATCGCTAGTAATCGCAGGTCAGCATACTGCGG<br>TGAATACGTTCCCGGGTCTTGTACACACCGCCCGTCACACCATGGGAGTGGGGGATACCAGAAGTAGGTAGGGTAACCGCAAGGAGCCCG<br>CTTACCACGGTATGCTTCATGACTGGGGTGAAGTCGTAC	100	*Neisseria zoodegmatis*

GGCTCAGATTGAACGCTGGCGGCATGCTTTACACATGCAAGTCGGACGGCAGCACAGAGAAGCTTGCTTCTTGGGTGGCGAGTGGCGAAC<br>GGGTGAGTAATGCATCGGAACGTACCGAGTAGTGGGGGATAACTGTCCGAAAGGATGGCTAATACCGCATACGCTTTGAGAAGGAAAGC<br>AGGGGCTCTTCGGACCTTGCGCTATTCGAGCGGCCGATGTCTGATTAGCTGGTTGGTGGGGTAAAGGCCTACCAAGGCGACGATCAGTAG<br>CGGGTCTGAGAGGATGATCCGCCACACTGGGACTGAGACACGGCCCAGACTCCTACGGGAGGCAGCAGTGGGGAATTTTGGACAATGGG<br>CGCAAGCCTGATCCAGCCATGCCGCGTGTCTGAAGAAGGCCTTCGGGTTGTAAAGGACTTTTGTCAGGGAAGAAAAGGTTGTGGTTAATA<br>CCCATGACTGATGACGGTACCTGAAGAATAAGCACCGGCTAACTACGTGCCAGCAGCCGCGGTAATACGTAGGGTGCGAGCGTTAATCGG<br>AATTACTGGGCGTAAAGCGGGCGCAGACGGTTACTTAAGTCAGATGTGAAATCCCCGGGCTCAACCTGGGAACTGCGTTTGAAACTGGGT<br>GACTAGAGTATGTCAGAGGGGGGTAGAATTCCACGTGTAGCAGTGAAATGCGTAGAGATGTGGAGGAATACCGATGGCGAAGGCAGCCC<br>CCTGGGATAATACTGACGTTCATGCCCGAAAGCGTGGGTAGCAAACAGGATTAGATACCCTGGTAGTCCACGCCCTAAACGATGTCAATT<br>AGCTGTTGGGCAACTTGATTGCTTAGTAGCGTAGCTAACGCGTGAAATTGACCGCCTGGGGAGTACGGTCGCAAGATTAAAACTCAAAGG<br>AATTGACGGGGACCCGCACAAGCGGTGGATGATGTGGATTAATTCGATGCAACGCGAAGAACCTTACCTGGTCTTGACATGTACGGAATC<br>CTCCAGAGACGGAGGAGTGCCTTCGGGAGCTGTAACACAGGTGCTGCATGGCTGTCGTCAGCTCGTGTCGTGAGATGTTGGGTTAAGTCC<br>CGCAACGAGCGCAACCCTTGTCATTAGTTGCCATCATTTGGTTGGGCACTCTAATGAGACTGCCGGTGACAAGCCGGAGGAAGGTGGGGA<br>TGACGTCAAGTCCTCATGGCCCTTATGACCAGGGCTTCACACGTCATACAATGGTCGGTACAGAGGGTAGCCAAGCCGCGAGGTGGAGCC<br>AATCCCACAAAACCGATCGTAGTCCGGATTGCACTCTGCAACTCGAGTGCATGAAGTCGGAATCGCTAGTAATCGCAGGTCAGCATACTG<br>CGGTGAATACGTTCCCGGGTCTTGTACACACCGCCCGTCACACCATGGGAGTGGGGGATACCAGAAGTAGGTAGGGTAACCGCAAGGAGC<br>CCGCTTACCACGGTATGCTTCATGACTGGGGTG	99.93	*Neisseria zoodegmatis*
CTCAGATTGAACGCTGGCGGCAGGCCTAACACATGCAAGTCGAGCGGTAACAGGGGAAGCTTGCTTCTCGCTGACGAGCGGCGGACGGGT<br>GAGTAATGTATGGGGATCTGCCCGATAGAGGGGGATAACTACTGGAAACGGTGGCTAATACCGCATAATCTCTTAGGAGCAAAGCAGGGG<br>ACCTTCGGGCCTTGCGCTGTCGGATGAACCCATATGGGATTAGCTAGTAGGTAAGGTAATGGCTTACCTAGGCGACGATCCCTAGCTGGTC<br>TGAGAGGATGATCAGCCACACTGGGACTGAGACACGGCCCAGACTCCTACGGGAGGCAGCAGTGGGGAATATTGCACAATGGGCGCAAG<br>CCTGATGCAGCCATGCCGCGTGTATGAAGAAGGCCCTAGGGTTGTAAAGTACTTTCAGTCGGGAGGAAGGCGTTGATGTTAATACCATCA<br>ACGATTGACGTTACCGACAGAAGAAGCACCGGCTAACTCCGTGCCAGCAGCCGCGGTAATACGGAGGGTGCAAGCGTTAATCGGAATTAC<br>TGGGCGTAAAGCGCACGCAGGCGGTTAATTAAGTTAGATGTGAAATCCCCGGGCTTAACCTGGGAATGGCATCTAAGACTGGTTAGCTAG<br>AGTCTTGTAGAGGGGGGTAGAATTCCATGTGTAGCGGTGAAATGCGTAGAGATGTGGAGGAATACCGGTGGCGAAGGCGGCCCCCTGGAC<br>AAAGACTGACGCTCAGGTGCGAAAGCGTGGGGAGCAAACAGGATTAGATACCCTGGTAGTCCACGCTGTAAACGATGTCGATTTGGAGGT<br>TGTTCCCTAGAGGAGTGGCTTCCGGAGCTAACGCGTTAAATCGACCGCCTGGGGAGTACGGCCGCAAGGTTAAAACTCAAATGAATTGAC<br>GGGGGCCCGCACAAGCGGTGGAGCATGTGGTTTAATTCGATGCAACGCGAAGAACCTTACCTACTCTTGACATCCAGAGAATTGAGCAGA<br>GATGCTGAAGTGCCTTCGGGAGCTCTGAGACAGGTGCTGCATGGCTGTCGTCAGCTCGTGTTGTGAAATGTTGGGTTAAGTCCCGCAACGA<br>GCGCAACCCTTATCCTTTGTTGCCAGCGATTCGGTCGGGAACTCAAAGGAGACTGCCGGTGATAAACCGGAGGAAGGTGGGGATGACGTC<br>AAGTCATCATGGCCCTTACGAGTAGGGCTACACACGTGCTACAATGGCGTATACAAAGAGAAGCGACCTCGCGAGAGCAAGCGGAACTC<br>ATAAAGTACGTCGTAGTCCGGATTGGAGTCTGCAACTCGACTCCATGAAGTCGGAATCGCTAGTAATCGTAGATCAGAATGCTACGGTGA<br>ATACGTTCCCGGGCCTTGTACACACCGCCCGTCACACCATGGGAGTGGGTTGCAAAAGAAGTAGGTAGCTTAACCTTCGGGAGGGCGCTT<br>ACCACTTTGTGATCCATGACTGGGGTG	100	*Providencia stuartii*
