# Supplementary material for: Isolation of Oral Bacteria, Measurement of the C-Reactive Protein, and Blood Clinical Parameters in Dogs with Oral Tumor
Source: Vet Med Int. 2023 Mar 22;2023:2582774. doi: 10.1155/2023/2582774 (PMC10060073; doi:10.1155/2023/2582774)
Supplement: Supplementary Materials — Supplementary file 1: the nucleotide sequences of bacterial isolates from the no oral mass dogs. Supplementary file 2: the nucleotide sequences of bacterial isolates from the dogs with oral mass. [file 2582774.f1.zip › 2. Supplementary file 2-The nucleotide sequences of bacterial isolates from the dogs with oral mass (1).pdf]

The nucleotide sequences of bacterial isolates from the dogs with oral mass

Sequence	% Identical	Bacterial identification
GGCTCAGATTGAACGCTGGCGGCATGCTTTACACATGCAAGTCGGACGGCAGCACAGAGAAGCTTGCTTCTTGGG<br>TGGCGAGTGGCGAACGGGTGAGTAATGCATCGGAACGTACCGAGTAGTGGGGGATAACTGTCCGAAAGGATGGC<br>TAATACCGCATACGCTTTGAGAAGGAAAGCAGGGGCTCTTCGGACCTTGCGCTATTCGAGCGGCCGATGTCTGAT<br>TAGCTGGTTGGTGGGGTAAAGGCCTACCAAGGCGACGATCAGTAGCGGGTCTGAGAGGATGATCCGCCACACTGG<br>GACTGAGACACGGCCCAGACTCCTACGGGAGGCAGCAGTGGGGAATTTTGGACAATGGGCGCAAGCCTGATCCA<br>GCCATGCCGCGTGTCTGAAGAAGGCCTTCGGGTTGTAAAGGACTTTTGTCAGGGAAGAAAAGGTTGTGGTTAATA<br>CCCATGACTGATGACGGTACCTGAAGAATAAGCACCGGCTAACTACGTGCCAGCAGCCGCGGTAATACGTAGGGT<br>GCGAGCGTTAATCGGAATTACTGGGCGTAAAGCGGGCGCAGACGGTTACTTAAGTCAGATGTGAAATCCCCGGGC<br>TCAACCTGGGAACTGCGTTTGAAACTGGGTGACTAGAGTATGTCAGAGGGGGGTAGAATTCCACGTGTAGCAGTG<br>AAATGCGTAGAGATGTGGAGGAATACCGATGGCGAAGGCAGCCCCCTGGGATAATACTGACGTTCATGCCCGAA<br>AGCGTGGGTAGCAAACAGGATTAGATACCCTGGTAGTCCACGCCCTAAACGATGTCAATTAGCTGTTGGGCAACT<br>TGATTGCTTAGTAGCGTAGCTAACGCGTGAAATTGACCGCCTGGGGAGTACGGTCGCAAGATTAAAACTCAAAGG<br>AATTGACGGGGACCCGCACAAGCGGTGGATGATGTGGATTAATTCGATGCAACGCGAAGAACCTTACCTGGTCTT<br>GACATGTACGGAATCCTCCAGAGACGGAGGAGTGCCTTCGGGAGCCGTAACACAGGTGCTGCATGGCTGTCGTCA<br>GCTCGTGTCGTGAGATGTTGGGTTAAGTCCCGCAACGAGCGCAACCCTTGTCATTAGTTGCCATCATTTGGTTGGG<br>CACTCTAATGAGACTGCCGGTGACAAGCCGGAGGAAGGTGGGGATGACGTCAAGTCCTCATGGCCCTTATGACCA<br>GGGCTTCACACGTCATACAATGGTCGGTACAGAGGGTAGCCAAGCCGCGAGGTGGAGCCAATCCCACAAAACCG<br>ATCGTAGTCCGGATTGCACTCTGCAACTCGAGTGCATGAAGTCGGAATCGCTAGTAATCGCAGGTCAGCATACTG<br>CGGTGAATACGTTCCCGGGTCTTGTACACACCGCCCGTCACACCATGGGAGTGGGGGATACCAGAAGTAGGTAGG	100	*Neisseria zoodegmatis*
CTCAGGACGAACGCTGGCGGCGTGCCTAATACATGCAAGTCGAACGCTTCTTTCCTCCCGAGTGCTTGCACTCAAT<br>TGGAAAGAGGAGTGGCGGACGGGTGAGTAACACGTGGGTAACCTACCCATCAGAGGGGGATAACACTTGGAAAC<br>AGGTGCTAATACCGCATAACAGTTTATGCCGCATGGCATAAGAGTGAAAGGCGCTTTCGGGTGTCGCTGATGGAT<br>GGACCCGCGGTGCATTAGCTAGTTGGTGAGGTAACGGCTCACCAAGGCCACGATGCATAGCCGACCTGAGAGGGT<br>GATCGGCCACACTGGGACTGAGACACGGCCCAGACTCCTACGGGAGGCAGCAGTAGGGAATCTTCGGCAATGGA<br>CGAAAGTCTGACCGAGCAACGCCGCGTGAGTGAAGAAGGTTTTCGGATCGTAAAACTCTGTTGTTAGAGAAGAAC<br>AAGGACGTTAGTAACTGAACGTCCCCTGACGGTATCTAACCAGAAAGCCACGGCTAACTACGTGCCAGCAGCCGC<br>GGTAATACGTAGGTGGCAAGCGTTGTCCGGATTTATTGGGCGTAAAGCGAGCGCAGGCGGTTTCTTAAGTCTGAT<br>GTGAAAGCCCCCGGCTCAACCGGGGAGGGTCATTGGAAACTGGGAGACTTGAGTGCAGAAGAGGAGAGTGGAAT<br>TCCATGTGTAGCGGTGAAATGCGTAGATATATGGAGGAACACCAGTGGCGAAGGCGGCTCTCTGGTCTGTAACTG<br>ACGCTGAGGCTCGAAAGCGTGGGGAGCAAACAGGATTAGATACCCTGGTAGTCCACGCCGTAAACGATGAGTGC<br>TAAGTGTTGGAGGGTTTCCGCCCTTCAGTGCTGCAGCAAACGCATTAAGCACTCCGCCTGGGGAGTACGACCGCA<br>AGGTTGAAACTCAAAGGAATTGACGGGGGCCCGCACAAGCGGTGGAGCATGTGGTTTAATTCGAAGCAACGCGA<br>AGAACCTTACCAGGTCTTGACATCCTTTGACCACTCTAGAGATAGAGCTTTCCCTTCGGGGACAAAGTGACAGGT<br>GGTGCATGGTTGTCGTCAGCTCGTGTCGTGAGATGTTGGGTTAAGTCCCGCAACGAGCGCAACCCTTATTGTTAGT<br>TGCCATCATTTAGTTGGGCACTCTAGCGAGACTGCCGGTGACAAACCGGAGGAAGGTGGGGATGACGTCAAATCA<br>TCATGCCCCTTATGACCTGGGCTACACACGTGCTACAATGGGAAGTACAACGAGTCGCTAGACCGCGAGGTCATG<br>CAAATCTCTTAAAGCTTCTCTCAGTTCGGATTGCAGGCTGCAACTCGCCTGCATGAAGCCGGAATCGCTAGTAATC<br>GCGGATCAGCACGCCGCGGTGAATACGTTCCCGGGCCTTGTACACACCGCCCGTCACACCACGAGAGTTTGTAAC<br>ACCCGAAGTCGGTGAGGTAACCTTTTTGGAGCCAGCCGCCTAAGGTGGGATAGATGATTGGGGTGAA	100	*Enterococcus faecalis*

GCACGCAGGCGGACTTTTAGGCTCAGATGTGAACGCTSSCSGCAGGCTTAACACATGCAAGTCGAACGGTAGACA<br>GGAAGAAAGCTTGCTTTCTTTGCTGACGAGTAGGATCGGACGGGTAGCGGAGTAATGCTTGGGAATCTGGAGGCT<br>TACTGGAGGGGGAGGTAACTGCTTGGGAATGTACTGCAGCTAATACCGCGTAATCTCTGAGGAGCTAACAGGAGT<br>AGGGACCCTGATTGGCCACCTGCCATAAGATGCAGCCCAAGTGGGGATTAGGCTAGTTGGTGGGGTAAAGGCCGT<br>ACCAAGCCTGCGATCTCTAGCTGGTCTGGAAGGACTGACGCAAGGTCCACACTGGAACTGAATTGACACGGTCCA<br>GACTCCTACGGGAGGCAGCATGTGGTGGKKAATATTGCTGCAATGGGGGGAACCTTACCTAGACTTGACAGCCAG<br>TGCCGCGTGCAATGATGAGAATGTGCCTTCGGGTTGTAAAGTYTCTTTCGGTAGATGAGGAAGGGATGTTGTTAAA<br>TCGAGATGTGCATCATTGACGTTAATTACAGCAACGAGAGCACCGGCTAACCTCCGTTGCCAGCGATTAGCCGTC<br>GGTAACTCAACGGAGGGTGCGAGCGTTAATCTGGAATAAGCTGGGGATGACGTAAAGTCATCATGGCCCTTACGA<br>GCAGGGCTACACACGGAKRCTTTTAAGGTGCATACAGAGTGCTGAGAATCCCCGAGCTTGAGCGAATCTTGGGAA<br>TTGCATTTCAGTACGGATGGGAGTCTGAGAGTCGACTTTAGGGAGGGGTAGAATTCCACGTGTAGCGGTGAAATG<br>CGTAGAGATGTTGGAGTGAATACGTTCGAAGGCCTTGAAGGCAGCGCCCGTCACACCATGGGAGTGGGTTGTACC<br>AGAAGTAGATAGCTTAACCTTCGGGAGGGCGTTTACCACGGTATGATTCATGACTGGGGTGAAGTCGT		*Pasteurella multocida*
CTCAGATTGAACGCTGGCGGCATGCTTTACACATGCAAGTCGGACGGCAGCACAAAGAAGCTTGCTTCTTGGGTG<br>GCGAGTGGCGAACGGGTGAGTAACGCATCGGAACGTACCGAGTAGTGGGGGATAACTGTCCGAAAGGATGGCTA<br>ATACCGCATACGCTTTGAGAAGGAAAGCGGGGGATCTTCGGACCTCGCGCTATTCGAGCGGCCGATGTCTGATTA<br>GCTGGTTGGTGGGGTAAAGGCCTACCAAGGCGACGATCAGTAGCGGGTCTGAGAGGATGATCCGCCACACTGGG<br>ACTGAGACACGGCCCAGACTCCTACGGGAGGCAGCAGTGGGGAATTTTGGACAATGGGCGCAAGCCTGATCCAG<br>CCATGCCGCGTGTCTGAAGAAGGCCTTCGGGTTGTAAAGGACTTTTGTCAGGGAAGAAAAGCTTGAGGCTAATAC<br>CCTTGAGTGATGACGGTACCTGAAGAATAAGCACCGGCTAACTACGTGCCAGCAGCCGCGGTAATACGTAGGGTG<br>CGAGCGTTAATCGGAATTACTGGGCGTAAAGCGGGCGCAGACGGTTACTTAAGTCAGATGTGAAATCCCCGGGCT<br>CAACCTGGGAACTGCGTTTGAAACTGGGTAGCTAGAGTATGTCAGAGGGGGGTAGAATTCCACGTGTAGCAGTGA<br>AATGCGTAGAGATGTGGAGGAATACCGATGGCGAAGGCAGCCCCCTGGGATAATACTGACGTTCATGCCCGAAA<br>GCGTGGGTAGCAAACAGGATTAGATACCCTGGTAGTCCACGCCCTAAACGATGTCAATTAGCTGTTGGGGCACTT<br>GATGCCTTAGTAGCGTAGCTAACGCGTGAAATTGACCGCCTGGGGAGTACGGTCGCAAGATTAAAACTCAAAGGA<br>ATTGACGGGGACCCGCACAAGCGGTGGATGATGTGGATTAATTCGATGCAACGCGAAGAACCTTACCTGGTCTTG<br>ACATGTACGGAATCCTCCAGAGACGGAGGAGTGCCTTCGGGAGCCGTAACACAGGTGCTGCATGGCTGTCGTCAG<br>CTCGTGTCGTGAGATGTTGGGTTAAGTCCCGCAACGAGCGCAACCCTTGTCATTAGTTGCCATCATTAAGTTGGGC<br>ACTCTAATGAGACTGCCGGTGACAAGCCGGAGGAAGGTGGGGATGACGTCAAGTCCTCATGGCCCTTATGACCAG<br>GGCTTCACACGTCATACAATGGTCGGTACAGAGGGTAGCCAAGCCGCGAGGTGGAGCCAATCCCACAAAACCGA<br>TCGTAGTCCGGATTGCACTCTGCAACTCGAGTGCATGAAGTCGGAATCGCTAGTAATCGCAGGTCAGCATACTGC<br>GGTGAATACGTTCCCGGGTCTTGTACACACCGCCCGTCACACCATGGGAGTGGGGGATACCAGAAGTAGGTAGGG	100	*Neisseria dumasiana*

CTCAGATTGAACGCTGGCGGCAGGCCTAACACATGCAAGTCGAACGGTAACAGGAAGCAGCTTGCTTCTTTGCTG<br>ACGAGTGGCGGACGGGTGAGTAATGTCTGGGAAACTGCCTGATGGAGGGGGATAACTACTGGAAACGGTAGCTA<br>ATACCGCATAACGTCGCAAGACCAAAGAGGGGGACCTTCGGGCCTCTTGCCATCGGATGTGCCCAGATGGGATTA<br>GCTAGTAGGTGGGGTAACGGCTCACCTAGGCGACGATCCCTAGCTGGTCTGAGAGGATGACCAGCCACACTGGAA<br>CTGAGACACGGTCCAGACTCCTACGGGAGGCAGCAGTGGGGAATATTGCACAATGGGCGCAAGCCTGATGCAGC<br>CATGCCGCGTGTATGAAGAAGGCCTTCGGGTTGTAAAGTACTTTCAGCGGGGAGGAAGGGAGTAAAGTTAATACC<br>TTTGCTCATTGACGTTACCCGCAGAAGAAGCACCGGCTAACTCCGTGCCAGCAGCCGCGGTAATACGGAGGGTGC<br>AAGCGTTAATCGGAATTACTGGGCGTAAAGCGCACGCAGGCGGTTTGTTAAGTCAGATGTGAAATCCCCGGGCTC<br>AACCTGGGAACTGCATCTGATACTGGCAAGCTTGAGTCTCGTAGAGGGGGGTAGAATTCCAGGTGTAGCGGTGAA<br>ATGCGTAGAGATCTGGAGGAATACCGGTGGCGAAGGCGGCCCCCTGGACGAAGACTGACGCTCAGGTGCGAAAG<br>CGTGGGGAGCAAACAGGATTAGATACCCTGGTAGTCCACGCCGTAAACGATGTCGACTTGGAGGTTGTGCCCTTG<br>AGGCGTGGCTTCCGGAGCTAACGCGTTAAGTCGACCGCCTGGGGAGTACGGCCGCAAGGTTAAAACTCAAATGAA<br>TTGACGGGGGCCCGCACAAGCGGTGGAGCATGTGGTTTAATTCGATGCAACGCGAAGAACCTTACCTGGTCTTGA<br>CATCCACGGAAGTTTTCAGAGATGAAAAGGTGCCTTCGGGAACCGTGAGACAGGTGCTGCATGGCTGTCGTCAGC<br>TCGTGTTGTGAAATGTTGGGTTAAGTCCCGCAACGAGCGCAACCCTTATCCTTTGTTGCCAGCGGTCCGGCCGGGA<br>ACTCAAAGGAGACTGCCAGTGATAAACTGGAGGAAGGTGGGGATGACGTCAAGTCATCATGGCCCTTACGACCA<br>GGGCTACACACGTGCTACAATGGCGCATACAAAGAGAAGCGACCTCGCGAGAGCAAGCGGACCTCATAAAGTGC<br>GTCGTAGTCCGGATTGGAGTCTGCAACTCGACTCCATGAAGTCGGAATCGCTAGTAATCGTGGATCAGAATGCCA<br>CGGTGAATACGTTCCCGGGCCTTGTACACACCGCCCGTCACACCATGGGAGTGGGTTGCAAAAGAAGTAGGTAGC	99.86	*Escherichia coli*
CTCAGATTGAACGCTGGCGGCAGGCCTAACACATGCAAGTCGAGCGGTAGCACAGAGAGCTTGCTCTCGGGTGAC<br>GAGCGGCGGACGGGTGAGTAATGTCTGGGAAACTGCCTGATGGAGGGGGATAACTACTGGAAACGGTAGCTAAT<br>ACCGCATAATGTCGCAAGACCAAAGTGGGGGACCTTCGGGCCTCATGCCATCAGATGTGCCCAGATGGGATTAGC<br>TAGTAGGTGGGGTAACGGCTCACCTAGGCGACGATCCCTAGCTGGTCTGAGAGGATGACCAGCCACACTGGAACT<br>GAGACACGGTCCAGACTCCTACGGGAGGCAGCAGTGGGGAATATTGCACAATGGGCGCAAGCCTGATGCAGCCA<br>TGCCGCGTGTGTGAAGAAGGCCTTCGGGTTGTAAAGCACTTTCAGCGGGGAGGAAGGCGATAAGGTTAATAACCT<br>TGGCGATTGACGTTACCCGCAGAAGAAGCACCGGCTAACTCCGTGCCAGCAGCCGCGGTAATACGGAGGGTGCA<br>AGCGTTAATCGGAATTACTGGGCGTAAAGCGCACGCAGGCGGTCTGTCAAGTCGGATGTGAAATCCCCGGGCTCA<br>ACCTGGGAACTGCATTCGAAACTGGCAGGCTAGAGTCTTGTAGAGGGGGGTAGAATTCCAGGTGTAGCGGTGAAA<br>TGCGTAGAGATCTGGAGGAATACCGGTGGCGAAGGCGGCCCCCTGGACAAAGACTGACGCTCAGGTGCGAAAGC<br>GTGGGGAGCAAACAGGATTAGATACCCTGGTAGTCCACGCCGTAAACGATGTCGATTTGGAGGTTGTGCCCTTGA<br>GGCGTGGCTTCCGGAGCTAACGCGTTAAATCGACCGCCTGGGGAGTACGGCCGCAAGGTTAAAACTCAAATGAAT<br>TGACGGGGGCCCGCACAAGCGGTGGAGCATGTGGTTTAATTCGATGCAACGCGAAGAACCTTACCTGGTCTTGAC<br>ATCCACAGAACTTTCCAGAGATGGATTGGTGCCTTCGGGAACTGTGAGACAGGTGCTGCATGGCTGTCGTCAGCT<br>CGTGTTGTGAAATGTTGGGTTAAGTCCCGCAACGAGCGCAACCCTTATCCTTTGTTGCCAGCGGTTCGGCCGGGAA<br>CTCAAAGGAGACTGCCAGTGATAAACTGGAGGAAGGTGGGGATGACGTCAAGTCATCATGGCCCTTACGACCAG<br>GGCTACACACGTGCTACAATGGCATATACAAAGAGAAGCGACCTCGCGAGAGCAAGCGGACCTCATAAAGTATG<br>TCGTAGTCCGGATTGGAGTCTGCAACTCGACTCCATGAAGTCGGAATCGCTAGTAATCGTAGATCAGAATGCTAC<br>GGTGAATACGTTCCCGGGCCTTGTACACACCGCCCGTCACACCATGGGAGTGGGTTGCAAAAGAAGTAGGTAGCT	100	*Klebsiella pneumoniae*

GGCTCAGGATGAACGCTGGCGGCGTGCCTAATACATGCAAGTCGAGCGAACAGATAAGGAGCTTGCTCCTTTGAC<br>GTTAGCGGCGGACGGGTGAGTAACACGTGGGTAACCTACCTATAAGACTGGAATAACTCCGGGAAACCGGGGCT<br>AATGCCGGATAACATGTTGAACCGCATGGTTCTACAGTGAAAGACGGTCTTGCTGTCACTTATAGATGGACCCGC<br>GCCGTATTAGCTAGTTGGTGGGGTAACGGCCTACCAAGGCGACGATACGTAGCCGACCTGAGAGGGTGATCGGCC<br>ACACTGGAACTGAGACACGGTCCAGACTCCTACGGGAGGCAGCAGTAGGGAATCTTCCGCAATGGGCGAAAGCC<br>TGACGGAGCAACGCCGCGTGAGTGATGAAGGTCTTCGGATCGTAAAGCTCTGTTGTTAGGGAAGAACAAATGTGT<br>AAGTAACTGTGCACATCTTGACGGTACCTAACCAGAAAGCCACGGCTAACTACGTGCCAGCAGCCGCGGTAATAC<br>GTAGGTGGCAAGCGTTATCCGGAATTATTGGGCGTAAAGCGCGCGTAGGCGGTTTTTTAAGTCTGATGTGAAAGC<br>CCACGGCTCAACCGTGGAGGGTCATTGGAAACTGGAAAACTTGAGTGCAGAAGAGGAAAGTGGAATTCCATGTGT<br>AGCGGTGAAATGCGCAGAGATATGGAGGAACACCAGTGGCGAAGGCGGCTTTCTGGTCTGCAACTGACGCTGATG<br>TGCGAAAGCGTGGGGATCAAACAGGATTAGATACCCTGGTAGTCCACGCCGTAAACGATGAGTGCTAAGTGTTAG<br>GGGGTTTCCGCCCCTTAGTGCTGCAGCTAACGCATTAAGCACTCCGCCTGGGGAGTACGGTCGCAAGACTGAAAC<br>TCAAAGGAATTGACGGGGACCCGCACAAGCGGTGGAGCATGTGGTTTAATTCGAAGCAACGCGAAGAACCTTAC<br>CAAATCTTGACATCCTTTGACCGCTCTAGAGATAGAGTTTTCCTCTTCGGAGGACAAAGTGACAGGTGGTGCATGG<br>TTGTCGTCAGCTCGTGTCGTGAGATGTTGGGTTAAGTCCCGCAACGAGCGCAACCCTTGAACTTAGTTGCCATCAT<br>TCAGTTGGGCACTCTAAGTTGACTGCCGGTGACAAACCGGAGGAAGGTGGGGATGACGTCAAATCATCATGCCCC<br>TTATGATTTGGGCTACACACGTGCTACAATGGACAATACAAAGGGCAGCGAAACCGCGAGGTCAAGCAAATCCCA<br>TAAAGTTGTTCTCAGTTCGGATTGTAGTCTGCAACTCGACTACATGAAGCTGGAATCGCTAGTAATCGTAGATCAG<br>CATGCTACGGTGAATACGTTCCCGGGTCTTGTACACACCGCCCGTCACACCACGAGAGTTTGTAACACCCGAAGC	100	*Staphylococcus pseudintermedius*
CTCAGGACGAACGCTGGCGGCGTGCTTAACACATGCAAGTCGAACGGAAAGGCCATGCTTGCGGGGTGCTCGAGT<br>GGCGAACGGGTGAGTAACACGTGGGTGATCTGCCTCGTACTTCGGGATAAGCTTGGGAAACTGGGTCTAATACCG<br>GATAGGACCATCATTTAGTGTTGGTGGTGGAAAGTTTTTCGGTGCGAGATGAGCCCGCGGCCTATCAGCTTGTTG<br>GTGGGGTAATGGCCTACCAAGGCGTCGACGGGTAGCCGGCCTGAGAGGGTGGACGGCCACATTGGGACTGAGAT<br>ACGGCCCAGACTCCTACGGGAGGCAGCAGTGGGGAATATTGCACAATGGGCGCAAGCCTGATGCAGCGACGCCG<br>CGTGGGGGATGACGGCCTTCGGGTTGTAAACCTCTTTCGGTAGGGACGAAGCCTTTTTGGTGACGGTACCTGTAGA<br>AGAAGCACCGGCTAACTACGTGCCAGCAGCCGCGGTAATACGTAGGGTGCGAGCGTTGTCCGGAATTACTGGGCG<br>TAAAGAGCTCGTAGGTGGTTTGTTGCGTCGTCTGTGAAATACCGGGGCTTAACTTCGGGGTTGCAGGCGATACGG<br>GCATAACTGGAGTGCTGTAGGGGAGACTGGAATTCCTGGTGTAGCGGTGGAATGCGCAGATATCAGGAGGAACAC<br>CGATGGCGAAGGCAGGTCTCTGGGCAGTAACTGACGCTGAGGAGCGAAAGCATGGGGAGCGAACAGGATTAGAT<br>ACCCTGGTAGTCCATGCTGTAAACGGTGGGCGCTAGGTGTAGGGGTCTTCCACGATTTCTGTGCCGTAGCTAACGC<br>ATTAAGCGCCCCGCCTGGGGAGTACGGCCGCAAGGCTAAAACTCAAAGGAATTGACGGGGGCCCGCACAAGCGG<br>CGGAGCATGTGGATTAATTCGATGCAACGCGAAGAACCTTACCTGGGCTTGACATACACCAGATCGCTGCAGAGA<br>TGTAGTTTCCCTTTGTGGTTGGTGTACAGGTGGTGCATGGTTGTCGTCAGCTCGTGTCGTGAGATGTTGGGTTAAGT<br>CCCGCAACGAGCGCAACCCTTGTCTTATGTTGCCAGCACGTGGTGGTGGGGACTCATGAGAGACTGCCGGGGTTA<br>ACTCGGAGGAAGGTGGGGATGACGTCAAATCATCATGCCCCTTACGTCCAGGGCTTCACACATGCTACAATGGTC<br>GGTACAACGCGTTGCGAGCCTGTGAGGGTGAGCGAATCGCTGAAAGCCGGCCTCAGTTCGGATTGGGGTCTGCAA<br>CTCGACCCCATGAAGTCGGAGTCGCTAGTAATCGCAGATCAGCAACGCTGCGGTGAATACGTTCCCGGGCCTTGT<br>ACACACCGCCCGTCACGTCATGAAAGTTGGTAACACCCGAAGCCCATGGCCTAACCCCGTTGTGGAGGGAGTGGT<br>CTAAAGTGGGATAGTCGATAGTGA	98.62	*Corynebacterium mustelae*

CTCAGATTGAACGCTGGCGGCAGGCCTAACACATGCAAGTCGAGCGGATGAAGGGAGCTTGCTCCTGGATTCAGC<br>GGCGGACGGGTGAGTAATGCCTAGGAATCTGCCTGGTAGTGGGGGATAACGTCCGGAAACGGGCGCTAATACCG<br>CATACGTCCTGAGGGAGAAAGTGGGGGATCTTCGGACCTCACGCTATCAGATGAGCCTAGGTCGGATTAGCTAGT<br>TGGTGGGGTAAAGGCCTACCAAGGCGACGATCCGTAACTGGTCTGAGAGGATGATCAGTCACACTGGAACTGAGA<br>CACGGTCCAGACTCCTACGGGAGGCAGCAGTGGGGAATATTGGACAATGGGCGAAAGCCTGATCCAGCCATGCC<br>GCGTGTGTGAAGAAGGTCTTCGGATTGTAAAGCACTTTAAGTTGGGAGGAAGGGCAGTAAGTTAATACCTTGCTG<br>TTTTGACGTTACCAACAGAATAAGCACCGGCTAACTTCGTGCCAGCAGCCGCGGTAATACGAAGGGTGCAAGCGT<br>TAATCGGAATTACTGGGCGTAAAGCGCGCGTAGGTGGTTCAGCAAGTTGGATGTGAAATCCCCGGGCTCAACCTG<br>GGAACTGCATCCAAAACTACTGAGCTAGAGTACGGTAGAGGGTGGTGGAATTTCCTGTGTAGCGGTGAAATGCGT<br>AGATATAGGAAGGAACACCAGTGGCGAAGGCGACCACCTGGACTGATACTGACACTGAGGTGCGAAAGCGTGGG<br>GAGCAAACAGGATTAGATACCCTGGTAGTCCACGCCGTAAACGATGTCGACTAGCCGTTGGGATCCTTGAGATCT<br>TAGTGGCGCAGCTAACGCGATAAGTCGACCGCCTGGGGAGTACGGCCGCAAGGTTAAAACTCAAATGAATTGAC<br>GGGGGCCCGCACAAGCGGTGGAGCATGTGGTTTAATTCGAAGCAACGCGAAGAACCTTACCTGGCCTTGACATGC<br>TGAGAACTTTCCAGAGATGGATTGGTGCCTTCGGGAACTCAGACACAGGTGCTGCATGGCTGTCGTCAGCTCGTGT<br>CGTGAGATGTTGGGTTAAGTCCCGTAACGAGCGCAACCCTTGTCCTTAGTTACCAGCACCTCGGGTGGGCACTCTA<br>AGGAGACTGCCGGTGACAAACCGGAGGAAGGTGGGGATGACGTCAAGTCATCATGGCCCTTACGGCCAGGGCTA<br>CACACGTGCTACAATGGTCGGTACAAAGGGTTGCCAAGCCGCGAGGTGGAGCTAATCCCATAAAACCGATCGTAG<br>TCCGGATCGCAGTCTGCAACTCGACTGCGTGAAGTCGGAATCGCTAGTAATCGTGAATCAGAATGTCACGGTGAA<br>TACGTTCCCGGGCCTTGTACACACCGCCCGTCACACCATGGGAGTGGGTTGCTCCAGAAGTAGCTAGTCTAACCG	100	*Pseudomonas aeruginosa*
CTCAGATTGAACGCTGGCGGCAGGCCTAACACATGCAAGTCGAACGGTAACAGGAATCAGCTTGCTGATTCGCTG<br>ACGAGTGGCGGACGGGTGAGTAATGTCTGGGAAACTGCCTGATGGAGGGGGATAACTACTGGAAACGGTAGCTA<br>ATACCGCATAACGTCGCAAGACCAAAGAGGGGGACCTTCGGGCCTCTTGCCATCGGATGTGCCCAGATGGGATTA<br>GCTAGTAGGTGGGGTAACGGCTCACCTAGGCGACGATCCCTAGCTGGTCTGAGAGGATGACCAGCCACACTGGAA<br>CTGAGACACGGTCCAGACTCCTACGGGAGGCAGCAGTGGGGAATATTGCACAATGGGCGCAAGCCTGATGCAGC<br>CATGCCGCGTGTATGAAGAAGGCCTTCGGGTTGTAAAGTACTTTCAGCGGGGAGGAAGGGAGTAAAGTTAATACC<br>TTTGCTCATTGACGTTACCCGCAGAAGAAGCACCGGCTAACTCCGTGCCAGCAGCCGCGGTAATACGGAGGGTGC<br>AAGCGTTAATCGGAATTACTGGGCGTAAAGCGCACGCAGGCGGTTTGTTAAGTCAGATGTGAAATCCCCGGGCTC<br>AACCTGGGAACTGCATCTGATACTGGCAAGCTTGAGTCTCGTAGAGGGGGGTAGAATTCCAGGTGTAGCGGTGAA<br>ATGCGTAGAGATCTGGAGGAATACCGGTGGCGAAGGCGGCCCCCTGGACGAAGACTGACGCTCAGGTGCGAAAG<br>CGTGGGGAGCAAACAGGATTAGATACCCTGGTAGTCCACGCCGTAAACGATGTCGACTTGGAGGTTGTGCCCTTG<br>AGGCGTGGCTTCCGGAGCTAACGCGTTAAGTCGACCGCCTGGGGAGTACGGCCGCAAGGTTAAAACTCAAATGAA<br>TTGACGGGGGCCCGCACAAGCGGTGGAGCATGTGGTTTAATTCGATGCAACGCGAAGAACCTTACCTGGTCTTGA<br>CATCCACAGAACTTTCCAGAGATGGATTGGTGCCTTCGGGAACTGTGAGACAGGTGCTGCATGGCTGTCGTCAGC<br>TCGTGTTGTGAAATGTTGGGTTAAGTCCCGCAACGAGCGCAACCCTTATCCTTTGTTGCCAGCGGTCCGGCCGGGA<br>ACTCAAAGGAGACTGCCAGTGATAAACTGGAGGAAGGTGGGGATGACGTCAAGTCATCATGGCCCTTACGACCA<br>GGGCTACACACGTGCTACAATGGCGCATACAAAGAGAAGCGACCTCGCGAGAGCAAGCGGACCTCATAAAGTGC<br>GTCGTAGTCCGGATTGGAGTCTGCAACTCGACTCCATGAAGTCGGAATCGCTAGTAATCGTGGATCAGAATGCCA<br>CGGTGAATACGTTCCCGGGCCTTGTACACACCGCCCGTCACACCATGGGAGTGGGTTGCAAAAGAAGTAGGTAGC	100	*Escherichia coli*

CTCAGGATGAACGCTGGCGGCGTGCCTAATACATGCAAGTCGAGCGAACAGATAAGGAGCTTGCTCCTTTGACGT<br>TAGCGGCGGACGGGTGAGTAACACGTGGGTAACCTACCTATAAGACTGGAATAACTCCGGGAAACCGGGGCTAA<br>TGCCGGATAACATGTTGAACCGCATGGTTCTACAGTGAAAGACGGTCTTGCTGTCACTTATAGATGGACCCGCGC<br>CGTATTAGCTAGTTGGTGGGGTAACGGCCTACCAAGGCGACGATACGTAGCCGACCTGAGAGGGTGATCGGCCAC<br>ACTGGAACTGAGACACGGTCCAGACTCCTACGGGAGGCAGCAGTAGGGAATCTTCCGCAATGGGCGAAAGCCTG<br>ACGGAGCAACGCCGCGTGAGTGATGAAGGTCTTCGGATCGTAAAGCTCTGTTGTTAGGGAAGAACAAATGTGTAA<br>GTAACTGTGCACATCTTGACGGTACCTAACCAGAAAGCCACGGCTAACTACGTGCCAGCAGCCGCGGTAATACGT<br>AGGTGGCAAGCGTTATCCGGAATTATTGGGCGTAAAGCGCGCGTAGGCGGTTTTTTAAGTCTGATGTGAAAGCCC<br>ACGGCTCAACCGTGGAGGGTCATTGGAAACTGGAAAACTTGAGTGCAGAAGAGGAAAGTGGAATTCCATGTGTAG<br>CGGTGAAATGCGCAGAGATATGGAGGAACACCAGTGGCGAAGGCGGCTTTCTGGTCTGCAACTGACGCTGATGTG<br>CGAAAGCGTGGGGATCAAACAGGATTAGATACCCTGGTAGTCCACGCCGTAAACGATGAGTGCTAAGTGTTAGGG<br>GGTTTCCGCCCCTTAGTGCTGCAGCTAACGCATTAAGCACTCCGCCTGGGGAGTACGGTCGCAAGACTGAAACTC<br>AAAGGAATTGACGGGGACCCGCACAAGCGGTGGAGCATGTGGTTTAATTCGAAGCAACGCGAAGAACCTTACCA<br>AATCTTGACATCCTTTGACCGCTCTAGAGATAGAGTTTTCCTCTTCGGAGGACAAAGTGACAGGTGGTGCATGGTT<br>GTCGTCAGCTCGTGTCGTGAGATGTTGGGTTAAGTCCCGCAACGAGCGCAACCCTTGAACTTAGTTGCCATCATTC<br>AGTTGGGCACTCTAAGTTGACTGCCGGTGACAAACCGGAGGAAGGTGGGGATGACGTCAAATCATCATGCCCCTT<br>ATGATTTGGGCTACACACGTGCTACAATGGACAATACAAAGGGCAGCGAAACCGCGAGGTCAAGCAAATCCCAT<br>AAAGTTGTTCTCAGTTCGGATTGTAGTCTGCAACTCGACTACATGAAGCTGGAATCGCTAGTAATCGTAGATCAGC<br>ATGCTACGGTGAATACGTTCCCGGGTCTTGTACACACCGCCCGTCACACCACGAGAGTTTGTAACACCCGAAGCC	100	*Staphylococcus pseudintermedius*
CTCAGATTGAACGCTGGCGGCAGGCTTAACACATGCAAGTCGAACGAGGTTAGAAAGCTTGCTTTTGATACCTAG<br>TGGCGGACGGGTGAGTAATGCTTAGGAATCTGCCTAGTAGTGGGGGATAACTTGGGGAAACTCAAGCTAATACCG<br>CATACGACCTACGGGTGAAAGGGGGCGCAAGCTCTCGCTATTAGATGAGCCTAAGTCGGATTAGCTAGTTGGTGG<br>GGTAAAGGCCTACCAAGGCGACGATCTGTAGCTGGTCTGAGAGGATGATCAGCCACACTGGGACTGAGACACGG<br>CCCAGACTCCTACGGGAGGCAGCAGTGGGGAATATTGGACAATGGGCGAAAGCCTGATCCAGCCATGCCGCGTG<br>TGTGAAGAAGGCCTTTTGGTTGTAAAGCACTTTAAGTGGGGAGGAAAAGTTAATGGTTAATACCCATTAGCCCTGA<br>CGTTACCCACAGAATAAGCACCGGCTAACTCTGTGCCAGCAGCCGCGGTAATACAGAGGGTGCAAGCGTTAATCG<br>GAATTACTGGGCGTAAAGCGCGCGTAGGTGGTTACTTAAGTCAGATGTGAAAGCCCCGGGCTTAACCTGGGAACT<br>GCATCTGATACTGGGTAACTAGAGTAGGTGAGAGGGGAGTAGAATTCCAGGTGTAGCGGTGAAATGCGTAGAGAT<br>CTGGAGGAATACCGATGGCGAAGGCAGCTCCCTGGCATCATACTGACACTGAGGTGCGAAAGCGTGGGTAGCAA<br>ACAGGATTAGATACCCTGGTAGTCCACGCCGTAAACGATGTCTACCAGTCGTTGGGTCTTTTAAAGACTTAGTGAC<br>GCAGTTAACGCAATAAGTAGACCGCCTGGGGAGTACGGCCGCAAGGTTAAAACTCAAATGAATTGACGGGGGCC<br>CGCACAAGCGGTGGAGCATGTGGTTTAATTCGATGCAACGCGAAGAACCTTACCTGGTCTTGACATATCTAGAAT<br>CCGAAAGAGATTTCGGAGTGCCTTCGGGAATTAGAATACAGGTGCTGCATGGCTGTCGTCAGCTCGTGTCGTGAG<br>ATGTTGGGTTAAGTCCCGCAACGAGCGCAACCCTTTTCCTTAGTTACCAGCACCTCGGGTGGGAACTCTAAGGAT<br>ACTGCCAGTGACAAACTGGAGGAAGGCGGGGACGACGTCAAGTCATCATGGCCCTTACGACCAGGGCTACACAC<br>GTGCTACAATGGTAGGTACAAAGGGTTGCTACACAGCGATGTGATGCCAATCTCAAAAAGCCTATCGTAGTCCGG<br>ATTGGAGTCTGCAACTCGACTCCATGAAGTCGGAATCGCTAGTAATCGCAGATCAGAATGCTGCGGTGAATACGT<br>TCCCGGGCCTTGTACACACCGCCCGTCACACCATGGGAGTTGATCTCACCAGAAGTGGGTAGCCTAACGCAAGAG<br>GGCGCTCACCACGGTGGGGTCGATGACTGGGGTGAAGTCGT	99	*Moraxella canis*

CTCAGATTGAACGCTGGCGGCAGGCCTAACACATGCAAGTCGAACGGTAACAGGAAGCAGCTTGCTGCTTCGCTG<br>ACGAGTGGCGGACGGGTGAGTAATGTCTGGGAAACTGCCTGATGGAGGGGGATAACTACTGGAAACGGTAGCTA<br>ATACCGCATAACGTCGCAAGACCAAAGAGGGGGACCTTCGGGCCTCTTGCCATCGGATGTGCCCAGATGGGATTA<br>GCTAGTTGGTGGGGTAACGGCTCACCAAGGCGACGATCCCTAGCTGGTCTGAGAGGATGACCAGCCACACTGGAA<br>CTGAGACACGGTCCAGACTCCTACGGGAGGCAGCAGTGGGGAATATTGCACAATGGGCGCAAGCCTGATGCAGC<br>CATGCCGCGTGTATGAAGAAGGCCTTCGGGTTGTAAAGTACTTTCAGCGGGGAGGAAGGGAGTAAAGTTAATACC<br>TTTGCTCATTGACGTTACCCGCAGAAGAAGCACCGGCTAACTCCGTGCCAGCAGCCGCGGTAATACGGAGGGTGC<br>AAGCGTTAATCGGAATTACTGGGCGTAAAGCGCACGCAGGCGGTTTGTTAAGTCAGATGTGAAATCCCCGGGCTC<br>AACCTGGGAACTGCATCTGATACTGGCAAGCTTGAGTCTCGTAGAGGGGGGTAGAATTCCAGGTGTAGCGGTGAA<br>ATGCGTAGAGATCTGGAGGAATACCGGTGGCGAAGGCGGCCCCCTGGACGAAGACTGACGCTCAGGTGCGAAAG<br>CGTGGGGAGCAAACAGGATTAGATACCCTGGTAGTCCACGCCGTAAACGATGTCGACTTGGAGGTTGTGCCCTTG<br>AGGCGTGGCTTCCGGAGCTAACGCGTTAAGTCGACCGCCTGGGGAGTACGGCCGCAAGGTTAAAACTCAAATGAA<br>TTGACGGGGGCCCGCACAAGCGGTGGAGCATGTGGTTTAATTCGATGCAACGCGAAGAACCTTACCTGGTCTTGA<br>CATCCACGGAAGTTTTCAGAGATGAAAAGGTGCCTTCGGGAACCGTGAGACAGGTGCTGCATGGCTGTCGTCAGC<br>TCGTGTTGTGAAATGTTGGGTTAAGTCCCGCAACGAGCGCAACCCTTATCCTTTGTTGCCAGCGGTCCGGCCGGGA<br>ACTCAAAGGAGACTGCCAGTGATAAACTGGAGGAAGGTGGGGATGACGTCAAGTCATCATGGCCCTTACGACCA<br>GGGCTACACACGTGCTACAATGGCGCATACAAAGAGAAGCGACCTCGCGAGAGCAAGCGGACCTCATAAAGTGC<br>GTCGTAGTCCGGATTGGAGTCTGCAACTCGACTCCATGAAGTCGGAATCGCTAGTAATCGTGGATCAGAATGCCA<br>CGGTGAATACGTTCCCGGGCCTTGTACACACCGCCCGTCACACCATGGGAGTGGGTTGCAAAAGAAGTAGGTAGC	99.86	*Shigella flexneri*
CTCAGGATGAACGCTGGCGGCGTGCCTAATACATGCAAGTCGAGCGAACAGATAAGGAGCTTGCTCCTTTGACGT<br>TAGCGGCGGACGGGTGAGTAACACGTGGGTAACCTACCTATAAGACTGGAATAACTCCGGGAAACCGGGGCTAA<br>TGCCGGATAACATGTTGAACCGCATGGTTCTACAGTGAAAGACGGTCTTGCTGTCACTTATAGATGGACCCGCGC<br>CGTATTAGCTAGTTGGTGGGGTAACGGCCTACCAAGGCGACGATACGTAGCCGACCTGAGAGGGTGATCGGCCAC<br>ACTGGAACTGAGACACGGTCCAGACTCCTACGGGAGGCAGCAGTAGGGAATCTTCCGCAATGGGCGAAAGCCTG<br>ACGGAGCAACGCCGCGTGAGTGATGAAGGTCTTCGGATCGTAAAGCTCTGTTGTTAGGGAAGAACAAATGTGTAA<br>GTAACTGTGCACATCTTGACGGTACCTAACCAGAAAGCCACGGCTAACTACGTGCCAGCAGCCGCGGTAATACGT<br>AGGTGGCAAGCGTTATCCGGAATTATTGGGCGTAAAGCGCGCGTAGGCGGTTTTTTAAGTCTGATGTGAAAGCCC<br>ACGGCTCAACCGTGGAGGGTCATTGGAAACTGGAAAACTTGAGTGCAGAAGAGGAAAGTGGAATTCCATGTGTAG<br>CGGTGAAATGCGCAGAGATATGGAGGAACACCAGTGGCGAAGGCGGCTTTCTGGTCTGCAACTGACGCTGATGTG<br>CGAAAGCGTGGGGATCAAACAGGATTAGATACCCTGGTAGTCCACGCCGTAAACGATGAGTGCTAAGTGTTAGGG<br>GGTTTCCGCCCCTTAGTGCTGCAGCTAACGCATTAAGCACTCCGCCTGGGGAGTACGGTCGCAAGACTGAAACTC<br>AAAGGAATTGACGGGGACCCGCACAAGCGGTGGAGCATGTGGTTTAATTCGAAGCAACGCGAAGAACCTTACCA<br>AATCTTGACATCCTTTGACCGCTCTAGAGATAGAGTTTTCCTCTTCGGAGGACAAAGTGACAGGTGGTGCATGGTT<br>GTCGTCAGCTCGTGTCGTGAGATGTTGGGTTAAGTCCCGCAACGAGCGCAACCCTTGAACTTAGTTGCCATCATTC<br>AGTTGGGCACTCTAAGTTGACTGCCGGTGACAAACCGGAGGAAGGTGGGGATGACGTCAAATCATCATGCCCCTT<br>ATGATTTGGGCTACACACGTGCTACAATGGACAATACAAAGGGCAGCGAAACCGCGAGGTCAAGCAAATCCCAT<br>AAAGTTGTTCTCAGTTCGGATTGTAGTCTGCAACTCGACTACATGAAGCTGGAATCGCTAGTAATCGTAGATCAGC<br>ATGCTACGGTGAATACGTTCCCGGGTCTTGTACACACCGCCCGTCACACCACGAGAGTTTGTAACACCCGAAGCC	100	*Staphylococcus pseudintermedius*

CTCAGGACGAACGCTGGCGGCGTGCCTAATACATGCAAGTAGAACGCTGAGGACTGGTGCTTGCACCGGTCCAAG<br>GAGTTGCGAACGGGTGAGTAACGCGTAGGTAACCTACCTCATAGCGGGGGATAACTATTGGAAACGATAGCTAAT<br>ACCGCATAAAAGTGTTTAACACATGTTAACCATTTAAAAGGTGCAATTGCATCACTATGAGATGGACCTGCGTTGT<br>ATTAGCTAGTTGGTGAGGTAACGGCTCACCAAGGCGACGATACATAGCCGACCTGAGAGGGTGATCGGCCACACT<br>GGGACTGAGACACGGCCCAGACTCCTACGGGAGGCAGCAGTAGGGAATCTTCGGCAATGGACGGAAGTCTGACC<br>GAGCAACGCCGCGTGAGTGAAGAAGGTTTTCGGATCGTAAAGCTCTGTTGTTAGAGAAGAATGATGGTGGGAGTG<br>GAAAATCCACCATGTGACGGTAACTAACCAGAAAGGGACGGCTAACTACGTGCCAGCAGCCGCGGTAATACGTA<br>GGTCCCGAGCGTTGTCCGGATTTATTGGGCGTAAAGCGAGCGCAGGCGGTTCTTTAAGTCTGAAGTTAAAGGCAG<br>TGGCTCAACCATTGTACGCTTTGGAAACTGGAGAACTTGAGTGCAGAAGGGGAGAGTGGAATTCCATGTGTAGCG<br>GTGAAATGCGTAGATATATGGAGGAACACCGGTGGCGAAAGCGGCTCTCTGGTCTGTAACTGACGCTGAGGCTCG<br>AAAGCGTGGGGAGCAAACAGGATTAGATACCCTGGTAGTCCACGCCGTAAACGATGAGTGCTAGGTGTTAGGCCC<br>TTTCCGGGGCTTAGTGCCGGAGCTAACGCATTAAGCACTCCGCCTGGGGAGTACGACCGCAAGGTTGAAACTCAA<br>AGGAATTGACGGGGGCCCGCACAAGCGGTGGAGCATGTGGTTTAATTCGAAGCAACGCGAAGAACCTTACCAGG<br>TCTTGACATCCTCCTGACCGGTCTAGAGATAGGCTTTCCCTTCGGGGCAGGAGTGACAGGTGGTGCATGGTTGTCG<br>TCAGCTCGTGTCGTGAGATGTTGGGTTAAGTCCCGCAACGAGCGCAACCCCTATTGTTAGTTGCCATCATTAAGTT<br>GGGCACTCTAGCGAGACTGCCGGTAATAAACCGGAGGAAGGTGGGGATGACGTCAAATCATCATGCCCCTTATGA<br>CCTGGGCTACACACGTGCTACAATGGTTGGTACAACGAGTCGCAAGCCGGTGACGGCAAGCTAATCTCTTAAAGC<br>CAATCTCAGTTCGGATTGTAGGCTGCAACTCGCCTACATGAAGTCGGAATCGCTAGTAATCGCGGATCAGCACGC<br>CGCGGTGAATACGTTCCCGGGCCTTGTACACACCGCCCGTCACACCACGAGAGTTTGTAACACCCGAAGTCGGTG<br>AGGTAACCTATTAGGAGCCAGCCGCCTAAGGTGGGATAGATGATTGGGGTGAAGTC	100	*Streptococcus dysgalactiae subsp. Equisimilis*
CTCAGATTGAACGCTGGCGGCAGGCCTAACACATGCAAGTCGAACGGTAACAGGAAGCAGCTTGCTGCTTTGCTG<br>ACGAGTGGCGGACGGGTGAGTAATGTCTGGGAAACTGCCTGATGGAGGGGGATAACTACTGGAAACGGTAGCTA<br>ATACCGCATAACGTCGCAAGACCAAAGAGGGGGACCTTCGGGCCTCTTGCCATCGGATGTGCCCAGATGGGATTA<br>GCTAGTAGGTGGGGTAACGGCTCACCTAGGCGACGATCCCTAGCTGGTCTGAGAGGATGACCAGCCACACTGGAA<br>CTGAGACACGGTCCAGACTCCTACGGGAGGCAGCAGTGGGGAATATTGCACAATGGGCGCAAGCCTGATGCAGC<br>CATGCCGCGTGTATGAAGAAGGCCTTCGGGTTGTAAAGTACTTTCAGCGGGGAGGAAGGGAGTAAAGTTAATACC<br>TTTGCTCATTGACGTTACCCGCAGAAGAAGCACCGGCTAACTCCGTGCCAGCAGCCGCGGTAATACGGAGGGTGC<br>AAGCGTTAATCGGAATTACTGGGCGTAAAGCGCACGCAGGCGGTTTGTTAAGTCAGATGTGAAATCCCCGGGCTC<br>AACCTGGGAACTGCATCTGATACTGGCAAGCTTGAGTCTCGTAGAGGGGGGTAGAATTCCAGGTGTAGCGGTGAA<br>ATGCGTAGAGATCTGGAGGAATACCGGTGGCGAAGGCGGCCCCCTGGACGAAGACTGACGCTCAGGTGCGAAAG<br>CGTGGGGAGCAAACAGGATTAGATACCCTGGTAGTCCACGCCGTAAACGATGTCGACTTGGAGGTTGTGCCCTTG<br>AGGCGTGGCTTCCGGAGCTAACGCGTTAAGTCGACCGCCTGGGGAGTACGGCCGCAAGGTTAAAACTCAAATGAA<br>TTGACGGGGGCCCGCACAAGCGGTGGAGCATGTGGTTTAATTCGATGCAACGCGAAGAACCTTACCTGGTCTTGA<br>CATCCACGGAAGTTTTCAGAGATGAGAATGTGCCTTCGGGAACCGTGAGACAGGTGCTGCATGGCTGTCGTCAGC<br>TCGTGTTGTGAAATGTTGGGTTAAGTCCCGCAACGAGCGCAACCCTTATCCTTTGTTGCCAGCGGTCCGGCCGGGA<br>ACTCAAAGGAGACTGCCAGTGATAAACTGGAGGAAGGTGGGGATGACGTCAAGTCATCATGGCCCTTACGACCA<br>GGGCTACACACGTGCTACAATGGCGCATACAAAGAGAAGCGACCTCGCGAGAGCAAGCGGACCTCATAAAGTGC<br>GTCGTAGTCCGGATTGGAGTCTGCAACTCGACTCCATGAAGTCGGAATCGCTAGTAATCGTGGATCAGAATGCCA<br>CGGTGAATACGTTCCCGGGCCTTGTACACACCGCCCGTCACACCATGGGAGTGGGTTGCAAAAGAAGTAGGTAGC<br>TTAACCTTCGGGAGGGCGCTTACCACTTTGTGATTCATGACTGGGGTGAAGTC	100	*Escherichia coli*

AGATTGAACGCTGGCGGCATGCTTTACACATGCAAGTCGAACGGCAGCACGGAAGAGCTTGCTCTTTTGGTGGCG<br>AGTGGCGAACGGGTGAGTAATACATCGGAACGTACCGAGTAGTGGGGGATAACTGTCCGAAAGGATGGCTAATA<br>CCGCATATTCTCTGAGGAGGAAAGCAGGGGACCTTCGGGCCTTGCGCTATTTGAGCGGCCGATGTCTGATTAGCT<br>AGTTGGTGGGGTAAAGGCCTACCAAGGCGACGATCAGTAGCGGGTCTGAGAGGATGATCCGCCACACTGGGACT<br>GAGACACGGCCCAGACTCCTACGGGAGGCAGCAGTGGGGAATTTTGGACAATGGGGGGAACCCTGATCCAGCCA<br>TGCCGCGTGTCTGAAGAAGGCCTTCGGGTTGTAAAGGACTTTTGTCAGGGAAGAAAAGCTTGAGGTTAATACCCT<br>TGAGTGATGACGGTACCTGAAGAATAAGCACCGGCTAACTACGTGCCAGCAGCCGCGGTAATACGTAGGGTGCG<br>AGCGTTAATCGGAATTACTGGGCGTAAAGCGGGCGCAGACGGTTACTTAAGTCAGATGTGAAATCCCCGGGCTCA<br>ACCTGGGAACTGCGTTTGAAACTGGGTGACTAGAGTATGTCAGAGGGGGGTAGAATTCCACGTGTAGCAGTGAAA<br>TGCGTAGAGATGTGGAGGAATACCGATGGCGAAGGCAGCCCCCTGGGATAATACTGACGTTCATGCCCGAAAGC<br>GTGGGTAGCAAACAGGATTAGATACCCTGGTAGTCCACGCCCTAAACGATGTCAATTAGCTGTTGGGGCACTTGA<br>TGCCTTAGTAGCGTAGCTAACGCGTGAAATTGACCGCCTGGGGAGTACGGTCGCAAGATTAAAACTCAAAGGAAT<br>TGACGGGGACCCGCACAAGCGGTGGATGATGTGGATTAATTCGATGCAACGCGAAGAACCTTACCTGGTCTTGAC<br>ATGTACGGAATCCTCCAGAGACGGAGGAGTGCCTTCGGGAGCCGTAACACAGGTGCTGCATGGCTGTCGTCAGCT<br>CGTGTCGTGAGATGTTGGGTTAAGTCCCGCAACGAGCGCAACCCTTGTCATTAGTTGCCATCATTAAGTTGGGCAC<br>TCTAATGAGACTGCCGGTGACAAGCCGGAGGAAGGTGGGGATGACGTCAAGTCCTCATGGCCCTTATGACCAGGG<br>CTTCACACGTCATACAATGGTCGGTACAGAGGGTAGCCAAGCCGCGAGGTGGAGCCAATCCCAGAAAACCGATC<br>GTAGTCCGGATTGCACTCTGCAACTCGAGTGCATGAAGTCGGAATCGCTAGTAATCGCAGGTCAGCATACTGCGG<br>TGAATACGTTCCCGGGTCTTGTACACACCGCCCGTCACACCATGGGAGTGGGGGATACCAGAAGTAGGTAGGGTA	100	*Neisseria animaloris*
CTCAGATTGAACGCTGGCGGCAGGCTTAACACATGCAAGTCGAACGGTAGCAGGAAGAAAGCTTGCTTTTTTTGC<br>TGACGAGTGGCGGACGGGTGAGTAATGCTTGGGAATCTGGCTTATGGAGGGGGATAACTGTGGGAAACTGCAGCT<br>AATACCGCGTATAATCGAGAGATGAAAGGGTGGGACCGTAAGGCCACTTGCCATAAGATGAGCCCAAGTGGGAT<br>TAGGTAGTTGGTGGGGTAAAGGCCTACCAAGCCTGCGATCTCTAGCTGGTCTGAGAGGATGGCCAGCCACACTGG<br>GACTGAGACACGGCCCAGACTCCTACGGGAGGCAGCAGTGGGGAATATTGCGCAATGGGGGGAACCCTGACGCA<br>GCCATGCCGCGTGAATGAAGAAGGCCTTCGGGTTGTAAAGTTCTTTCGGTGATGAGGAAGGGGTATTATTGAATA<br>GATAATATCATTGACGTTAATTACAGAAGAAGCACCGGCTAACTCCGTGCCAGCAGCCGCGGTAATACGGAGGGT<br>GCGAGCGTTAATCGGAATAACTGGGCGTAAAGGGCACGCAGGCGGACTTTTAAGTGAGATGTGAAATCCCCGAGC<br>TTAACTTGGGAACTGCATTTCAGACTGGGAGTCTAGAGTACTTTAGGGAGGGGTAGAATTCCACGTGTAGCGGTG<br>AAATGCGTAGAGATGTGGAGGAATACCGAAGGCGAAGGCAGCCCCTTGGGAATGTACTGACGCTCATGTGCGAA<br>AGCGTGGGGAGCAAACAGGATTAGATACCCTGGTAGTCCACGCTGTAAACGCTGTCGATTTGGGGATTGGACTTT<br>ATGTTTGGTGCCCGAAGCTAACGTGATAAATCGACCGCCTGGGGAGTACGGCCGCAAGGTTAAAACTCAAATGAA<br>TTGACGGGGGCCCGCACAAGCGGTGGAGCATGTGGTTTAATTCGATGCAACGCGAAGAACCTTACCTACTCTTGA<br>CATCCTAAGAAGAGCTCAGAGATGAGCTTGTGCCTTCGGGAACTTAGAGACAGGTGCTGCATGGCTGTCGTCAGC<br>TCGTGTTGTGAAATGTTGGGTTAAGTCCCGCAACGAGCGCAACCCTTATCCTTTGTTGCCAGCGATTTGGTCGGGA<br>ACTCAAAGGAGACTGCCAGTGACAAACTGGAGGAAGGTGGGGATGACGTCAAGTCATCATGGCCCTTACGAGTA<br>GGGCTACACACGTGCTACAATGGTGCATACAGAGGGCGGCGAGACGGCGACGTTGAGCGAATCTCAGAAAGTGC<br>ATCTAAGTCCGGATTGGAGTCTGCAACTCGACTCCATGAAGTCGGAATCGCTAGTAATCGCAAATCAGAATGTTG<br>CGGTGAATACGTTCCCGGGCCTTGTACACACCGCCCGTCACACCATGGGAGTGGGTTGTACCAGAAGTGGATAGC	99.8	*Pasteurella canis*

TTAGAGTTTGGAATCATGGCTCAGATTGAACGCTGGCGGCAGGCTTAACACATGCAAGTCGAGCGGAGAGAGGTA<br>GCTTGCTACTGATCTTAGCGGCGGACGGGTGAGTAATGCTTAGGAATCTGCCTATTAGTGGGGGACAACATCTCG<br>AAAGGGATGCTAATACCGCATACGTCCTACGGGAGAAAGCAGGGGATCTTCGGACCTTGCGCTAATAGATGAGCC<br>TAAGTCGGATTAGCTAGTTGGTGGGGTAAAGGCCTACCAAGGCGACGATCTGTAGCGGGTCTGAGAGGATGATCC<br>GCCACACTGGGACTGAGACACGGCCCAGACTCCTACGGGAGGCAGCAGTGGGGAATATTGGACAATGGGGGGAA<br>CCCTGATCCAGCCATGCCGCGTGTGTGAAGAAGGCCTTATGGTTGTAAAGCACTTTAAGCGAGGAGGAGGCTACT<br>TTAGTTAATACCTAGAGATAGTGGACGTTACTCGCAGAATAAGCACCGGCTAACTCTGTGCCAGCAGCCGCGGTA<br>ATACAGAGGGTGCAAGCGTTAATCGGATTTACTGGGCGTAAAGCGCGCGTAGGCGGCTAATTAAGTCAAATGTGA<br>AATCCCCGAGCTTMACTTGGGAATTGCATTCGATACTGGTTAGCTAGAGTGTGGGAGAGGATGGTAGAATTCCAG<br>GTGTAGCGGTGAAATGCGTAGAGATCTGGAGGAATACCGATGGCGAAGGCAGCCATCTGGCCTAACACTGACGCT<br>GAGGTGCGAAAAGSAWSGKGAGCAAACAGGATTAGATACCCTGGTAGTCCATGCCGTAAACGATGTCTACTAGC<br>CGTTGGGGCCTTTGAGGCTTTAGTGGCGCAGCTAACGCGATAAGTAGACCGCCTGGGGAGTACGGTCGCAAGACT<br>AAAACTCAAATGAATTGACGGGGGCCCGCACAAGCGGTGGAGCATGTGGTTTAATTCGATGCAACGCGAAGAAC<br>CTTACCTGGCCTTGACATAGTAAGAACTTTCCAGAGATGGATTGGTGCCTTCGGGAACTTACATACAGGTGCTGCA<br>TGGCTGTCGTCAGCTCGTGTCGTGAGATGTTGGGTTAAGTCCCGCAACGAGCGCAACCCTTTTCCTTATTTGCCAG<br>CGAGTAATGTCGGGAACTTTAAGGATACTGCCAGTGACAAACTGGAGGAAGGCGGGGACGACGTCAAGTCATCA<br>TGGCCCTTACGGCCAGGGCTACACACGTGCTACAATGGTCGGTACAAAGGGTTGCTACACAGCGATGTGATGCTA<br>ATCTCAAAAAGCCGATCGTAGTCCGGATTGGAGTCTGCAACTCGACTCCATGAAGTCGGAATCGCTAGTAATCGC<br>GGATCAGAATGCCGCGGTGAATACGTTCCCGGGCCTTGTACACACCGCCCGTCACACCATGGGAGTTTGTTGCAC<br>CAGAAGTAGCTAGCCTAACTGCAAAGAGGGCGGTTACCACGGTGTGGCCGATGACTG	99.46	*Acinetobacter seifertii*
GCTCAGATTGAACGCTGGCGGCAGGCCTAACACATGCAAGTCGAGCGGTAGCACAGAGAGCTTGCTCTCGGGTGA<br>CGAGCGGCGGACGGGTGAGTAATGTCTGGGAAACTGCCTGATGGAGGGGGATAACTACTGGAAACGGTAGCTAA<br>TACCGCATAACGTCGCAAGACCAAAGTGGGGGACCTTCGGGCCTCATGCCATCAGATGTGCCCAGATGGGATTAG<br>CTAGTAGGTGGGGTAACGGCTCACCTAGGCGACGATCCCTAGCTGGTCTGAGAGGATGACCAGCCACACTGGAAC<br>TGAGACACGGTCCAGACTCCTACGGGAGGCAGCAGTGGGGAATATTGCACAATGGGCGCAAGCCTGATGCAGCC<br>ATGCCGCGTGTATGAAGAAGGCCTTCGGGTTGTAAAGTACTTTCAGCGGGGAGGAAGGCGATAAGGTTAATAACC<br>TTGTCGATTGACGTTACCCGCAGAAGAAGCACCGGCTAACTCCGTGCCAGCAGCCGCGGTAATACGGAGGGTGCA<br>AGCGTTAATCGGAATTACTGGGCGTAAAGCGCACGCAGGCGGTCTGTCAAGTCGGATGTGAAATCCCCGGGCTCA<br>ACCTGGGAACTGCATTCGAAACTGGCAGGCTAGAGTCTTGTAGAGGGGGGTAGAATTCCAGGTGTAGCGGTGAAA<br>TGCGTAGAGATCTGGAGGAATACCGGTGGCGAAGGCGGCCCCCTGGACAAAGACTGACGCTCAGGTGCGAAAGC<br>GTGGGGAGCAAACAGGATTAGATACCCTGGTAGTCCACGCCGTAAACGATGTCGATTTGGAGGTTGTGCCCTTGA<br>GGCGTGGCTTCCGGAGCTAACGCGTTAAATCGACCGCCTGGGGAGTACGGCCGCAAGGTTAAAACTCAAATGAAT<br>TGACGGGGGCCCGCACAAGCGGTGGAGCATGTGGTTTAATTCGATGCAACGCGAAGAACCTTACCTGGTCTTGAC<br>ATCCACAGAACTTAGCAGAGATGCTTTGGTGCCTTCGGGAACTGTGAGACAGGTGCTGCATGGCTGTCGTCAGCT<br>CGTGTTGTGAAATGTTGGGTTAAGTCCCGCAACGAGCGCAACCCTTATCCTTTGTTGCCAGCGGTTCGGCCGGGAA<br>CTCAAAGGAGACTGCCAGTGATAAACTGGAGGAAGGTGGGGATGACGTCAAGTCATCATGGCCCTTACGACCAG<br>GGCTACACACGTGCTACAATGGCATATACAAAGAGAAGCGACCTCGCGAGAGCAAGCGGACCTCATAAAGTATG<br>TCGTAGTCCGGATTGGAGTCTGCAACTCGACTCCATGAAGTCGGAATCGCTAGTAATCGTAGATCAGAATGCTAC<br>GGTGAATACGTTCCCGGGCCTTGTACACACCGCCCGTCACACCATGGGAGTGGGTTGCAAAAGAAGTAGGTAGCT<br>TAACCTTCGGGAGGGCGCTTACCACTTTGTGATTCATGACTGGGG	100	*Klebsiella quasipneumoniae subsp. similipneumoniae*

AGATTGAACGCTGGCGGCAGGCCTAACACATGCAAGTCGAGCGGCAGCGGGAAAGTAGCTTGCTACTTTTGCCGG<br>CGAGCGGCGGACGGGTGAGTAATGCCTGGGAAATTGCCCAGTCGAGGGGGATAACAGTTGGAAACGACTGCTAA<br>TACCGCATACGCCCTACGGGGGAAAGCAGGGGACCTTCGGGCCTTGCGCGATTGGATATGCCCAGGTGGGATTAG<br>CTAGTTGGTGAGGTAATGGCTCACCAAGGCGACGATCCCTAGCTGGTCTGAGAGGATGATCAGCCACACTGGAAC<br>TGAGACACGGTCCAGACTCCTACGGGAGGCAGCAGTGGGGAATATTGCACAATGGGGGAAACCCTGATGCAGCC<br>ATGCCGCGTGTGTGAAGAAGGCCTTCGGGTTGTAAAGCACTTTCAGCGAGGAGGAAAGGTTGATGCCTAATACGT<br>ATCAACTGTGACGTTACTCGCAGAAGAAGCACCGGCTAACTCCGTGCCAGCAGCCGCGGTAATACGGAGGGTGC<br>AAGCGTTAATCGGAATTACTGGGCGTAAAGCGCACGCAGGCGGTTGGATAAGTTAGATGTGAAAGCCCCGGGCTC<br>AACCTGGGAATTGCATTTAAAACTGTCCAGCTAGAGTCTTGTAGAGGGGGGTAGAATTCCAGGTGTAGCGGTGAA<br>ATGCGTAGAGATCTGGAGGAATACCGGTGGCGAAGGCGGCCCCCTGGACAAAGACTGACGCTCAGGTGCGAAAG<br>CGTGGGGAGCAAACAGGATTAGATACCCTGGTAGTCCACGCCGTAAACGATGTCGATTTGGAGGCTGTGTCCTTG<br>AGACGTGGCTTCCGGAGCTAACGCGTTAAATCGACCGCCTGGGGAGTACGGCCGCAAGGTTAAAACTCAAATGAA<br>TTGACGGGGGCCCGCACAAGCGGTGGAGCATGTGGTTTAATTCGATGCAACGCGAAGAACCTTACCTGGCCTTGA<br>CATGTCTGGAATCCTGTAGAGATACGGGAGTGCCTTCGGGAATCAGAACACAGGTGCTGCATGGCTGTCGTCAGC<br>TCGTGTCGTGAGATGTTGGGTTAAGTCCCGCAACGAGCGCAACCCCTGTCCTTTGTTGCCAGCACGTAATGGTGGG<br>AACTCAAGGGAGACTGCCGGTGATAAACCGGAGGAAGGTGGGGATGACGTCAAGTCATCATGGCCCTTACGGCC<br>AGGGCTACACACGTGCTACAATGGCGCGTACAGAGGGCTGCAAGCTAGCGATAGTGAGCGAATCCCAAAAAGCG<br>CGTCGTAGTCCGGATCGGAGTCTGCAACTCGACTCCGTGAAGTCGGAATCGCTAGTAATCGCAAATCAGAATGTT<br>GCGGTGAATACGTTCCCGGGCCTTGTACACACCGCCCGTCACACCATGGGAGTGGGTTGCACCAGAAGTAGATAG	100	*Aeromonas hydrophila subsp. Hydrophila*
GGATGAACGCTGGCGGCGTGCCTAATACATGCAAGTCGAGCGAACAGATAAGGAGCTTGCTCCTTTGACGTTAGC<br>GGCGGACGGGTGAGTAACACGTGGGTAACCTACCTATAAGACTGGAATAACTCCGGGAAACCGGGGCTAATGCC<br>GGATAACATGTTGAACCGCATGGTTCTACAGTGAAAGACGGTCTTGCTGTCACTTATAGATGGACCCGCGCCGTAT<br>TAGCTAGTTGGTGGGGTAACGGCCTACCAAGGCGACGATACGTAGCCGACCTGAGAGGGTGATCGGCCACACTGG<br>AACTGAGACACGGTCCAGACTCCTACGGGAGGCAGCAGTAGGGAATCTTCCGCAATGGGCGAAAGCCTGACGGA<br>GCAACGCCGCGTGAGTGATGAAGGTCTTCGGATCGTAAAGCTCTGTTGTTAGGGAAGAACAAATGTGTAAGTAAC<br>TGTGCACATCTTGACGGTACCTAACCAGAAAGCCACGGCTAACTACGTGCCAGCAGCCGCGGTAATACGTAGGTG<br>GCAAGCGTTATCCGGAATTATTGGGCGTAAAGCGCGCGTAGGCGGTTTTTTAAGTCTGATGTGAAAGCCCACGGC<br>TCAACCGTGGAGGGTCATTGGAAACTGGAAAACTTGAGTGCAGAAGAGGAAAGTGGAATTCCATGTGTAGCGGTG<br>AAATGCGCAGAGATATGGAGGAACACCAGTGGCGAAGGCGGCTTTCTGGTCTGCAACTGACGCTGATGTGCGAAA<br>GCGTGGGGATCAAACAGGATTAGATACCCTGGTAGTCCACGCCGTAAACGATGAGTGCTAAGTGTTAGGGGGTTT<br>CCGCCCCTTAGTGCTGCAGCTAACGCATTAAGCACTCCGCCTGGGGAGTACGGTCGCAAGACTGAAACTCAAAGG<br>AATTGACGGGGACCCGCACAAGCGGTGGAGCATGTGGTTTAATTCGAAGCAACGCGAAGAACCTTACCAAATCTT<br>GACATCCTTTGACCGCTCTAGAGATAGAGTTTTCCTCTTCGGAGGACAAAGTGACAGGTGGTGCATGGTTGTCGTC<br>AGCTCGTGTCGTGAGATGTTGGGTTAAGTCCCGCAACGAGCGCAACCCTTGAACTTAGTTGCCATCATTCAGTTGG<br>GCACTCTAAGTTGACTGCCGGTGACAAACCGGAGGAAGGTGGGGATGACGTCAAATCATCATGCCCCTTATGATT<br>TGGGCTACACACGTGCTACAATGGACAATACAAAGGGCAGCGAAACCGCGAGGTCAAGCAAATCCCATAAAGTT<br>GTTCTCAGTTCGGATTGTAGTCTGCAACTCGACTACATGAAGCTGGAATCGCTAGTAATCGTAGATCAGCATGCTA<br>CGGTGAATACGTTCCCGGGTCTTGTACACACCGCCCGTCACACCACGAGAGTTTGTAACACCCGAAGCCGGTGGA	100	*Staphylococcus pseudintermedius*

GGACGAACGCTGGCGGCGTGCTTAACACATGCAAGTCGAACGGTAAGGCTCCAGCTTGCTGGGGTACACGAGTGG<br>CGAACGGGTGAGTAACACGTGGGTGACCTGCCCTGCACTTCGGGATAAGCCTGGGAAACTGGGTCTAATACCGGA<br>TAGGACCGCACCGTGAGGGTGTGGTGGAAAGTTTTTTCGGTGTGGGATGGGCCCGCGGCCTATCAGCTTGTTGGTG<br>GGGTAATGGCCTACCAAGGCGGCGACGGGTAGCCGGCCTGAGAGGGTGGACGGCCACATTGGGACTGAGACACG<br>GCCCAGACTCCTACGGGAGGCAGCAGTGGGGAATATTGCACAATGGGCGGAAGCCTGATGCAGCGACGCCGCGT<br>GGGGGATGACGGCCTTCGGGTTGTAAACTCCTTTCACCATCGACGAAGGGTTTCTGACGGTAGATGGAGAAGAAG<br>CACCGGCTAACTACGTGCCAGCAGCCGCGGTAATACGTAGGGTGCGAGCGTTGTCCGGAATTACTGGGCGTAAAG<br>AGCTCGTAGGTGGTTTGTCGCGTCGTCTGTGAAATTCCGGGGCTTAACTCCGGGCGTGCAGGCGATACGGGCATA<br>ACTTGAGTACTGTAGGGGAGACTGGAATTCCTGGTGTAGCGGTGAAATGCGCAGATATCAGGAGGAACACCGGTG<br>GCGAAGGCGGGTCTCTGGGCAGTAACTGACGCTGAGGAGCGAAAGCATGGGGAGCGAACAGGATTAGATACCCT<br>GGTAGTCCATGCCGTAAACGGTGGGCGCTAGGTGTGGGTTTCCTTCCACGGGATCCGTGCCGTAGCTAACGCATT<br>AAGCGCCCCGCCTGGGGAGTACGGCCGCAAGGCTAAAACTCAAAGGAATTGACGGGGGCCCGCACAAGCGGCGG<br>AGCATGTGGATTAATTCGATGCAACGCGAAGAACCTTACCTGGGCTTGACATATACAGGATCGCGCCAGAGATGG<br>TGTTTCCCTTGTGGCTTGTATACAGGTGGTGCATGGTTGTCGTCAGCTCGTGTCGTGAGATGTTGGGTTAAGTCCCG<br>CAACGAGCGCAACCCTTGTCTTATGTTGCCAGCACGTTGTGGTGGGGACTCGTAAGAAACTGCCGGGGTTAACTC<br>GGAGGAAGGTGGGGATGACGTCAAATCATCATGCCCCTTATGTCCAGGGCTTCACACATGCTACAATGGTCGGTA<br>CAGTGGGTTGCCAGTCCGTGAGGGCGAGCTAATCCCGCAAAGCCGGTCTCAGTTCGGATCGGGGTCTGCAACTCG<br>ACCCCGTGAAGTCGGAGTCGCTAGTAATCGCAGATCAGCAACGCTGCGGTGAATACGTTCCCGGGCCTTGTACAC<br>ACCGCCCGTCACGTCATGAAAGTCGGTAACACCCGAAGCCAGTGGCCTAACACATTTTTGTGGGGGAGCTGTCG<br>AAGGTGGGATTGGCGATTGGGACGAAGTCGTA	100	*Corynebacterium jeikeium*
AGGACGAACGCTGGCGGCGTGCCTAATACATGCAAGTCGAACGCCTTTTCTTTCACCGGAGCTTGCTCCACCGAA<br>AGAAAAGGAGTGGCGAACGGGTGAGTAACACGTGGGTAACCTGCCCATCAGAAGGGGATAACACTTGGAAACAG<br>GTGCTAATACCGTATAACAATAGAAACCGCATGGTTTCTATTTGAAAGGCGCTTTTGCGTCACTGGTGGATGGACC<br>CGCGGTGCATTAGCTAGTTGGTGAGGTAACGGCTCACCAAGGCAACGATGCATAGCCGACCTGAGAGGGTGATCG<br>GCCACATTGGGACTGAGACACGGCCCAAACTCCTACGGGAGGCAGCAGTAGGGAATCTTCGGCAATGGACGAAA<br>GTCTGACCGAGCAACGCCGCGTGAGTGAAGAAGGTTTTCGGATCGTAAAACTCTGTTGTTAGAGAAGAACAAGGA<br>TGAGAGTAGAATGTTCATCCCTTGACGGTATCTAACCAGAAAGCCACGGCTAACTACGTGCCAGCAGCCGCGGTA<br>ATACGTAGGTGGCAAGCGTTGTCCGGATTTATTGGGCGTAAAGCGAGCGCAGGCGGTTTCTTAAGTCTGATGTGA<br>AAGCCCCCGGCTCAACCGGGGAGGGTCATTGGAAACTGGGAAACTTGAGTGCAGAAGAGGAGAGTGGAATTCCA<br>TGTGTAGCGGTGAAATGCGTAGATATATGGAGGAACACCAGTGGCGAAGGCGGCTCTCTGGTCTGTAACTGACGC<br>TGAGGCTCGAAAGCGTGGGGAGCAAACAGGATTAGATACCCTGGTAGTCCACGCCGTAAACGATGAGTGCTAAGT<br>GTTGGAGGGTTTCCGCCCTTCAGTGCTGCAGCTAACGCATTAAGCACTCCGCCTGGGGAGTACGACCGCAAGGTT<br>GAAACTCAAAGGAATTGACGGGGGCCCGCACAAGCGGTGGAGCATGTGGTTTAATTCGAAGCAACGCGAAGAAC<br>CTTACCAGGTCTTGACATCCTTTGACCACTCTAGAGATAGAGCTTCCCCTTCGGGGGCAAAGTGACAGGTGGTGC<br>ATGGTTGTCGTCAGCTCGTGTCGTGAGATGTTGGGTTAAGTCCCGCAACGAGCGCAACCCTTATTGTTAGTTGCCA<br>TCATTTAGTTGGGCACTCTAGCGAGACTGCCGGTGACAAACCGGAGGAAGGTGGGGATGACGTCAAATCATCATG<br>CCCCTTATGACCTGGGCTACACACGTGCTACAATGGGAAGTACAACGAGTCGCGAAGTCGCGAGGCTAAGCTAAT<br>CTCTTAAAGCTTCTCTCAGTTCGGATTGTAGGCTGCAACTCGCCTACATGAAGCCGGAATCGCTAGTAATCGCGGA<br>TCAGCACGCCGCGGTGAATACGTTCCCGGGCCTTGTACACACCGCCCGTCACACCACGAGAGTTTGTAACACCCG	99.39	*Enterococcus raffinosus*

CTCAGATTGAACGCTGGCGGCAGGCCTAACACATGCAAGTCGAACGGTAACAGGAAGCAGCTTGCTTCTTTGCTG<br>ACGAGTGGCGGACGGGTGAGTAATGTCTGGGAAACTGCCTGATGGAGGGGGATAACTACTGGAAACGGTAGCTA<br>ATACCGCATAACGTCGCAAGACCAAAGAGGGGGACCTTCGGGCCTCTTGCCATCGGATGTGCCCAGATGGGATTA<br>GCTAGTAGGTGGGGTAACGGCTCACCTAGGCGACGATCCCTAGCTGGTCTGAGAGGATGACCAGCCACACTGGAA<br>CTGAGACACGGTCCAGACTCCTACGGGAGGCAGCAGTGGGGAATATTGCACAATGGGCGCAAGCCTGATGCAGC<br>CATGCCGCGTGTATGAAGAAGGCCTTCGGGTTGTAAAGTACTTTCAGCGGGGAGGAAGGGAGTAAAGTTAATACC<br>TTTGCTCATTGACGTTACCCGCAGAAGAAGCACCGGCTAACTCCGTGCCAGCAGCCGCGGTAATACGGAGGGTGC<br>AAGCGTTAATCGGAATTACTGGGCGTAAAGCGCACGCAGGCGGTTTGTTAAGTCAGATGTGAAATCCCCGGGCTC<br>AACCTGGGAACTGCATCTGATACTGGCAAGCTTGAGTCTCGTAGAGGGGGGTAGAATTCCAGGTGTAGCGGTGAA<br>ATGCGTAGAGATCTGGAGGAATACCGGTGGCGAAGGCGGCCCCCTGGACGAAGACTGACGCTCAGGTGCGAAAG<br>CGTGGGGAGCAAACAGGATTAGATACCCTGGTAGTCCACGCCGTAAACGATGTCGACTTGGAGGTTGTGCCCTTG<br>AGGCGTGGCTTCCGGAGCTAACGCGTTAAGTCGACCGCCTGGGGAGTACGGCCGCAAGGTTAAAACTCAAATGAA<br>TTGACGGGGGCCCGCACAAGCGGTGGAGCATGTGGTTTAATTCGATGCAACGCGAAGAACCTTACCTGGTCTTGA<br>CATCCACGGAAGTTTTCAGAGATGAAAATGTGCCTTCGGGAACCGTGAGACAGGTGCTGCATGGCTGTCGTCAGC<br>TCGTGTTGTGAAATGTTGGGTTAAGTCCCGCAACGAGCGCAACCCTTATCCTTTGTTGCCAGCGGTCCGGCCGGGA<br>ACTCAAAGGAGACTGCCAGTGATAAACTGGAGGAAGGTGGGGATGACGTCAAGTCATCATGGCCCTTACGACCA<br>GGGCTACACACGTGCTACAATGGCGCATACAAAGAGAAGCGACCTCGCGAGAGCAAGCGGACCTCATAAAGTGC<br>GTCGTAGTCCGGATTGGAGTCTGCAACTCGACTCCATGAAGTCGGAATCGCTAGTAATCGTGGATCAGAATGCCA<br>CGGTGAATACGTTCCCGGGCCTTGTACACACCGCCCGTCACACCATGGGAGTGGGTTGCAAAAGAAGTAGGTAGC<br>TTAACCTTCGGGAGGGCGCTTACCACTTTGTGATTCATGACTGGGGTGAAGTCGT	99.93	*Escherichia coli*
CTCAGGATGAACGCTAGCGGGAGGCCTAACACATGCAAGCCGAGCGGTAGATTACTTTCGGGTAATTGAGAGCGG<br>CGTACGGGTGCGGAACACGTGTGCAACCTGCCTTTATCAGGGGGATAGCCTTTCGAAAGGAAGATTAATACCCCA<br>TAATATATTATTCGGCATCGGGTGATATTGAAAACTACGGTGGATAGAGATGGGCACGCGCAAGATTAGCTAGTT<br>GGTGAGGTAACGGCTCACCAAGGCGACGATCTTTAGGGGGCCTGAGAGGGTGATCCCCCACACTGGTACTGAGAC<br>ACGGACCAGACTCCTACGGGAGGCAGCAGTGAGGAATATTGGACAATGGGTGGAAGCCTGATCCAGCCATCCCG<br>CGTGCAGGAAGACGGCCCTATGGGTTGTAAACTGCTTTTATCTGGGGATAAACCTACTTACGTGTAAGTAGCTGAA<br>GGTACCAGAAGAATAAGCACCGGCTAACTCCGTGCCAGCAGCCGCGGTAATACGGAGGGTGCAAGCGTTATCCG<br>GATTTATTGGGTTTAAAGGGTCCGTAGGCGGACTGATAAGTCAGTGGTGAAATCCGACAGCTTAACTGTCGAACT<br>GCCATTGATACTGTTAGTCTTGAGTAAGGTTGAAGTGGCTGGAATAAGTAGTGTAGCGGTGAAATGCATAGATATT<br>ACTTAGAACACCAATTGCGAAGGCAGGTCACTAAGTCTTAACTGACGCTGATGGACGAAAGCGTGGGGAGCGAA<br>CAGGATTAGATACCCTGGTAGTCCACGCCGTAAACGATGATTACTCGTTTTTGGGTTTTATGATTCAGAGACTAAG<br>CGAAAGTGATAAGTAATCCACCTGGGGAGTACGTTCGCAAGAATGAAACTCAAAGGAATTGACGGGGGCCCGCA<br>CAAGCGGTGGAGCATGTGGTTTAATTCGATGATACGCGAGGAACCTTACCAAGACTTAAATGGGAAATGACAGAT<br>TTAGAAATAGATCCTTCTTCGGACATTTTTCAAGGTGCTGCATGGTTGTCGTCAGCTCGTGCCGTGAGGTGTTAGG<br>TTAAGTCCTGCAACGAGCGCAACCCCTGTCACTAGTTGCTAACATTAAGTTGAGGACTCTAGTGAGACTGCCTAC<br>GCAAGTAGAGAGGAAGGTGGGGATGACGTCAAATCATCACGGCCCTTACGTCTTGGGCCACACACGTGCTACAAT<br>GGCCGGTACAGAGGGCAGCTACCTAGTGATAGGATGCAAATCTCGAAAGCCGGTCTCAGTTCGGATTGGAGTCTG<br>CAACTCGACTCTATGAAGCTGGAATCGCTAGTAATCGCGCATCAGCCATGGCGCGGTGAATACGTTCCCGGGCCT<br>TGTACACACCGCCCGTCAAGCCATGGAAGCTGGGGGTACCTGAAGTCGGTGACCGTAAAAGGAGCTGCCTAGGGT	100	*Elizabethkingia anophelis subsp. Anophelis*

CTCAGATTGAACGCTAGCGGGATGCTTTACACATGCAAGTCGAACGGCAGCGCGGACTTCGGTCTGGCGGCGAGT<br>GGCGAACGGGTGAGTAATGTATCGGAACGTGCCCAGTAGCGGGGGATAACTACGCGAAAGCGTAGCTAATACCG<br>CATACGCCCTACGGGGGAAAGCAGGGGATCGTAAGACCTTGCACTATTGGAGCGGCCGATATCGGATTAGCTAGT<br>TGGTGAGGTAACGGCTCACCAAGGCGACGATCCGTAGCTGGTTTGAGAGGACGACCAGCCACACTGGGACTGAG<br>ACACGGCCCAGACTCCTACGGGAGGCAGCAGTGGGGAATTTTGGACAATGGGGGCAACCCTGATCCAGCCATCC<br>CGCGTGTGCGATGAAGGCCTTCGGGTTGTAAAGCACTTTTGGCAGGAAAGAAACGTCGCGGGTTAATACCCCGTG<br>GAACTGACGGTACCTGCAGAATAAGCACCGGCTAACTACGTGCCAGCAGCCGCGGTAATACGTAGGGTGCAAGC<br>GTTAATCGGAATTACTGGGCGTAAAGCGTGCGCAGGCGGTTCGGAAAGAAAGATGTGAAATCCCAGAGCTTAACT<br>TTGGAACTGCATTTTTAACTACCGAGCTAGAGTGTGTCAGAGGGAGGTGGAATTCCGCGTGTAGCAGTGAAATGC<br>GTAGATATGCGGAGGAACACCGATGGCGAAGGCAGCCTCCTGGGATAACACTGACGCTCATGCACGAAAGCGTG<br>GGGAGCAAACAGGATTAGATACCCTGGTAGTCCACGCCCTAAACGATGTCAACTAGCTGTTGGGGCCTTCGGGCC<br>TTGGTAGCGCAGCTAACGCGTGAAGTTGACCGCCTGGGGAGTACGGTCGCAAGATTAAAACTCAAAGGAATTGAC<br>GGGGACCCGCACAAGCGGTGGATGATGTGGATTAATTCGATGCAACGCGAAAAACCTTACCTACCCTTGACATGT<br>CTGGAATCCTGAAGAGATTTAGGAGTGCTCGCAAGAGAACCGGAACACAGGTGCTGCATGGCTGTCGTCAGCTCG<br>TGTCGTGAGATGTTGGGTTAAGTCCCGCAACGAGCGCAACCCTTGTCATTAGTTGCTACGAAAGGGCACTCTAATG<br>AGACTGCCGGTGACAAACCGGAGGAAGGTGGGGATGACGTCAAGTCCTCATGGCCCTTATGGGTAGGGCTTCACA<br>CGTCATACAATGGTCGGGACAGAGGGTCGCCAACCCGCGAGGGGGAGCCAATCCCAGAAACCCGATCGTAGTCC<br>GGATCGCAGTCTGCAACTCGACTGCGTGAAGTCGGAATCGCTAGTAATCGCGGATCAGCATGTCGCGGTGAATAC<br>GTTCCCGGGTCTTGTACACACCGCCCGTCACACCATGGGAGTGGGTTTTACCAGAAGTAGTTAGCCTAACCGCAA	99.52	*Achromobacter insolitus*
TGGCTCAGATTGAACGCTGGCGGCAGGCCTAACACATGCAAGTCGAACGGTAACAGGAAGCAGCTTGCTGTTTCG<br>CTGACGAGTGGCGGACGGGTGAGTAATGTCTGGGAAACTGCCTGATGGAGGGGGATAACTACTGGAAACGGTAG<br>CTAATACCGCATAACGTCGCAAGACCAAAGAGGGGGACCTTCGGGCCTCTTGCCATCGGATGTGCCCAGATGGGA<br>TTAGCTTGTTGGTGGGGTAACGGCTCACCAAGGCGACGATCCCTAGCTGGTCTGAGAGGATGACCAGCCACACTG<br>GAACTGAGACACGGTCCAGACTCCTACGGGAGGCAGCAGTGGGGAATATTGCACAATGGGCGCAAGCCTGATGC<br>AGCCATGCCGCGTGTATGAAGAAGGCCTTCGGGTTGTAAAGTACTTTCAGCGGGGAGGAAGGGAGTAAAGTTAAT<br>ACCTTTGCTCATTGACGTTACCCGCAGAAGAAGCACCGGCTAACTCCGTGCCAGCAGCCGCGGTAATACGGAGGG<br>TGCAAGCGTTAATCGGAATTACTGGGCGTAAAGCGCACGCAGGCGGTTTGTTAAGTCAGATGTGAAATCCCCGGG<br>CTCAACCTGGGAACTGCATCTGATACTGGCAAGCTTGAGTCTCGTAGAGGGGGGTAGAATTCCAGGTGTAGCGGT<br>GAAATGCGTAGAGATCTGGAGGAATACCGGTGGCGAAGGCGGCCCCCTGGACGAAGACTGACGCTCAGGTGCGA<br>AAGCGTGGGGAGCAAACAGGATTAGATACCCTGGTAGTCCACGCCGTAAACGATGTCGACTTGGAGGTTGTGCCC<br>TTGAGGCGTGGCTTCCGGAGCTAACGCGTTAAGTCGACCGCCTGGGGAGTACGGCCGCAAGGTTAAAACTCAAAT<br>GAATTGACGGGGGCCCGCACAAGCGGTGGAGCATGTGGTTTAATTCGATGCAACGCGAAGAACCTTACCTGGTCT<br>TGACATCCACGGAAGTTTTCAGAGATGAGAATGTGCCTTCGGGAACCGTGAGACAGGTGCTGCATGGCTGTCGTC<br>AGCTCGTGTTGTGAAATGTTGGGTTAAGTCCCGCAACGAGCGCAACCCTTATCCTTTGTTGCCAGCGGTCCGGCCG<br>GGAACTCAAAGGAGACTGCCAGTGATAAACTGGAGGAAGGTGGGGATGACGTCAAGTCATCATGGCCCTTACGA<br>CCAGGGCTACACACGTGCTACAATGGCGCATACAAAGAGAAGCGACCTCGCGAGAGCAAGCGGACCTCATAAAG<br>TGCGTCGTAGTCCGGATTGGAGTCTGCAACTCGACTCCATGAAGTCGGAATCGCTAGTAATCGTGGATCAGAATG<br>CCACGGTGAATACGTTCCCGGGCCTTGTACACACCGCCCGTCACACCATGGGAGTGGGTTGCAAAAGAAGTAGGT	100	*Escherichia coli*

ATGGCTCAGATTGAACGCTGGCGGCAGGCCTAACACATGCAAGTCGAGCGGATGAAGGGAGCTTGCTCCTGGATT<br>CAGCGGCGGACGGGTGAGTAATGCCTAGGAATCTGCCTGGTAGTGGGGGATAACGTCCGGAAACGGGCGCTAAT<br>ACCGCATACGTCCTGAGGGAGAAAGTGGGGGATCTTCGGACCTCACGCTATCAGATGAGCCTAGGTCGGATTAGC<br>TAGTTGGTGGGGTAAAGGCCTACCAAGGCGACGATCCGTAACTGGTCTGAGAGGATGATCAGTCACACTGGAACT<br>GAGACACGGTCCAGACTCCTACGGGAGGCAGCAGTGGGGAATATTGGACAATGGGCGAAAGCCTGATCCAGCCA<br>TGCCGCGTGTGTGAAGAAGGTCTTCGGATTGTAAAGCACTTTAAGTTGGGAGGAAGGGCAGTAAGTTAATACCTT<br>GCTGTTTTGACGTTACCAACAGAATAAGCACCGGCTAACTTCGTGCCAGCAGCCGCGGTAATACGAAGGGTGCAA<br>GCGTTAATCGGAATTACTGGGCGTAAAGCGCGCGTAGGTGGTTCAGCAAGTTGGATGTGAAATCCCCGGGCTCAA<br>CCTGGGAACTGCATCCAAAACTACTGAGCTAGAGTACGGTAGAGGGTGGTGGAATTTCCTGTGTAGCGGTGAAAT<br>GCGTAGATATAGGAAGGAACACCAGTGGCGAAGGCGACCACCTGGACTGATACTGACACTGAGGTGCGAAAGCG<br>TGGGGAGCAAACAGGATTAGATACCCTGGTAGTCCACGCCGTAAACGATGTCGACTAGCCGTTGGGATCCTTGAG<br>ATCTTAGTGGCGCAGCTAACGCGATAAGTCGACCGCCTGGGGAGTACGGCCGCAAGGTTAAAACTCAAATGAATT<br>GACGGGGGCCCGCACAAGCGGTGGAGCATGTGGTTTAATTCGAAGCAACGCGAAGAACCTTACCTGGCCTTGACA<br>TGCTGAGAACTTTCCAGAGATGGATTGGTGCCTTCGGGAACTCAGACACAGGTGCTGCATGGCTGTCGTCAGCTC<br>GTGTCGTGAGATGTTGGGTTAAGTCCCGTAACGAGCGCAACCCTTGTCCTTAGTTACCAGCACCTCGGGTGGGCA<br>CTCTAAGGAGACTGCCGGTGACAAACCGGAGGAAGGTGGGGATGACGTCAAGTCATCATGGCCCTTACGGCCAG<br>GGCTACACACGTGCTACAATGGTCGGTACAAAGGGTTGCCAAGCCGCGAGGTGGAGCTAATCCCATAAAACCGAT<br>CGTAGTCCGGATCGCAGTCTGCAACTCGACTGCGTGAAGTCGGAATCGCTAGTAATCGTGAATCAGAATGTCACG<br>GTGAATACGTTCCCGGGCCTTGTACACACCGCCCGTCACACCATGGGAGTGGGTTGCTCCAGAAGTAGCTAGTCT<br>AACCGCAAGGGGGACGGTTACCACGGAGTGATTCATGACTGGGGTGAAGTCGTAACA	100	*Pseudomonas aeruginosa*
